# Self-buoyant cell-spheroid culture programming multi-spheroid assembly

**DOI:** 10.1126/sciadv.aee3306

**Published:** 2026-10-07

**Authors:** Yuanhang Xiang, Binqi Wei, Yueyang Sun, Weifeng Liu, Hui Zhao, Xiaojie Qin, Changfeng Zhu, Xinchun Li, Fan Yang

**Affiliations:** ^1^ Guangxi Key Laboratory of Pharmaceutical Precision Detection and Screening, School of Pharmacy, State Key Laboratory of Targeting Oncology, Guangxi Medical University, Nanning 530021, China.; ^2^ School of Chemistry and Chemical Engineering, New Cornerstone Science Laboratory, Frontiers Science Center for Transformative Molecules, National Center for Translational Medicine, Shanghai Jiao Tong University, Shanghai, China.; ^3^ School of Chemistry and Molecular Engineering, East China Normal University, Shanghai 200241, China.; ^4^ School of Pharmacy, Nanjing Medical University, Nanjing 211166, China.; ^5^ Department of Gastroenterology and Hepatology, Zhongshan Hospital, Fudan University, Shanghai 200032, China.

## Abstract

Tempo-spatially controlled culture and assembly of cellular spheroids are fundamental in engineering tissue-like assembloids for scalable application, yet remain a technical challenge. Here, we show self-buoyant culture of cell spheroids and programmable assembly of multi-spheroid by microbubbles in a high-throughput, external field-free and miniaturized format. This approach allows one-step engineering of biocompatible cell-adhesive buoyancy interface that lifts the self-adaptive levitation growth of reliable one-drop-one-spheroid in an array without requiring any external fields. Using this self-floating spheroidal microarray, we find that a facile droplet ‘kiss’ facilitates the buoyancy-driven ultrafast transfer (∼1 s) of spheroids between top-down adjacent droplets, thus achieving rapid spheroid relocation, media exchange, and drug administration in parallel. Such a self-buoyant approach allows programmable horizontal/vertical bioassembly and enhanced fusion of homo/heterogeneous multi-spheroids into different “buoyantoid” patterns via sequential flash transfer along droplet array. This self-powered design provides a promising tool to enable mass production and smart manipulation of encoded assembloids for precision medicine, tissue engineering and high-throughput drug screening.

## INTRODUCTION

Cellular spheroids, organoids, and their assembloids are revolutionizing in vitro disease models, tissue engineering and drug discovery (*1*), which rely primarily on advanced three-dimensional (3D) cell culture technologies (*2*). For example, scaffold-free 3D culture allows self-organization of cells into spheroids or organoids (*3*), but necessitates either ultra-low attachment (ULA) surface (*4*), hanging-drop designs (*5*), or complicated external fields (e.g., acoustic field) for cell aggregation (*6*), with potential spheroidal collapse. In comparison, scaffold-based strategies use biocompatible materials, such as hydrogels (*7*–*9*), molecular glues (*10*), magnetic nanoparticles (*11*), and microparticles (*12*, *13*), to mimic extracellular matrix (ECM) for guiding cell growth, differentiation, and organ-like cohesion in a manner resembling in vivo self-organization (*8*, *14*, *15*), and also offer superior control over the mechanical and biochemical cues to improve tissue-like cytoarchitecture formation and functionality (*16*). Despite the progress, existing scaffold-based 3D culture techniques are fundamentally limited by (i) relying heavily on external fields for cell manipulation, such as magnetic levitation culture that demands complicated magnetic field configuration and endocytosis of magnetic beads or paramagnetic solution (*17*–*19*), (ii) difficulties in spheroidal fusion (*20*), ECM deposition, and exchanging media without spheroidal damaging, loss, or cross-contamination, and (iii) challenges of maintaining uniform, high-throughput single-spheroid culture, and controllable multi-spheroid assembly that dominate downstream large-scale testing performance.

Recently, hydrogel microcavity arrays have been designed at a multi-well plate bottom to enable arrayed organoid culture by gravity (*F*_g_) trapping (*21*), whereas the existence of non-formed and lost organoids during culture and media exchange degrades the advantage. Indeed, such multi-well plates-based spheroid culture is incompatible with multi-spheroid assembly (*5*), which often needs manually handling or 3D bioprinting to form assembloids (*22*–*24*), thereby largely reducing downstream testing efficiency, for example in high-throughput drug screening, and may result in physical damage of spheroids (*20*). To alleviate these issues, superhydrophobic patterning-based droplet microarrays have been created to enable high-throughput spheroid culture and transfer for 3D multicellular assembly (*5*), while reducing cross-contamination, user-to-user variation, and spheroid loss rates during handling (*25*–*27*). Despite these merits, critical bottlenecks remain unresolved that hinder their translation to programmable multi-spheroid engineering: the inherent nanoliter-scale droplet volume leads to rapid evaporation, which compromises long-term culture stability and spheroid size; frequent medium exchange is cumbersome and prone to disrupting fragile spheroids; and multi-spheroid assembly is limited in 2D. Given the growing demand for robust 3D culture platforms that integrate high-throughput single-spheroid isolation, long-term viability maintenance, and flexible multi-spheroid assembly—key prerequisites for deciphering inter-spheroid cross-talk and developing tissue-mimetic constructs—how can we overcome the challenge?

To address these hurdles, we here report a bubble-floated self-adaptive 3D cell culture strategy (BFC) to enable high-throughput one-spheroid-per-well culture and spatially programmable multi-spheroid assembly. By one-step assembling poly-L-lysine (PLL) onto biocompatible glass microbubble (GB) (*28*), we can engineer buoyant cell-adhesive GB (CGB) for guiding various cells (e.g., cancer cells) adhesion, aggregation, and 3D spatially-adapted growth to form single cellular spheroids self-floated in each droplet via dominant buoyancy (*F*_b_) of CGB (*F*_b_ > *F*_g_, Fig. 1A). Such a self-lifted BFC design can perfectly interface with droplet microarray to achieve high-throughput culture, transfer and bioassembly of cell spheroids. As kissed between bottom-up adjacent droplets, the self-buoyant cell spheroids cultured in the bottom microarray can spontaneously enter the up droplet via a droplet bridge in a flash, thus facilitating ultrafast (∼1 s), high-throughput transfer of cell spheroids, medium exchange, and drug administration (Fig. 1B). More importantly, this BFC approach allows programmable bioassembly and fusion of multi-spheroids, promoted by *F*_b_ and droplet surface tension (*F*_st_), into planar patterns (termed “buoyantoids”) across peanut, sweet heart, four-leaf clover, and Olympic rings via facile and sequential droplet kiss-bridge-separate handling (Fig. 1C). This approach also enables bioprinting-like (*29*), programmable vertical assembly of multi-spheroids within a capillary for promoted spheroid fusion, driven by *F*_b_, capillary force (*F*_c_), and Laplace pressure (*29*) in a synergy (Fig. 1D). Overall, our BFC technology offers several merits over traditional scaffold-based 3D cell culture, including (i) self-buoyant high-throughput spheroids cultivation without any external fields; (ii) buoyancy-activated single-spheroid-per-well culture with ultrafast transfer; (iii) buoyancy-promoted spheroid fusion; and (iv) programmable multi-spheroid bioassembly with consistent focal planes facilitating the downstream screening analysis, such as the study of pharmacological intervention in a tumoroid invasion model.

**Fig. 1.**
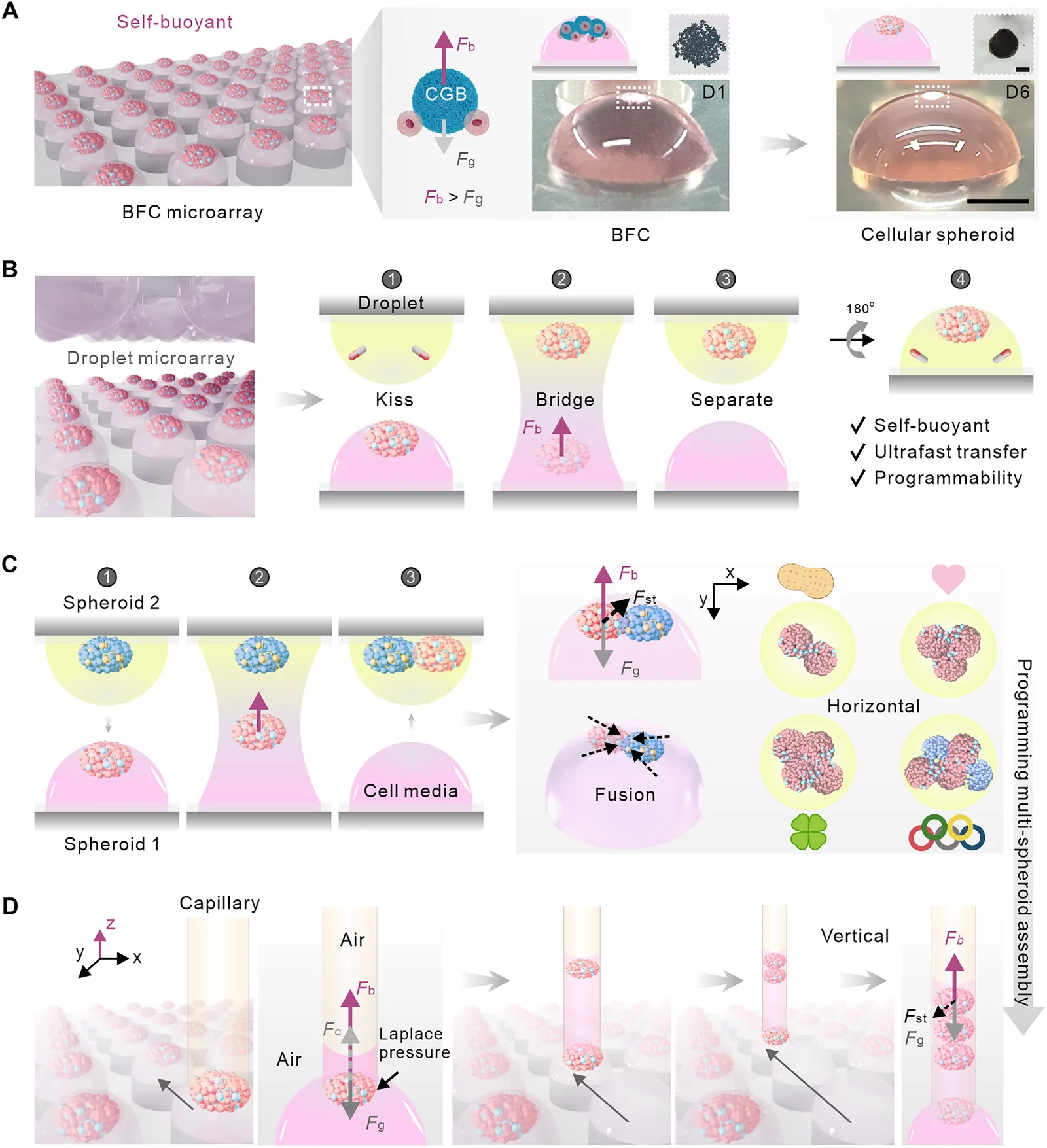
BFC-programmed multi-spheroid assembly. (**A**) In BFC microarray, buoyancy (*F*_b_)-dominated cell-adhesive glass bubble (CGB) adsorbing cells for self-buoyant and self-adaptive growth into a cellular spheroid in a droplet. Insets show a BFC spheroid at day 1 (D1) and D6, respectively. Scale bars, 300 μm (up) and 2 mm (down). (**B**) Schematic of droplet microarray-enabled high-throughput and ultrafast transfer of cellular spheroids for drug administration using a droplet sandwich method, following a ‘kiss’ between the up drug-droplet and down spheroid-droplet (1), buoyancy-activated transfer of cellular spheroid from bottom to up droplet through a droplet bridge (2), the cell-spheroid entering the upper droplet after separation (3), and 180° flipping of the upper droplet after cell-spheroid transfer (4). (**C**) BFC programming multi-spheroids horizontal assembly and fusion promoted by *F*_b_ and droplet surface tension (*F*_st_) into planar patterns across peanut, sweet heart, four-leaf clover, and Olympic rings via sequential sandwich steps (kiss, bridge and separate). (**D**) BFC-enabled bioprinting-like, programmable multi-spheroid vertical assembly and fusion through capillary-droplet interaction driven by synergic spheroid buoyancy, capillary force (*F*_c_) and Laplace pressure.

## RESULTS

### Engineering cell-laden bubbles for BFC

As emerging floating biomaterials (*30*–*32*), GB features low density (0.6 g/cm^3^), good biocompatibility, and easy-to-use in target cells capture via facile surface chemistry (*28*). Especially, GB (∼18 μm diameter) is larger than the size of human cancer cells (∼10 μm), thus may serve as a free-packed granular bed to facilitate cell movement between bubbles and aid the formation of cell spheroids (*33*), but remains elusive. To enhance cell attachment and the growth of cell spheroids, we constructed CGB by one-step assembly of GB with PLL (Fig. 2A), a polymer known for promoting interfacial cell adhesion (*34*, *35*). Such an effective self-assembly process was due mainly to the synergic electrostatic adsorption and hydrogen bonds between PLL and GB (*34*). Transmission electron microscopy (TEM) and scanning electron microscopy (SEM) both revealed the presence of assembled PLL nanolayer (∼54 nm thick) on the surface of the CGB (Fig. 2B and fig. S1). X-ray photoelectron spectroscopy (XPS) analysis showed prominent nitrogen peaks for CGB surface (Fig. 2C and fig. S2). The amino groups on the surface were also characterized using N-hydroxysuccinimide (NHS)-modified fluorescent dye (AZDye 488), and a clear green fluorescence signal was observed on the CGB surface (fig. S3), improved by 11 folds compared to GB. These results indicate the successful preparation of CGB.

**Fig. 2.**
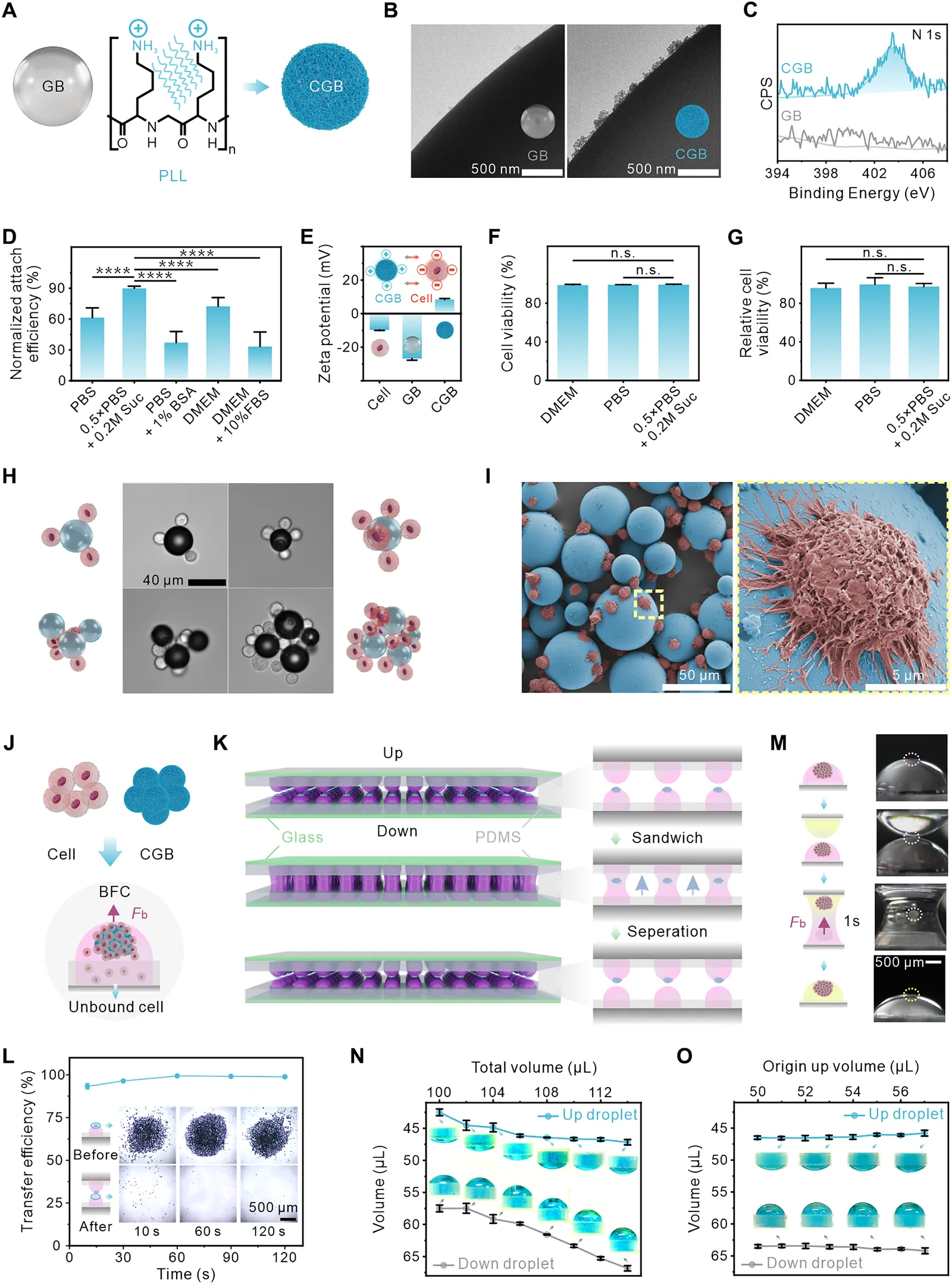
Engineering cell-laden bubbles for BFC and ultrafast sandwich transfer. (**A**) Schematic of CGB preparation via one-step assembly of PLL on GB. (**B**) TEM images of GB and CGB surfaces. (**C**) Nitrogen elemental analysis from the detailed XPS between GB and CGB. (**D**) Cell adsorption efficiency of CGB in different attachment solutions. (**E**) Measurement of Zeta potentials of HeLa cells, GB and CGB in attachment solution. Inset shows charge-based adsorption mechanisms between cells and CGB. (**F**) Calcein-AM/PI staining after 1 min exposure to DMEM, PBS, or attachment solution. (**G**) CCK-8 activity of HeLa cells cultured for an additional 2 days in complete culture medium after 1 min exposure to DMEM, PBS, or the attachment solution. (**H**) Optical microscope images show the distinct binding patterns between CGB and cells. (**I**) SEM images of cells on CGB; enlargement shows pseudopodial attachment. (**J**) Schematic of CGB adsorbing cells and self-aggregating at the apex of a droplet for BFC due to buoyancy (*F*_b_), leaving unbound cells. (**K**) High-throughput sandwich separation of cell–CGB clusters or spheroids using glass-backed PDMS multiwell chips. (**L**) Transfer efficiency of CGB–cell complexes at 10–120 s; insets show representative images before and after transfer. (**M**) Snapshots of the ultrafast sandwich transfer of a cell-spheroid. Dotted coils denote the spheroid. (**N**) Volumes of the up and down droplets after separation for different total droplet volumes. (**O**) Volumes of the up and down droplets after separation for different up droplet volumes under the same total droplet volume. Data are presented as mean ± SD (n = 5). Statistical significance was assessed using one-way ANOVA followed by Dunnett’s multiple-comparisons test, with the 0.5 × PBS + 0.2 M sucrose group as the reference condition. ****P ≤ 0.0001. n.s., *P* > 0.05.

The PLL rendered CGB surface not only nanorough, but positively charged because of abundant amino groups in PLL, thus allowing efficient adsorption of negatively charged cells (*34*). The efficiency of cell attachment was evaluated across multiple cell attachment solutions, including phosphate-buffered saline (PBS), 0.5 × PBS + 0.2 M Suc, PBS + 1% bovine serum albumin (BSA), dulbecco’s modified eagle medium (DMEM), and DMEM +10% fetal bovine serum (FBS). As a result, we found that CGB exhibited the highest cell adhesion efficiency (∼90%) in 0.5 × PBS + 0.2 M sucrose (Suc) (Fig. 2D), likely due to the relatively low ionic strength of the solution. In solutions containing abundant proteins (such as BSA or FBS), cell adsorption efficiency markedly decreased, potentially attributable to nonspecific protein adsorption onto the CGB surface (*36*), thereby diminishing the positive surface charge and reducing cellular attachment. Consequently, we selected 0.5 × PBS + 0.2 M Suc as the attachment solution for cell-CGB attaching. We next measured the Zeta potential of CGB and HeLa cells in 0.5 × PBS + 0.2 M Suc, and found that CGB exhibited positive charges, and the HeLa cells were negatively charged (Fig. 2E). This polarity difference allowed for the adsorption of cells onto CGB surface through electrostatic interactions. Following incubation of the cells with DMEM, PBS, or the attachment solution, their viability was assessed via live/dead staining (Calcein-AM/PI), revealing minimal impact on cell survival (Fig. 2F and fig. S4). Direct live/dead staining of floating cell-CGB clusters after the initial 24 h BFC culture confirmed predominant viability under the actual BFC condition (fig. S5), and a detachment assay further characterized the detached live/dead cells during this period (fig. S6). Upon transferring the treated cells to DMEM and culturing for 2 days (D2), Cell Counting Kit-8 (CCK-8) assays confirmed no significant reduction in cell viability (Fig. 2G). Additionally, in evaluating CGB cell capture over time, the peak attachment efficiency was found within 1 min (fig. S7), with a concomitant decline because of increasingly less cell-CGB collisions. Moreover, varying the PLL concentration did not notably affect attachment efficiency (fig. S8). Collectively, these findings demonstrate that CGB can achieve efficient and rapid cell adhesion under the mild condition.

To better understand cell adhesion on CGB, we mixed CGB (10^5^) with five times the number of HeLa cells in the attachment solution. Microscopic observations revealed a heterogeneous cellular adhesion pattern on the CGB surface, from three cells versus one CGB, to tens of cells versus four and more CGB (Fig. 2H and fig. S9). According to theoretical calculations (see supplementary materials and fig. S10), the cell-loading capacity of CGB increases with its volume. For instance, a 20-μm CGB can potentially carry around 40 cells, assuming each cell with a diameter of 10 μm. It is evident that individual cells can adhere to two and even more CGB, forming a cell-CGB cluster as an effective BFC precursor. Furthermore, an SEM image of the cell-CGB cluster showed that many cells attached to CGB in an aggregated or dispersed fashion, and an enlarged image clearly indicated the adhesion of dense pseudopodia of single cells onto the bubble surface (Fig. 2I and fig. S11), akin to on other cell-adhesion nanointerfaces. Moreover, CGBs varying in sizes may play complementary roles, with smaller ones facilitating dynamic rearrangement and multicellular compaction, and larger ones providing stable local anchoring surfaces for cell spreading and aggregate stabilization (*33*). These findings suggest that our CGB has great potential to adhere living cells for BFC that guides their growth, spreading, and formation of cell spheroids.

### Ultrafast sandwich transfer in BFC

The inherent buoyancy (*F*_b_) of hollow CGB allows cell-CGB to spontaneously float upward in solution and levitate at a droplet apex (Fig. 2J). By integrating with a droplet microarray, this feature thus could promote self-separation and transfer of cell-CGB without any external forces (e.g., magnetic fields or centrifugation) (Fig. 2K). To achieve this, we fabricated a droplet microarray using polydimethylsiloxane (PDMS), a common biomaterial for microfluidic cell culture (*37*), based on an inverted copper mold (fig. S12). In the microarray chip (12 × 6 wells), each culture well was designed 2 mm high and 5 mm in diameter to enable high-throughput culture and transfer (fig. S13). Specifically, a standardized microfabrication process and the same PDMS substrate ensured the uniform culture-well size (relative standard deviation, RSD = 1.98%, fig. S14), and meanwhile equal droplet volumes rendered the droplet morphology identical across the entire chip, enabling consistent cell manipulation. To prevent potential bending of the PDMS array chip in large-scale manipulation, the chip bottom was attached to a glass substrate (Fig. 2K). Such a facile design facilitated top-down droplet ‘kiss’, merging and separation via droplet sandwich (fig. S15).

Notably, this droplet sandwich method did not require any external forces to achieve high-throughput transfer in a single operation. We found that the most, if not all, cell-CGB clusters were transferred in 10 s, with a transfer efficiency >90% (Fig. 2L), which was comparable to transferring of individual CGB (fig. S16). Such a buoyancy-activated transfer strategy was further used to transport CGB-based cellular spheroids from the down droplet to the up one in a flash (∼1 s) upon droplet sandwich merging (Fig. 2M and movies S1 and S2). This result demonstrates the feasibility of using this droplet sandwich microarray for ultrafast, high-throughput cell-spheroid manipulation. In addition, we observed that the volume between the up and down droplets was inconsistent after separation due to the influence of gravity. When the same liquid volume was used for both the up and down droplet, only the down showed a clear change in volume after separation, while the up remained around 45 μL (Fig. 2N). When maintaining the total volume of the up and down droplets constant, we found that the volume of the up-down droplets became consistent after separation, regardless of the down droplet initial volume (Fig. 2O). This consistency in droplet volumes provides uniform conditions for BFC-based cell-spheroid transfer, buffer exchange, and drug administration.

### BFC-enabled cell-spheroid array culture

Using such a self-driven BFC transfer system, we performed 3D suspension culture of cervical cancer cells (HeLa) obviating the need for any external fields. Following the validation of sufficient cell loading required for robust BFC spheroid formation (fig. S17), we adjusted the initial number of CGB (from 100 to 2000), and achieved control over the size of either the cell-CGB clusters or cellular spheroids. As the number of CGBs increasing, the size of the cell clusters at day 1 (D1) or spheroids (D6) also gradually increased (Fig. 3A). Within the tested range, the projected areas between the cell–CGB clusters (D1) and spheroids (D6) showed approximately linear empirical correlations with the initial CGB input with a correlation coefficient of 0.961 (Fig. 3B). We also found, after growing for 6 days, there was a linear relationship between the cell-spheroid size and the CGB number on the D6, with a correlation coefficient of 0.955 (Fig. 3B). These results suggest that our BFC system allows not only flash transfer, but size controllable culture of cellular spheroids in parallel. Of note, the self-buoyant culture mode kept the growing spheroids suspended at the droplet apex without direct contact with the array substrate, thereby minimizing substrate-associated adhesion and enabling chip reuse that supported consistent BFC spheroid formation across different operating conditions and operators, demonstrating their practical reusability under the tested conditions (fig. S18).

**Fig. 3.**
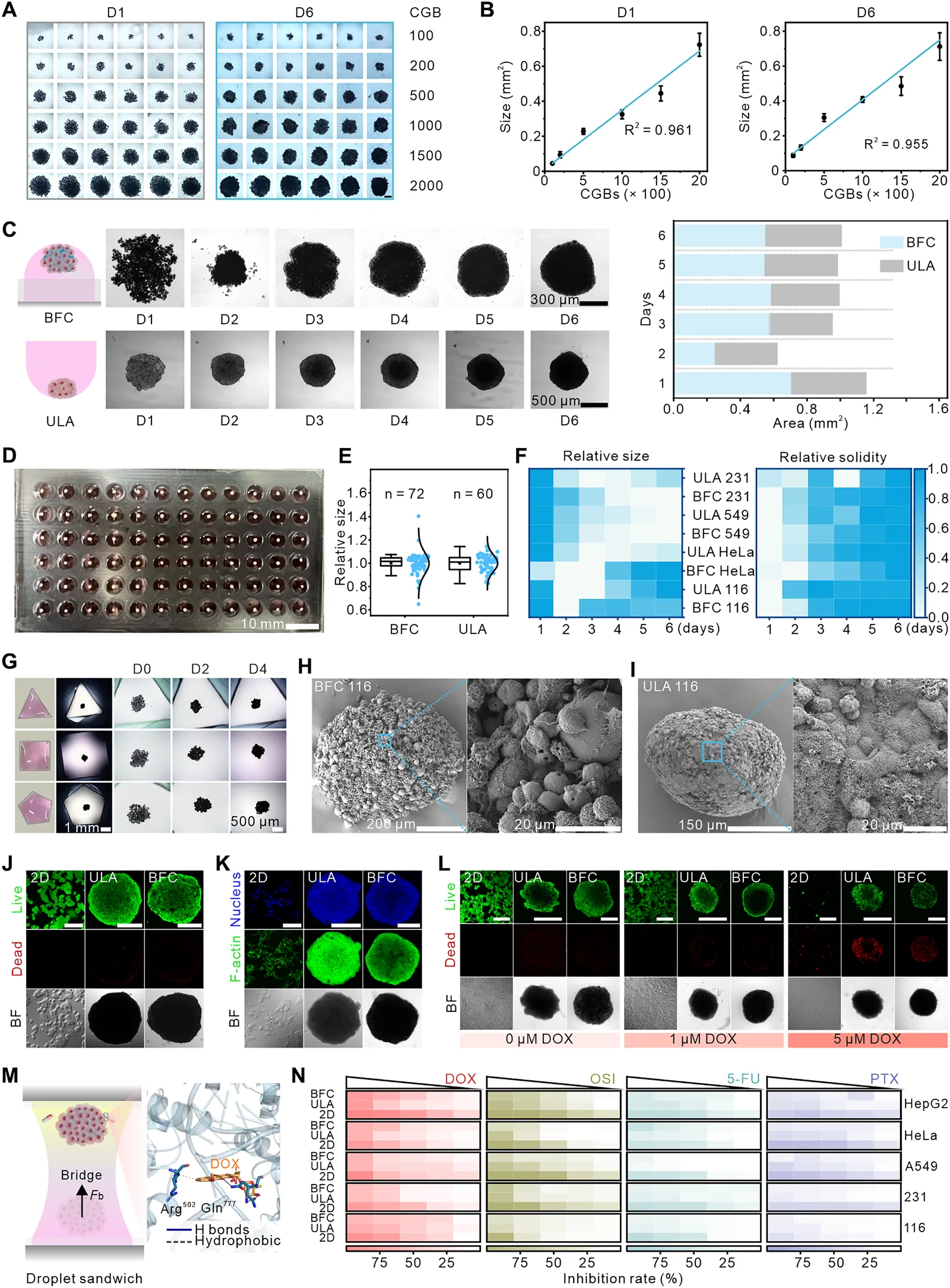
BFC-enabled reliable culture of cellular spheroids in parallel. (**A**) BFC spheroids formed with 100–2000 CGB after 1 day (D1) and 6 days (D6). Scale bar, 500 μm. (**B**) Linear relationships between CGB number and BFC spheroid size on D1 and D6; mean ± SD (n = 5). (**C**) Representative morphologies and size changes of HCT116 spheroids cultured from D1 to D6 using BFC or ULA. The D1–D6 images are intentionally repeated in fig. S22. (**D**) Photograph of HCT116 spheroids cultured in a 72-well droplet microarray via BFC. The average spheroid area was 1.13 ± 0.10 mm^2^ (mean ± SD; RSD, 9.2%). (**E**) Box plots and distribution profiles of relative spheroid size for BFC (n = 72) and ULA (n = 60). (**F**) Time-dependent changes in relative size and solidity of MDA-MB-231, A549, HeLa, and HCT116 spheroids cultured from D1 to D6 using BFC or ULA. (**G**) BFC spheroids cultured from D0 to D6 in triangular, square, and pentagonal microwells with 2.5-mm circumcircle radii. (**H** and **I**) Representative SEM and enlarged images of HCT116 cell-spheroids cultured using BFC (H) and ULA (I). (**J** and **K**) Live/dead staining (J) and immunostaining (K) of HCT116 cells cultured by the three methods for 6 days. BF, bright field. (**L**) Live/Dead staining of HCT116 cells cultured using the three methods after treatment with 0, 1, or 5 μM DOX. (**M**) Schematic of BFC spheroid transfer and drug efficacy evaluation with a scheme of DOX molecular docking. (**N**) Drug inhibition rates for HepG2, HeLa, A549, MDA-MB-231, and HCT116 cells cultured using 2D, ULA, or BFC and treated with different concentrations of DOX, OSI, 5-FU, or PTX. 231, 116 denote MDA-MB-231, and HCT116, respectively, in (F, H, I, and N). Scale bars in (J to L), 100 μm for 2D, 400 μm for ULA and BFC.

We further selected human colorectal cancer cells (HCT116) as a model for spheroid formation. Upon rapid attaching of HCT116 cells onto the CGB surface, the buoyant cell-CGB cluster spontaneously floated upward and congregated at the top of a droplet for growth via BFC (Fig. 3C). We note that BFC adapts the bubble buoyancy to inherent curvature and surface tension of droplets for guiding cells aggregation and facilitating their growth into spheroids (fig. S19). For example, during initial 3 days culture, the branched cell-CGB cluster (D1) sharply shrank into a cohesive spherical entity (D2), with a reduction in size by at least 2.8 folds, and then expanded to a typical cellular spheroid (D3) (Fig. 3C). This finding suggests a self-adaptive spatial organization and proliferation of cells in orchestrating with the free-aggregated bubbles. A similar morphological transition of cell-spheroid was also observed in conventional ULA culture, which harnessed anti-adhesive hemispherical substrates for cell aggregation (Fig. 3C), but showed much smaller change amplitude (< 20%) in the first 3 days culture. Besides that, we found that our BFC strategy was highly consistent with ULA in the pattern of spheroidal growth, during the entire culture period (6 days), suggesting the reliability of BFC in cellular spheroids culture.

By using a microarray chip (12 × 6 wells), we cultured 72 cellular spheroids simultaneously via a BFC method (Fig. 3D). This approach allowed forming consistent HCT116 cellular spheroids in size across the 72 entities (RSD = 9.2%), which was at the same level of the 60 cell spheroids after growing for 6 days using the ULA method (RSD = 6.9%) (figs. S20 and S21). In comparison, we note that BFC-based cell spheroids exhibited lower interquartile range values than those cultured in ULA (Fig. 3E), indicating more compact and uniform cell sizes with a narrower size distribution, and the superior consistency of BFC for cell spheroids culture. We then selected HeLa, human lung cancer cell (A549), and breast cancer cell (MDA-MB-231) as buoyant culture models to investigate the versatility of the BFC technology (figs. S22 to S25). By comparing the morphological indicators (size and solidity) of cell-spheroid formed between BFC and ULA across the four types of cells, we found that both culture methods showed a larger area at the initial cell aggregative state (D1), followed by a reduction in size as the cells migrated and cohered in a spatially adaptive fashion (D2), and then they entered proliferation and expansion (Fig. 3F). In addition, we observed that the solidity of cell-spheroid increased gradually when using both BFC and ULA methods, especially among the first 3 days, irrespective of cell types (Fig. 3F). This result indicates the thickness of the cell layers starting to increase with the formation of 3D structures. Throughout the entire cell growth process, the aspect ratio and roundness of the cellular spheroids tended toward 1.0 (fig. S26), suggesting the spheroidal shape being increasingly symmetrical and stable. We further investigated the effect of culture well geometries (e.g., triangular, square, and pentagonal wells) on cellular spheroidal morphology. As shown in Fig. 3G, we found cells cultured in these symmetrically shaped microwells showing consistent morphology evolution in self-buoyant spheroidal formation, also including that in original circle wells (2.5 mm diameter) and even the much larger, 7 mm diameter ones (fig. S27). This finding indicates that floating spheroidal organization is primarily governed by droplet surface morphology rather than culturing substrate topography, ensuring uniform spheroid formation regardless of underlying geometric variations.

We next used SEM to clarify the assembly architecture of single BFC spheroids (Fig. 3H), which revealed the cells dispersion among multiple CGB to form a 3D spheroidal cytoarchitecture through a combination of cell-cell adhesion and cell-CGB interactions. Figure 3I also showed a similar cell-spheroid morphology formed by the ULA method, but at a smaller size without typical bubble scaffolding structures. Although both methods demonstrated a favorable reproducibility in high-throughput cell-spheroid culture, the BFC showed following merits: (i) relying primarily on the number of CGB to tune cell spheroids varying in sizes, without requiring precise cell counting; (ii) not only mitigating the costs in 3D cell culture, but also facilitating the manipulation of both the culture medium and the resulting cell-spheroid, obviating the need of external field forces; and (iii) forming one-spheroid-per-well via self-levitated aggregation, instead of potential multi-spheroid-per-well in ULA or microwell culture (*38*).

### Reliable BFC cellular spheroids

Given the importance of cell-spheroid proteomics in revealing cellular states, functions and protein expression dynamics (*39*, *40*), we first conducted proteomics analysis of self-buoyant HCT116 cell-spheroids, and examined the differences between that derived by BFC and ULA, as well as traditional 2D culture (fig. S28). A Venn diagram showed that 96.60% of the proteins were shared among the 2D, ULA, and BFC methods, with uniquely identified proteins accounting for 0.44%, 0.32%, and 0.17% in each type. In comparison, 50 proteins were shared between the 2D and ULA methods, 92 proteins between BFC and ULA, whereas only 37 proteins were shared between 2D and BFC. This finding indicates a high similarity in protein expression profiles between the BFC and ULA cell-spheroids, with a notable difference from cells cultured in 2D. We next conducted gene set enrichment analysis (GSEA) to further explore the reliability of BFC spheroids by comparing different cell culture methods (fig. S28). Compared to cells cultured in 2D, the ULA counterparts showed positive enrichment in genes associated with hypoxia, coagulation, and epithelial mesenchymal transition (EMT), while exhibiting negative enrichment in cell cycle-related genes. Like ULA, we found that BFC spheroidal cells also showed significant positive enrichment in EMT genes, and additionally in metabolism-related genes including bile acid, fatty acid, and xenobiotic metabolism. Besides, we noticed a significant negative enrichment only for hypoxia and inflammatory response (*P* < 0.001), suggesting that BFC spheroidal cells did not exhibit a pronounced hypoxic response, but provided an aerobic environment conducive to cell growth. Overall, the GSEA and proteomic results indicate that self-buoyant BFC cellular spheroids show significant differences from those cultured in 2D, and are more similar to that by ULA method.

By using the droplet sandwich method, we next performed live/dead staining and immunostaining on BFC-based spheroids (fig. S29), and compared them with the cells cultured by 2D and ULA methods. We observed that cells cultured by all these methods exhibited high cellular viability (Fig. 3J and figs. S30 and S31). Especially, we found fewer dead cells in BFC spheroids than ULA-cultured ones, indicating that the BFC method could provide a more reliable and viable environment for cell-spheroid culture. Upon staining for F-actin, we observed that F-actin primarily localized at the cell membrane in 2D cells, whereas in cellular spheroids (by ULA and BFC), F-actin distributed both at the cell membrane and within the cytoplasm, signifying the formation of 3D cellular structures and high cellular viability (Fig. 3K and fig. S32). Moreover, Ki-67/F-actin staining confirmed proliferative and cytoskeletal organization in both ULA and BFC spheroids (fig. S33), whereas hypoxia staining showed that BFC spheroids displayed weaker hypoxic signals than ULA spheroids (fig. S34), consistent with enhanced oxygen transport through the CGB-supported architecture. Doxorubicin (DOX) penetration analysis showed more homogeneous early-stage drug distribution in BFC spheroids and comparable later-stage radial profiles between BFC and ULA spheroids (fig. S35). In addition, Yes-associated protein (YAP) staining revealed a local increase in nuclear YAP localization in BFC spheroids, suggesting that the retained rigid CGB scaffold may still introduce regional mechano-transduction cues (fig. S36). These results show BFC as a scaffold-assisted spheroid engineering platform that preserves 3D multicellular features while altering hypoxia, diffusion, and local cell-material interactions.

Given that the organ-like 3D cellular spheroids could more accurately reflect in vitro drug effects as compared to 2D cell models owing to the presence of drug concentration gradients (*38*, *41*), we further used the BFC sandwich method to test the potential of self-buoyant spheroids in drug evaluation. By taking a classic chemotherapeutic agent, DOX that binds to DNA topoisomerase II, as an example, droplet microarrays allowed rapid and high-throughput DOX administration and washout via droplet sandwiching (Fig. 3L and fig. S37). Upon exposure for 48 h, we compared the drug response of HCT116 cells and spheroids cultured using the 2D, ULA, and BFC methods. As shown in Fig. 3M, we found a clear decrease in the number of 2D-cultured cells with increasing DOX concentrations. In contrast, spheroids grown via ULA and BFC maintained relatively intact morphologies. At high DOX concentrations (5 μM), nearly all 2D cells exhibited signs of death. Meanwhile, ULA-cultured spheroids retained their morphology due to cell-to-cell encapsulation, whereas BFC spheroids underwent noticeable deformation because of loosened intercellular connections, allowing for a clear visualization of the cellular status. At moderate DOX concentrations (1 μM), both ULA and BFC-cultured spheroids remain intact, suggesting similar drug responses between the two types of spheroids.

To better demonstrate the reliability and advantage of BFC spheroids in drug evaluation, we used microarray droplet sandwich to deliver gradient concentrations of clinical anticancer pharmaceutics, such as DOX (from 5 to 0.01 μM), Osimertinib (OSI, from 20 to 0.1 μM), fluorouracil (5-FU, 250 to 50 μM), and paclitaxel (PTX, 100 to 1 nM). Following their 48-h exposure on cells or spheroids cultured using the 2D, ULA, and BFC methods, we evaluated the drug responses to various cell types across HepG2, HeLa, A549, MDA-MB-231 and HCT116. As shown in Fig. 3N, we found a heterogeneous drug-responsive pattern, due probably to the different cancer-cell killing mechanisms of drugs (e.g., DOX acting on topoisomerase II, OSI-specific binding to epidermal growth factor receptor (EGFR) receptors, 5-FU as an antimetabolite, and PTX targeting the microtubule system), and inconsistent drug penetration across 2D cells or 3D cellular spheroids. Inhibition-rate quantification (fig. S38) and IC_50_ analysis (fig. S39) showed that 2D cells were generally more sensitive to drug treatment, whereas 3D spheroids displayed higher drug tolerance under many conditions. Notably, BFC spheroids exhibited ULA-like responses in some cases, but also showed 2D-like or strong drug sensitivity in others, such as DOX treatment in HeLa cells or OSI treatment in A549 cells. This may be ascribed to the CGB-supported internal architecture, which can reduce compact cell packing, improve mass transport, weaken hypoxia-related stress responses, and alter early drug penetration (fig. S35). Overall, these findings indicate that BFC spheroids exhibit scaffold-assisted drug-response characteristics distinct from diffusion-limited avascular tumor spheroids and are suitable for high-viability spheroid culture, rapid medium/drug exchange, drug-response monitoring, and spheroids-based co-culture analysis (fig. S40).

### Programmable multi-spheroid horizontal assembly

We next implemented BFC droplet sandwich to program the bioassembly of multiple cellular spheroids for assembloids, thereby enhancing our understanding of tissue formation (*22*, *24*). Unlike coalescence of lateral droplets (*5*), our BFC approach synergized droplet surface tension (*F*_st_) and inherent spheroidal buoyancy (*F*_b_) to direct the spontaneous accumulation (or transfer) of cellular spheroids at (or from) the apex of the droplet. This autonomous feature facilitated the programmable assembly of multiple spheroids. As shown in Fig. 4A, when a droplet with cell-spheroid 1 was inverted, the spheroid thus self-moved upward to the droplet bottom. By kissing with another upright droplet, the floated spheroid 2 flashily transferred to the inverted droplet via the dominant buoyancy. After separating the up-down droplets, the up droplet was inverted again, thus allowing the two cellular spheroids to collide and merge spontaneously at the top of the droplet. Likewise, three and more spheroids could be self-assembled in a programmable fashion.

**Fig. 4.**
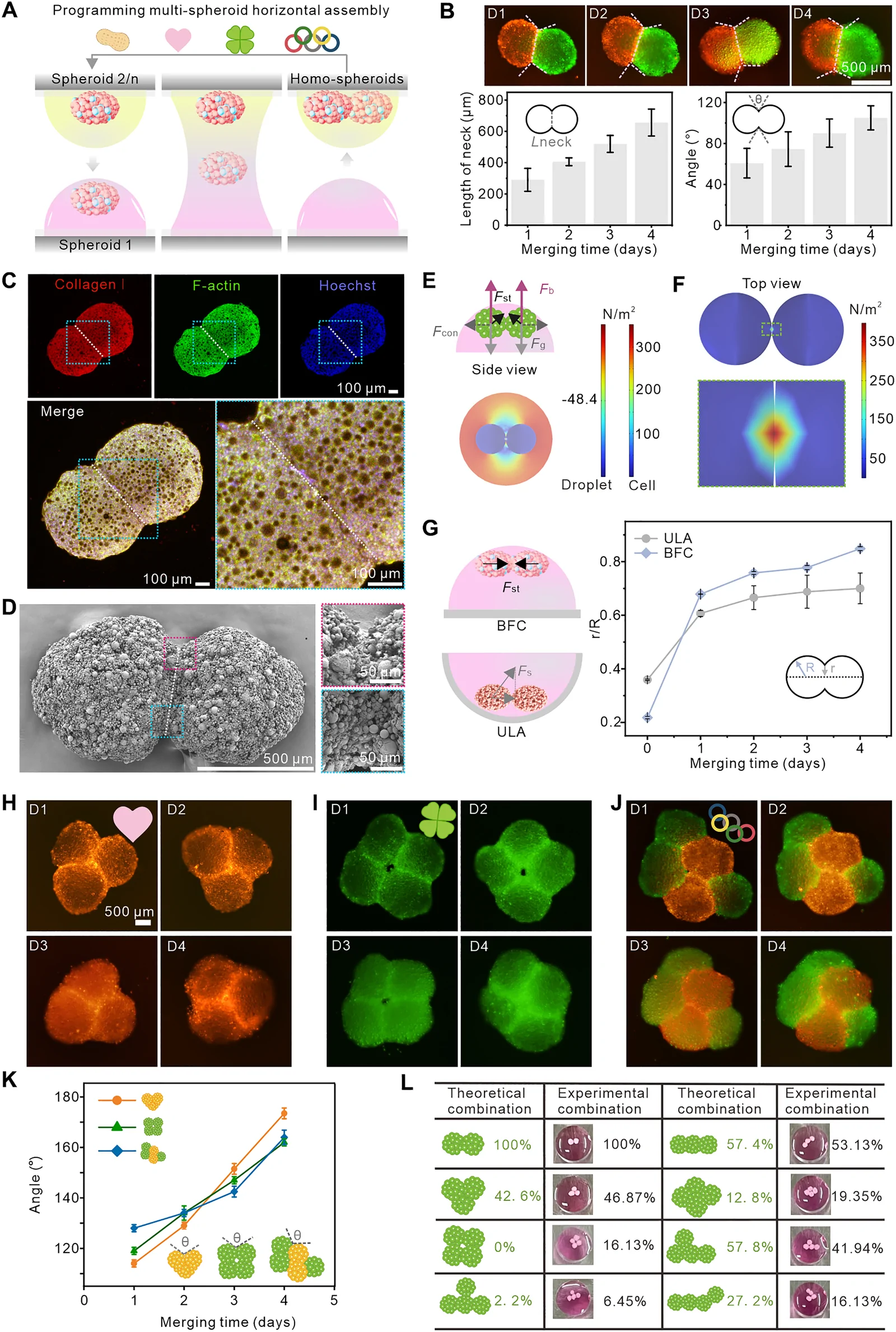
Programming horizontal bioassembly of multi-homo-spheroids via BFC. (**A**) Schematic of sequential droplet kissing used to assemble spheroids into peanut, heart-shaped, four-leaf-clover, and Olympic-ring patterns. (**B**) Fluorescence images and time-dependent changes in *L*_neck_ and interspheroid fusion angle (θ) between the two cell-spheroids as they merged from D1 to D4. Gray dotted line denotes the *L*_neck_ and fusion angle; mean ± SD (n = 5). (**C**) Collagen I, F-actin, and nuclear staining of peanut-shaped dual spheroids. The enlargement showed the fusion region (blue dashed box), and the fusion neck (white dashed line). (**D**) SEM images of the peanut-shaped dual-spheroids. The zoom-in SEM images showed the fusion region (purple and blue dashed box), and the fusion neck (white dashed line). (**E**) Simulation of the force distribution between two cellular spheroids at the apex of a droplet. (**F**) Top-view stress distribution and enlargement of the contact region. (**G**) Comparison of neck fusion evolution (r/R) between BFC and ULA methods as two cell-spheroids merged from D1 to D4. *F*_st_ denotes droplet surface tension; *F*_s_ denotes supporting force from ULA external wall; mean ± SD (n = 3). (H-J) Fluorescent microscopic images of the BFC-programmed multi-spheroid bioassembly patterns across tri-spheroid sweet heart (**H**), tetra-spheroid four-leaf clover (**I**) and penta-spheroid Olympic rings (**J**), and their fusion over time from D1 to D4. (**K**) Time-dependent shifting of fusion angles in (H) to (J) during spheroidal merging from D1 to D4; mean ± SD (n = 3). (**L**) Schematic diagrams, probabilities, and corresponding examples of diverse HCT116 multi-spheroids between theoretical and experimental combinations. In the theoretical model, cell spheroids are approximated as spheres, with combinatorial probabilities calculated through simulation algorithms (fig. S44), and the experimental combinatorial probability derived from experimental statistical data (n = 36).

Figure 4B shows the BFC-tuned assembly and time-dependent fusion between two HCT116 cellular spheroids. During the ‘peanut’-shaped dual-spheroid fusion from D1 to D4, we observed the dynamic change of two key indicators, fusion “neck” length (*L*_neck_) and fusion angle (θ) between the two spheroids. By digitally recording the fusion boundary length, we found that the *L*_neck_ extended from ∼300 to 700 μm over 4 days growth and the fusion angle increased from ∼60° to 100°. To clarify the fusion neck at a cellular level, we stained the dual-spheroid with Hoechst (for nucleus), Collagen I (for extracellular matrix) and phalloidin (for F-actin) (Fig. 4C). An enlarged fluorescent image allowed clearly visualizing the tight junction structure between the two HCT116 spheroids. An SEM image of the dual-spheroid also confirmed the initial formation of a peanut-like fusion complex and the intimate interactions between cells at the interface of individual spheroids (Fig. 4D). The zoom-in image further stressed the bubble-scaffolded cellular linkage in the inter-spheroid boundary. This finding indicates that the BFC-driven self-assembly not only brings the spheroids together, but also promotes spheroids fusion via cellular migration and merging. To enhance our comprehension of the internal and external determinants influencing 3D cell fusion, we used finite element simulation to investigate the force distribution between two cellular spheroids self-floating at the apex of a droplet (Fig. 4E). Due to the synergic effect between surface tension (*F*_st_) of the droplet and the spheroidal *F*_b_, overcoming the spheroidal contact force (*F*_con_) and gravity (*F*_g_), the two cell-spheroid squeezed each other from the central of their physical contact (i.e., the strongest stressing point) (Fig. 4F), thereby facilitating subsequent fusion. As shown in Fig. 4G, BFC demonstrated markedly faster fusion speed and higher neck fusion evolution rate (r/R) as compared to gravity-based collision using ULA plates (fig. S41). This is probably attributed to the synergistic promotion effect of spheroidal buoyancy and droplet surface tension with capillary action in BFC (*42*), whereas ULA relies solely on horizontally oriented forces (*F*_s_) generated by support from the external wall. Consequently, buoyancy-activated cell spheroids enable rapid, programmable self-assembly without external intervention.

Beyond dual-spheroid assembly, we further used sequential BFC droplet sandwich to program the horizontal assembly of multi-spheroid. As shown in Fig. 4, H to J, we observed a three spheroids-encoded ‘sweet heart’ buoyantoid with each other at approximately 120° (Fig. 4H), a four spheroids-encoded ‘four-leaf clover’ architecture with each aligned at ∼90° (Fig. 4I), and a five spheroids-encoded ‘Olympic rings’ pattern with a relatively symmetrical structure (Fig. 4J). These findings suggest that the BFC method facilitates the controlled and programmable assembly of buoyantoid into specific geometric configurations. During the culture period (from D1 to D4), we also found a typical time-dependent buoyantoid fusion with increasingly cohesive architectures (Fig. 4, H to J), and larger fusion angles (Fig. 4K), as an enlarged merging spheroid. Immunostaining and SEM of multi-spheroids also revealed tight connections between the neighboring spheroids (figs. S42 and S43). Figure 4L shows the theoretical and experimental probability in assembling two, three, and four BFC spheroids in a 2D plane via the sandwich method. Using a tension rotation angle-based algorithmic model (fig. S44), we achieved an excellent theoretical and experimental fitting. By adjusting the size of merged cell spheroids (fig. S45), the volume of culture droplets (fig. S46), and the topography of culture wells (fig. S47), the assembly with different spheroid ratios could be achieved. These results suggest the controllability of BFC sandwich method in programmable bioassembly of various cell spheroids.

### Programmable multi-spheroid vertical assembly

In addition to programmable assembly of multi-spheroid in the 2D plane, we also achieved bioprinting-free vertical programming of cell spheroids assembly by leveraging capillary force (*F*_c_) and spheroidal buoyancy (*F*_b_) in BFC without the need of external fields. This self-powered strategy expanded spatial encoding of spheroid fusion from horizontal to vertical dimension, enabling the top-down linear assembly of spheroids varying in colors, numbers, and sizes, which are typically difficult to achieve on planar culture substrates or via non-bioprinting methods. As shown in Fig. 5A, when a capillary tube (1 mm inner diameter) contacting the droplet apex, the buoyant cell spheroid was swiftly drawn into the tube for moving up driven by the Laplace pressure generated at the droplet-air interface (*29*) and inherent buoyancy of BFC spheroid. As the spheroid continued to ascend, the surface tension (*F*_st_) at the in-tube liquid surface could trap it within the solution for suspension culture (*17*). To transfer spheroids simultaneously in parallel, we engineered a ‘spheroidal pipette’ by fixing multiple capillary tubes (e.g., six-channel) onto a 3D-printed array positioner (Fig. 5B). By moving this spheroidal pipette along a BFC droplet microarray, the buoyant cell spheroids spontaneously entered the tubes in parallel for programmable arrangement of multi-spheroid in sequence (Fig. 5C), thus allowing the bioprinting-like vertical assembly of spheroidal strings (Fig. 5D).

**Fig. 5.**
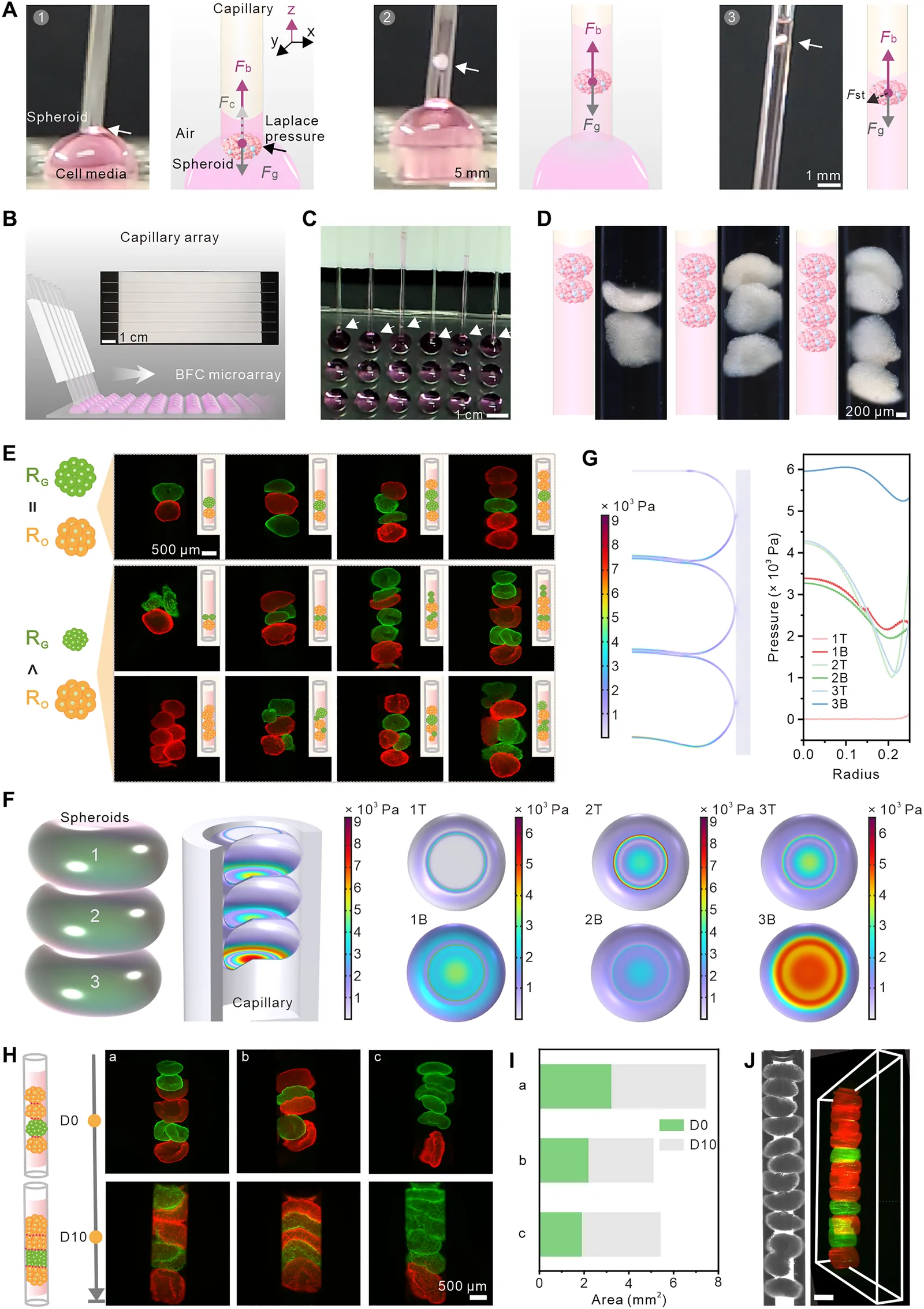
Programmable vertical assembly of multi-spheroids. (**A**) Images and schematics of BFC-driven cellular spheroid transfer and vertical assembly in a capillary tube. 1) Capillary tube contacting a BFC spheroid levitating at a droplet apex for transferring via buoyancy (*F*_b_), capillary force (*F*_c_) and Laplace pressure; 2) *F*_b_-dominated cell spheroid moving upward within the capillary tube; and 3) Cell spheroid levitating at the top of liquid surface in the capillary tube. (**B**) Array assembly of spheroidal strings by moving capillary array (six-channel) fixed on a 3D-printed positioner (Inset) along a BFC droplet microarray. (**C**) A photo showing multiple cell spheroids simultaneously entering a capillary array. The spheroids are highlighted with white arrows. (**D**) Microscopic images of vertical assembly of two, three, and four cell spheroids in tubes. (**E**) Fluorescence images of vertically assembled spheroidal strings encoding in capillary tubes, featuring both same-sized and differently-sized cell spheroids (R_G_ = R_O_ and R_G_ < R_O_), labeled by DiI (Cy3 channel) and DiO (green channel). (**F**) Finite element simulation of pressure distribution on three cell spheroids with vertical floating and up-down contacting in a capillary tube. T indicates spheroidal top; B indicates spheroidal bottom. (**G**) Forcing distribution across different positions of individual spheroids. (**H**) Fusion of differently encoded spheroidal strings after 10-day culture following programmable vertical assembly of cell spheroids in capillaries. (**I**) Size statistics of three types of spheroidal strings at 0 day (D0) and D10 in (H). (**J**) Bright-field (left) and 3D reconstructed fluorescent images (right) of an encoded spheroidal string vertically assembled by 12 cell spheroids arranging within a capillary. Scale bar, 500 μm.

We next separately used two lipophilic fluorescent dyes, such as DiI (orange-yellow, O) and DiO (green, G), to label HCT116 cellular spheroids for encoding string assembly. By controlling the relative sizes between spheroids varying in colors (O and G), such as R_G_ = R_O_ (radius, R) and R_G_ < R_O_ (or R_G_ > R_O_), we constructed spheroidal strings with different encoding patterns, and found close contact existing between upper and lower spheroids either in R_G_ = R_O_ or R_G_ < R_O_ vertical assembly (Fig. 5E), thereby benefiting spheroidal fusion. We next used finite element simulation to analyze the forcing distribution across three top-bottom (T-B) contacting cellular spheroids with same sizes. As shown in Fig. 5F, prominent compressive forces were observed at the spheroid-spheroid contact regions, primarily concentrating at the central contact area and gradually decreasing toward the periphery (Fig. 5G). Especially, the bottom spheroid, which is submerged in the aqueous phase, experiences the highest pressure (∼5.5 × 10^3^ Pa) beneath it, primarily due to hydrostatic pressure. At the upper contact interfaces, such as 1B-2 T and 2B-3 T, compressive forces between each pair of spheroids gradually reduced from bottom to up (Fig. 5, F and G). This longitudinal mechanical compression may facilitate the self-assembly of spheroidal string and promote their fusion.

Furthermore, three differently encoded spheroidal string-like assembloids were cultured for 10 days within the capillary tube. As shown in Fig. 5H, we found the boundaries between the up-down spheroids progressing from close contact to full fusion. In addition, the size of each spheroid string increased over time, at least by ∼50% (Fig. 5I), and fully occupied the tube laterally after 10-day culture. Using this power-free bioprinting-like platform, we successfully engineered tissue-like strings with over 12 encoding spheroidal units (Fig. 5J). Theoretically, the number of encoding units per string depends on the number of spheroids and capillary tube length without the need of external fields, thus overcoming the constraint of traditional 3D bioprinting (e.g., demanding external fields and potential spheroidal lesion) and providing a foundation for assembling ultra-long tissue-like assembloids. These results indicate that our BFC droplet approach has great potential for engineering complex multi-spheroid-based tissue-like architectures via tempo-spatially controlled organization, deepening the understanding in tissue engineering.

### Encoding hetero-spheroid assembly for fusion application

In addition to homo-spheroid assembly and fusion, we next examined the coded hetero-spheroid fusion processes (Fig. 6A), and their promotion and inhibition by regulating molecules. Indeed, in multicellular organisms, diverse cell types arrange spatially in tissues, and collaborate each other to execute particular functions, such as tumor invasion (*17*). To realize such heterogeneous multi-spheroids, we cultured two distinct breast cell types (tumor MDA-MB-231 and normal MCF-10A) into spheroids using the BFC method, and then transferred pairs of spheroids (DiI-stained MDA-MB-231 spheroid and DiO-stained MCF-10A spheroid) into the same droplet for self-buoyant assembly and fusion. As shown in Fig. 6B, we found geometrically distinct hetero-spheroids coded by two, three, or even seven MDA-MB-231 and MCF-10A spheroids in different combinations, including peanut-shaped dual-spheroids, string bead-like tri-spheroids, sweet heart-shaped tri-spheroids, and flower-shaped hepta-spheroids. We also found that distinct boundaries between the two cell types within the hetero-spheroids remained evident at 3 days post-fusion, although progressive fusion was observed (Fig. 6B and fig. S48). This finding suggests that the BFC method enables programmable assembly and fusion of hetero-spheroids through spheroid-level spatial encoding, driven by the synergistic effect of droplet surface tension and buoyancy-activated self-aggregation. By assembling hetero-dual-spheroids either with the same size (RMCF-7 = RMCF-10A) or varying in cell surface tension (σMCF-7 < σMDA-MB-231) (Fig. 6C), we examined their time-dependent fusion in 3 days culture. Figure 6D shows the dominant invasion of tumor MCF-7 and MDA-MB-231 spheroids with higher σ relative to their normal and low σ counterparts, respectively. This feature made the BFC method an ideal platform to study tumor invasion within the 3D tissue microenvironment.

**Fig. 6.**
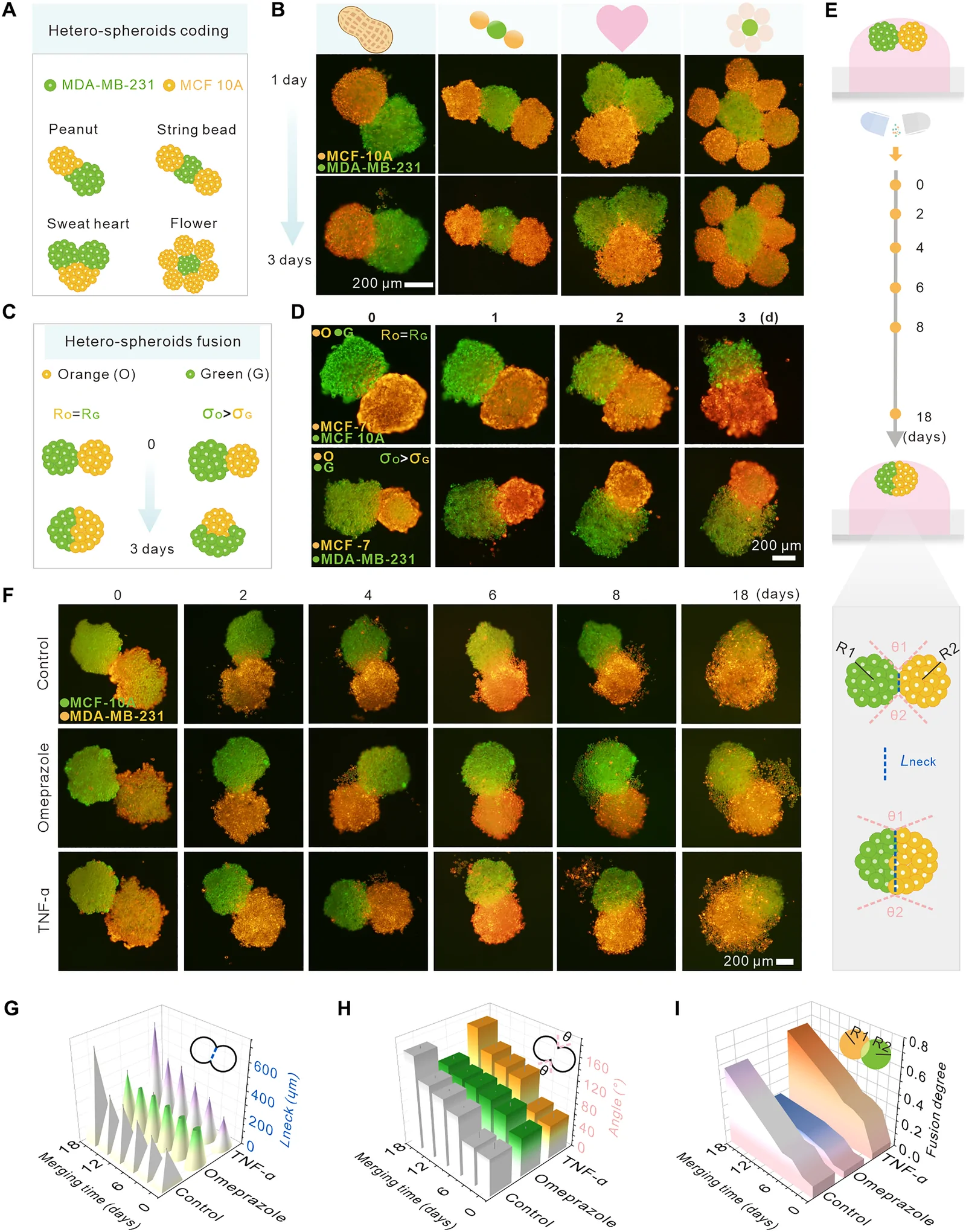
Encoding hetero-spheroids bioassembly and fusion. (**A**) Schematic of hetero-spheroids coding. Using different cell spheroids (green, MDA-MB-231; orange, MCF-10A) to encode four different cell-spheroid patterns, in sequence: di-spheroid peanut, tri-spheroid string bead, tri-spheroid sweet heart and hepta-spheroid flower topography. (**B**) Programmable hetero-spheroids assembly (top) and fusion for 3 days (down) between MCF-10A and MDA-MB-231 spheroids with four topographical patterns in (A). (**C**) Schematic of hetero-spheroids fusion between them either with the same size (R_O_ = R_G_) or varying in surface tension (σ_O_ > σ_G_). (**D**) Time-dependent fusion between MCF-7/MCF-10A and MDA-MB-231/MCF-7 hetero-spheroids in 3 days culture. (**E**) Schematic of self-buoyant hetero-dual-spheroids fusion model for dynamic assessing of tumor invasion regulated by biochemical factors, such as pro-metastatic factor (TNF-α) and anti-metastatic compound (Omeprazole). (**F**) Topographic images of MCF-10A/MDA-MB-231 hetero-dual-spheroids at each time point (0, 2, 4, 6, 8 and 18 days) during the fusion process by exposing to varied biochemical environments. Blue and pink dashed lines denote neck length (*L*_neck_) and tangent angle (θ), respectively, of the merged spheroids. (**G** to **I**) Comparison of the *L*_neck_ (G), angle (H) and fusion degree (I) of the merged hetero-dual-spheroid between the TNF-α (10 ng/mL), Omeprazole (200 μM) and control group. R1 and R2 denote each spheroidal radius of two hetero-spheroids. Error bars represent as mean ± SD (n ≥ 3).

We next investigated the fusion process between hetero-spheroids, akin to the metastatic migration of 3D microtumors. Given that specific chemicals can effectively hinder or promote this 3D fusion and metastasis (*5*), we explored the invasion response of paired hetero-spheroids when subjected to specific external stimuli (Fig. 6E), such as the tumor necrosis factor-α (TNF-α, 10 ng/mL) and the anti-metastatic compound (Omeprazole, 200 μM). As shown in Fig. 6F, we observed a preferentially wrapped and time-dependent fusion process between MDA-MB-231 tumoroids and normal MCF-10A spheroids in both TNF-α group and the control, resulting in a Janus hybrid-spheroid, whereas almost no such a process in the omeprazole group. To quantitatively depict the fusion process, we measured the neck length (Lneck, blue dashed line as shown in Fig. 6E) and the degree of tangent angle (pink dashed line) between the merged region (Fig. 6F). In control group, the initial Lneck between the two hetero-spheroids was about 248.50 ± 6.25 μm, and gradually increased to 666.44 ± 14.93 μm after merging for 18 days, while in the antagonist omeprazole group, the Lneck increased only to 132.59 ± 13.78 μm for the same merging time (Fig. 6G), probably due to inhibition of omeprazole on the expression of pro-metastatic genes, such as matrix metalloproteinase-9 (MMP-9) and C-X-C chemokine receptor 4 (CXCR4). By contrast, the Lneck increased by 440.76 ± 3.24 μm in TNF-α group, much higher than other two groups, suggesting an enhanced invasion process promoted by the pro-metastatic factor of TNF-α. Beyond Lneck, we also found the different tangent angles among the three groups. For example, the angle enlarged from 70.34° ± 3.82° to 126.28° ± 4.60° in omeprazole group (Fig. 6H), with a fusion degree of 19.2% (Fig. 6I and fig. S49), while the two hetero-spheroids almost completely fused (near 180°) in the other two groups (Fig. 6H), showing the fusion degrees reached 56.8% and 65.3% in the control group and the promotion group, respectively (Fig. 6I). These findings suggest that the BFC method can not only encode living materials with controllable composition and topology, but also provide a promising tool to decode the metastatic phenotypes between different tumoroids, and to screen the corresponding bioactive molecule candidates.

## DISCUSSION

In this work, we show a self-powered buoyantoid culture technology (i.e., BFC) with microbubbles to streamline one-spheroid-per-well array culture and programmable multi-spheroid bioassembly for assembloid engineering. This approach leverages self-buoyant cellular spheroids and droplet surface tension to program homo- and hetero-assembloids with symmetric geometry patterns across peanut, sweet heart, four-leaf clover, Olympic rings and flower-like topographies. BFC also enables bioprinting-free vertical assembly of cell-spheroids in parallel. Such attractive multi-spheroid patterns are difficult to achieve by other spheroidal assembly strategies, such as manual manipulation and 3D bioprinting (*43*), while obviating the need for external-field actuation. In BFC, the buoyancy-activated self-aggregation of bubble-cell clusters renders the cell-spheroid growth self-adaptive in droplets through a one-step adsorption of cells onto PLL-grafted microbubbles, benefiting one-drop-one-spheroid. By engineering a droplet sandwich microarray system, a facile top-down droplet kiss allows ultrafast transfer of self-buoyant cellular spheroids between adjacent droplets in a single step, thereby enabling efficient spheroid relocation, media exchange, and drug delivery. More importantly, such a multifunctional spheroid manipulation system allows scalable and programmable multi-spheroid assembly and fusion in a horizontal or vertical fashion, advancing the study in tumor invasion and drug efficacy evaluation.

It should be noted that BFC spheroids are scaffold-assisted hybrid spheroids rather than conventional scaffold-free spheroids. The retained CGBs provide buoyant support and cell-adhesive interfaces that enable self-floating spheroid formation, rapid transfer, medium/drug exchange, and programmable multi-spheroid assembly, without direct exposure to air due primarily to droplet surface tension and hydrophilicity of BFC spheroids. The PLL-coated CGBs and attachment solution used here represent a practical condition for the tested cell lines, whereas other cell types may require further optimization due to their varied surface charge and adhesion properties. Meanwhile, the CGB scaffold introduces non-native cell–material interactions and may alter local mechanics, cell packing, diffusion geometry, hypoxia development, drug penetration, and long-term tissue maturation compared with ULA or hanging-drop spheroids. During prolonged culture, increased tissue mass and effective density may cause a subset of BFC spheroids to sink; however, almost no sinking was observed within the first week experimental window used for staining, drug treatment, transfer, and multi-spheroid assembly. Therefore, BFC spheroids should not be considered as a universal replacement for scaffold-free spheroids or native tumor tissues, especially for applications requiring faithful modeling of tissue compression, avascular tumor hypoxia, necrotic-core formation, spontaneous cell sorting, or in vivo-like tissue mechanics. Rather, BFC is best positioned as a self-buoyant spheroid-engineering and assembloid-construction platform that is particularly useful when high-throughput spheroid manipulation, repeated solution exchange, high viability, and spatially programmable multi-spheroid assembly are required.

As summarized in table S1, compared with ULA and hanging-drop methods, BFC enables rapid parallel spheroid transfer and solution exchange (∼1–10 s), accommodates larger droplets for handling and assembling larger spheroids, and supports self-driven, controllable horizontal and vertical assembly while retaining external-field-free single-spheroid culture and reusable non-contact array operation. Overall, our BFC technology offers some merits over prior scaffold-based 3D cell culture (*14*), including (i) self-buoyant high-throughput cultivation without requiring any external fields, such as magnetic and acoustic fields (*19*, *44*, *45*), thus preventing the endocytosis of magnetic beads and potential cellular damages (*17*); (ii) improved mass transport and viability in the tested scaffold-assisted spheroids, which may be advantageous for applications prioritizing long-term viability and solution exchange (*46*); (iii) the inherent buoyancy and self-aggregation of CGB allowing for single-spheroid-per-well culture with ultrafast spheroid transfer (∼1 s), facile medium exchange and no cross-contamination; (iv) such a self-floating culture mode renders the BFC array chip substrate contactless with the spheroid in growth, and thereby makes it reusable to largely reduce the cost of 3D cell culture in a high-throughput manner; and (v) arrayed spheroids culture and assembly with consistent focal planes facilitating the downstream scalable screening analysis. Especially, programmable assembly and promoted fusion of buoyantoids underscore the power of BFC technology in bioprinting-free engineering of large multi-layer tissue-like assembloids, such as centimeter-scale tissues with fewer cells (*47*). By combining the physical self-aggregation of CGBs with the biological self-organization of cells, this buoyancy-driven platform enables the parallel construction of spatially organized heterotypic assembloids, including tumor–stroma, tumor–immune, and tumor–vascular co-culture models, for investigating inter-spheroid cross-talk, cell migration, invasion, fusion, tissue reorganization, and therapeutic responses in tissue engineering, disease modeling, regenerative medicine, and drug discovery (*12*, *35*, *46*, *48*–*50*).

## MATERIALS AND METHODS

### Materials

GB (iM30K, density 0.6 × 10^3^ kg/m^3^) were purchased from 3M Company (USA). PLL, Hoechst 33258, PBS, and sucrose were obtained from Shanghai Sangon Biotech Co. Ltd. (China). Fluorescein isothiocyanate (FITC)-phalloidin was purchased from ABclonal Biotеchnоlоgy Co. Ltd. (China). Cell Counting Kit-3D, Calcein-AM/PI Cell Viability/Cytotoxicity Assay Kit, Cell Plasma Membrane Staining Kit with DiO/DiI were purchased from Beyotime Biotеchnоlоgy Ltd. (China). AZDye 488 NHS Ester was purchased from Vector Laboratories, Inc. (USA). BSA, Doxorubicin (DOX), Osimertinib (OSI), 5-Fluorouracil (5-FU) and Paclitaxel (PTX) were obtained from Solarbio Biotech Co. Ltd. (China). PDMS (Sylgard 184) consisted of a base and a curing agent was purchased from Dow Corning Corporation (USA). Hexamethyldisilazane (HMDS) was purchased from Aladdin Chemistry Co., Ltd. (China). Sealing membrane was purchased from Bemis Parafilm (USA). Capillary tubes were obtained from Nanjing PBO Quartz Technology Co., Ltd. (China).

### Cell culture

All cells were purchased from Procell Life Science & Technology Co., Ltd. (China). Human HCT116, HepG2, HeLa, A549 and MDA-MB-231 cells were cultured in high-glucose DMEM supplemented with 10% FBS and 1% penicillin-streptomycin. Human MCF-10A cells were maintained in the MCF-10A cell-specific complete growth medium recommended by the supplier. All cells were cultured at 37°C in a humidified incubator with 5% CO_2_ and were passaged at approximately 80% confluency.

### Preparation and characterization of CGB

1 g of GB was suspended in 5 mL of 0.1 mg/mL PLL solution and incubated at room temperature for 10 min. The mixture was then centrifuged at 150g for 1 min, after which the solution was removed using a syringe and the pellet was resuspended in ultrapure water. This washing process was repeated three times. Finally, the resulting pellet was vacuum-dried to obtain GB@PLL (i.e., CGB).

The morphology of GB and CGB were observed using field emission scanning electron microscope (FESEM, Zeiss, Sigma 300, Germany) and high-resolution transmission electron microscope (HRTEM, JEOL-F200, Japan).

Surface chemical composition of GB and CGB was determined by an XPS (Thermo Scientific K-Alpha, USA). GB and CGB were vacuum-dried overnight before being transferred into the system for analysis. Survey spectra over a binding energy range of 0 to 1000 were measured for GB and CGB. High-resolution XPS spectra of N 1 s, C 1 s, and O 1 s were also collected. Binding energies were calibrated by C-C peak of adventitious carbon (C 1 s = 284.6 eV).

### Engineering bubble droplet microarray

PDMS (PDMS: curing agent Sylgard 184 = 10:1) was poured into a copper mold, degassed under vacuum, and cured at 80°C for 2 h. The PDMS was then peeled off the mold and cut into an appropriate size. Both the PDMS and the coverslip underwent plasma treatment (1 min, PDC-32G, Harrick, USA). Following the treatment, the PDMS was bonded with the coverslip and placed at 80°C for 2 h to obtain the droplet microarray.

### 3D cell culture using ULA and BFC

For ULA culture, cells were diluted in the culture medium to a concentration of 5 × 10^4^ cells/mL. Subsequently, 100 μL of the cell suspension was added to each well of the ULA plate and incubated in a cell culture incubator.

For BFC culture, 1 mg of CGB (approximately 10^5^ CGBs) was suspended in 50 μL of attachment solution (0.5 × PBS + 200 mM sucrose). A 50 μL aliquot of cell suspension containing approximately 5 × 10^5^ cells was then added, and the mixture was vortexed at 1000 rpm for 1 min to promote cell-CGB attachment. Immediately after the attachment step, complete culture medium was added to dilute the attachment solution and adjust the CGB concentration to 10^4^/mL. A 10 μL aliquot of the cell-laden CGB suspension was transferred into each microarray well containing 45 μL of complete culture medium. The floating cell-CGB clusters were cultured in the droplets for 24 h and subsequently transferred to fresh complete culture medium using the sandwich method (i.e., droplet kiss) for further incubation.

Then the 3D cultured spheroidal area, aspect ratio, roundness, and solidity were measured and calculated by ImageJ. The shapes of spheroids were outlined by “freehand selections” or “analyze particles”.

### SEM characterization of cell spheroids

The cell spheroids were transferred into 1.5 mL centrifuge tubes and gently washed once with PBS. Excess PBS was aspirated, and the spheroids were then fixed with 2.5% glutaraldehyde in PBS at room temperature for 20 min. Following fixation, the samples were washed again with PBS. Dehydration was achieved through a graded ethanol series (30%, 50%, 70%, 85%, 95%, and 100%) with each step lasting for 10 min. Subsequently, the cell spheroids were treated with 50% HMDS in ethanol and then with 100% HMDS for 10 min each to facilitate further drying. After removing the HMDS solutions, the samples were left to air-dry at room temperature. Once dried, the samples were sputter-coated with gold to enhance conductivity and mounted on the SEM sample stage for observation.

### Proteomics

Proteomic testing and analysis were conducted by Majorbio Biopharm Technology Co., Ltd. (China). Proteins were extracted from lysed cells, then enzymatically digested, and analyzed using the Astral-DIA method. Bioinformatic analysis of the resulting data was performed on the Majorbio Cloud platform (https://cloud.majorbio.com). GSEA was carried out using the Hallmarks dataset with default parameters.

### Cell viability staining

Live/dead staining was performed according to the manufacturer’s instructions. The spheroids were incubated with Calcein-AM (1:1000 dilution) and PI (1:1000 dilution) at 37°C for 30 min. After incubation, the spheroids were washed with PBS and imaged using a confocal laser scanning microscope (CLSM, Olympus, FV3000, Japan).

### Immunostaining

After washing the cell spheroids with PBS, they were fixed with 4% paraformaldehyde at room temperature for 10 min. Following fixation, the spheroids were washed once with PBS and permeabilized with 0.1% Triton X-100 at room temperature for 20 min. After another wash with PBS, the spheroids were incubated with FITC-labeled phalloidin and Alexa Fluor 594-labeled Collagen I in a solution containing 1% BSA and 0.1% Triton X-100, protected from light at room temperature for 1 h. Upon completion of the incubation, the spheroids were washed twice with PBS and stained with Hoechst 33258 at 37°C for 20 min. After staining, the spheroids were washed once more with PBS. The stained cell spheroids were then imaged using CLSM.

### Evaluation of drug activity

After cell digestion, 5,000 cells were seeded separately into 2D 96-well plates, ULA plates, and BFC as mentioned before for cultivation. After 2 days culture, drugs were added to achieve the following final concentrations: DOX at 5, 1, 0.5, 0.1, and 0.01 μM; OSI at 20, 10, 5, 1, and 0.1 μM; 5-FU at 250, 200, 150, 100, and 50 μM; and PTX at 100, 50, 10, 5, and 1 nM. Their culture processes were continued for an additional 2 days. For cells cultured in 2D conditions, cytotoxicity was measured using the CCK-8 reagent. For cells cultured in ULA and BFC conditions, cytotoxicity was evaluated using the CCK-3D reagent. The inhibition rate of drugs on cells was calculated according to formula

$$\text{Inhibition Rate}\left(\%\right)=\left(1-\frac{{\mathrm{OD}}_{\text{treated}}}{{\mathrm{OD}}_{\text{control}}}\right)\times 100\%$$ (1)

### Spheroidal fusion

Following culture using the BFC method, individual or array spheroids were selected for fusion. The fusion process involved sandwiching two spheroids within a single droplet to promote their fusion. The fused spheroids were then further cultured for growth (e.g., 4 days). Throughout this period, the culture medium was refreshed every second day to maintain optimal growth conditions. After the extended culturing period, the fused spheroids were stained for imaging or fixed for SEM according to the previously described protocols.

### Programmable bioassembly of cell spheroids

Horizontal assembly: HCT116 cells were cultured using the BFC method, followed by staining of the spheroids with DiO or DiI. Multiple droplets containing spheroids were then gathered into a single droplet using the sandwich method and cultured further. The spheroid assemblies were observed using an upright fluorescence microscope (Olympus). The cell culture medium was gently replaced by the “kiss method” as needed. The spheroid neck and contact angle were quantified using ImageJ software.

Vertical assembly: HCT116 cell spheroids were cultivated using the BFC method. Subsequently, the spheroids were stained with DiO and DiI. The spheroids were then aspirated into capillaries (diameter 1 mm, length 12 cm) inclined at approximately 80° via capillary action for assembly. One end of each capillary was sealed with a sealing membrane, and the capillaries were vertically placed in an incubator (37°C, 5% CO_2_) to facilitate initial contact between spheroids via buoyant forces. For the assembly of arrays in the vertical direction, a 3D-printed mold was used to fix the capillaries into an array formation, and cell spheroids were sequentially aspirated according to the aforementioned method. The interactions of the spheroids were monitored periodically over a 10-day period using an inverted fluorescence microscope (Zeiss). When necessary, the culture medium was gently replaced by aspirating from the side of the capillary. The total surface area was quantified using ImageJ software.

### Hetero-spheroids fusion and invasion assay

DiI- and DiO-stained MDA-MB-231 and MCF-10A BFC spheroids were placed in a droplet microarray device in pairs with 55 μL DMEM complete medium per well (control group), and 200 μM omeprazole (Shanghai Aladdin Biochemical Technology Co., Ltd.) and 10 ng/mL TNF-α (MedChemexpress Biotechnology company, USA) were added to the medium in the drug treatment group, respectively. Spheroidal buoyancy and the surface tension of the droplets promoted the contact between the initial spheroid. After that, the interaction of the spheroids was regularly monitored for 18 days using an upright fluorescence microscope. The cell culture medium was gently replaced by the “kiss method” as needed. The spheroid neck, contact angle, and radius were quantified using ImageJ software.

### Statistical analysis

Data are presented as mean ± standard deviation (SD). Unless otherwise specified, n represents technical replicates, including individual spheroids, droplets, wells, droplet-pair transfer assays, image fields, or spheroid assemblies analyzed under the same experimental condition. For drug-response assays, biological replicates were performed as independent experiments on different days using separately prepared cell cultures or spheroid batches. The sample size (n) and statistical test used for each analysis are specified in the corresponding figure legends. Figures and heat maps were generated using Origin 2021 and GraphPad Prism 9. Statistical analyses were performed using Origin 2021 and GraphPad Prism 9. For comparisons between two groups, two-tailed Student’s t-test was used. For comparisons among multiple groups, one-way ANOVA followed by the indicated multiple-comparisons test was used. For experiments involving two independent variables, two-way ANOVA followed by the indicated post hoc test was used. For time-dependent repeated measurements, repeated-measures ANOVA with Geisser–Greenhouse correction followed by the indicated multiple-comparisons test was used where appropriate. Differences were considered statistically significant when *P* ≤ 0.05. n.s., *P* > 0.05; *P ≤ 0.05; **P ≤ 0.01; ***P ≤ 0.001; ****P ≤ 0.0001.

### Ethics statements

The collection and use of human peripheral blood samples were approved by the Medical Ethics Committee of the First Affiliated Hospital of Guangxi Medical University (approval no. 2026-E0593). Peripheral venous blood samples (5 mL per donor) were obtained from five healthy adult volunteers after written informed consent had been obtained from each participant. All samples and experimental data were coded to protect participant privacy.

## Supplementary Materials

### The PDF file includes:

Supplementary Text Figs. S1 to S49 Table S1 Legends for movies S1 and S2 References
