# Supplementary material for: Self-buoyant cell-spheroid culture programming multi-spheroid assembly
Source: Sci Adv. 2026 Oct 7;12(41):eaee3306. doi: 10.1126/sciadv.aee3306 (PMC13644768; doi:10.1126/sciadv.aee3306)
Supplement: Supplementary file 1 [file sciadv.aee3306_sm.pdf]

Science Advances
AAAS

Supplementary Materials for

Self-buoyant cell-spheroid culture programming multi-spheroid assembly

Yuanhang Xiang *et al.*

Corresponding author: Fan Yang, yangfan@gxmu.edu.cn

Sci. Adv. **12**, eaee3306 (2026)
DOI: 10.1126/sciadv.aee3306

The PDF file includes:

Supplementary Text
Figs. S1 to S49
Table S1
Legends for movies S1 and S2
References

Other Supplementary Material for this manuscript includes the following:

Movies S1 and S2

Supplementary Text

Zeta potential measurement

To measure the zeta potential of GB and CGB, the microbubbles were ground in a mortar with a small amount of ultrapure water. The resulting suspension was transferred to a centrifuge tube and centrifuged at 1500 × g for 10 seconds to remove the intact floating bubbles from the top layer. The pellet was resuspended in ultrapure water and this washing step was repeated three times. 10 mg of the crushed bubbles were then reacted with 1 mL of 0.1 mg/mL PLL solution for 10 minutes. After the reaction, the mixture was centrifuged at 1500 × g for 10 seconds, and the supernatant was removed. The pellet was washed three more times with ultrapure water. Finally, 1 mg of PLL-modified bubble fragments were suspended in the measurement solution for zeta potential analysis.

To measure the zeta potential of cells, cells were harvested and washed twice with PBS. The cells were then suspended in the measurement solution. Approximately 5×10^3 cells were used for the zeta potential measurement.

Influence of different solutions on cell adhesion efficiency

HeLa cells were digested following the standard protocol and subsequently stained with DiO according to the manufacturer's instructions. Following staining, 10^5 CGB and 5×10^5 cells were incubated for 1 minute at room temperature in PBS, 0.5× PBS with 200 mM sucrose, 1% BSA in PBS, DMEM, and DMEM supplemented with 10% FBS, respectively. Solutions without added CGB served as controls. After mixing, 10 µL of the cells were transferred to a 96-well plate containing 100 µL of PBS, followed by imaging and cell counting. The number of unadsorbed cells (m) was compared against the total number of cells without added CGB (n). Adsorption efficiency was calculated as

$$\text{Adhesion efficiency (\%)} = \frac{n - m}{n} \times 100\% \quad (1)$$

For the cell viability assay following incubation with the solutions, 5,000 cells were seeded into each well of a 96-well plate. Once the cells adhered to the plate surface, the original culture medium was aspirated out, and the cells were washed with 100 µL of PBS. After removing the PBS, 100 µL of DMEM, PBS, or the attachment solution was added to each well. Following 1 minute incubation at room temperature, the solutions were aspirated, and the cells were stained with calcein-AM/PI according to the manufacturer's instructions and observed under a fluorescence microscope. Cell viability was calculated as

$$\text{Cell viability (\%)} = \frac{N_{live}}{N_{live} + N_{dead}} \times 100\% \quad (2)$$

Here, N_{live} represents the number of cells stained with calcein-AM, and N_{dead} represents the number of cells stained with PI.

To directly assess cell viability after the initial 24 h BFC culture, floating CGB-attached cell clusters were collected after culture in droplets containing complete culture medium for 24 h. The clusters were stained with calcein-AM and PI according to the manufacturer’s instructions.

To assess the effect of solution incubation on cell proliferation capacity, after incubation of the cells with different solutions, 100 µL of DMEM + 10% FBS was added back to the cells. The cells were then further incubated in an incubator for two days, and cell viability was measured using a CCK-8 assay kit according to the manufacturer's instructions.

Cell detachment assay during the initial BFC culture period

To evaluate cell detachment during the initial 24-h BFC culture period, cell-CGB clusters were prepared using the standard BFC protocol and sandwich transferred into fresh culture wells after the initial cell-CGB attachment step to remove initially unbound cells. At the indicated time points, cells accumulated at the bottom of the wells were stained with Calcein-AM and PI according to the manufacturer's instructions and imaged using a fluorescence microscope. The detached live- and dead-cell fractions were quantified based on the numbers of Calcein-AM-positive and PI-positive cells, respectively.

To estimate the number of cells initially attached to the CGBs, a defined fraction of the cell-CGB suspension was collected after the initial attachment step and imaged using a fluorescence microscope. The number of cells attached to the sampled CGBs was counted, and the total number of initially attached cells was estimated according to the proportion of sampled CGBs relative to the total number of CGBs used in each well.

To estimate the number of detached cells, Calcein-AM/PI staining was performed at the indicated time points. Representative images of the bottom of each well were acquired using the same imaging settings. The numbers of detached live cells and detached dead cells were counted in the sampled fields and extrapolated according to the ratio of the total well-bottom area to the imaged area.

The estimated detached live-cell fraction and detached dead-cell fraction were calculated as follows:

$$\text{Estimated detached live} - \text{cell fraction } (\%) = \frac{\mathrm{N}_{detached\ live\ cells}}{\mathrm{N}_{initially\ attached\ cells}} \times 100\% \quad (3)$$

$$\text{Estimated detached dead} - \text{cell fraction } (\%) = \frac{\mathrm{N}_{detached\ dead\ cells}}{\mathrm{N}_{initially\ attached\ cells}} \times 100\% \quad (4)$$

where $N_{\text{detached live cells}}$ and $N_{\text{detached dead cells}}$ represent the estimated numbers of detached Calcein-AM-positive and PI-positive cells, respectively, and $N_{\text{initially attached cells}}$ represents the estimated number of cells initially attached to the CGBs.

To evaluate the effect of detached cells on spheroid formation, the 24 h transfer step was omitted, and the cell-CGB clusters were continuously cultured in the presence of detached cells. Bright-field images were acquired at the indicated time points to monitor spheroid formation.

Theoretical calculations

When a CGB with a diameter of d_{CGB} μm carries n cells each with a diameter of d_{Cell} μm, it can just maintain suspension in the solution, implying:

$$F_{CGB} + n \times F_{Cells} \geq G_{CGB} + n \times G_{Cells} \quad (5)$$

where F_{CGB} is the buoyant force acting on one CGB, F_{Cells} is the buoyant force acting on one cell, G_{CGB} is the gravitational force acting on one CGB, and G_{Cells} is the gravitational force acting on one cell. The density of the bubble is 0.6×10^3 kg/m^3, and the density of the cells is approximately 1.08×10^3 kg/m^3.

The buoyancy acting on one bubble is:

$$F_{CGB} = \rho_{water} g V_{CGB} = \rho_{water} g \times \frac{\pi}{6}(d_{CGB} \times 10^{-6})^3 \quad (6)$$

The buoyancy acting on one cell is:

$$F_{Cells} = \rho_{water} g V_{Cell} = \rho_{water} g \times \frac{\pi}{6}(d_{cell} \times 10^{-6})^3 \quad (7)$$

The gravitational force acting on one bubble is:

$$G_{CGB} = m_{CGB}g = V_{CGB}\rho_{CGB}g = \frac{\pi}{6}(d_{CGB} \times 10^{-6})^3 \times \rho_{CGB} \times g \quad (8)$$

The gravitational force acting on one cell is:

$$G_{Cells} = m_{Cells}g = V_{Cells}\rho_{Cells}g = \frac{\pi}{6}(d_{cell} \times 10^{-6})^3 \times \rho_{Cells} \times g \quad (9)$$

From these calculations, it follows that:

$$n \leq \frac{(\rho_{water} - \rho_{CGB}) \times {d_{CGB}}^3}{(\rho_{cell} - \rho_{water}) \times {d_{Cell}}^3} \quad (10)$$

That is:

$$n \leq \frac{5 \times {d_{CGB}}^3}{{d_{Cell}}^3} \quad (11)$$

Molecular docking

The crystallographic structure of the complex Top II/DNA/etoposide (Protein Data Bank (PDB) ID: 3QX3) was fetched from the PDB (http://www.pdb.org). The AutodockTools was employed for manual preparation of the protein by adding non-polar hydrogen and Gasteiger charges and saved as pdbqt format. Openbabel 2.4.1 was used to generate a three-dimensional structure from the two-dimensional structure by genetic algorithm and MMFF94 force field. Hydrogen was added at pH 7.4 and saved as pdbqt format. Docking analysis was performed using AutoDock Vina. Search grid was identified as center_x: 33.026, center_y: 95.765, and center_z: 51.567 with dimensions size_x: 21.519, size_y: 21.519, and size_z: 21.519. The default search parameter values were used. The lowest binding energy pose obtained in the docking was selected and interaction analysis was carried out using PyMOL.

Computer simulation

Simulations were performed using COMSOL Multiphysics 6.0 (COMSOL, Massachusetts, USA). The hemispherical droplet has a diameter of 5 mm and a height of 1 mm. The cell spheroids are oblate spheroidal with an equatorial radius of 0.25 mm and a polar diameter of 0.05 mm. The spheroids aggregate at the top of the droplet surface or stack inside the capillary containing the liquid. The dynamic viscosity of the cells is 7.7×10^{-4} Pa • s, the density of the cells is 800 kg/m^3, the elastic modulus of the cell membrane is 5000 Pa, the Poisson's ratio of the cell membrane is 0.35, and the density of water is 1000 kg/m^3. The fluid domain surrounding the water is defined as a moving mesh region; the bottom surface is set as an open boundary condition, and the cell material is modeled as linearly elastic.

HCT116 spheroid–PBMC co-culture assay

HCT116 spheroids were prepared using the standard ULA and BFC spheroid culture procedures. Human peripheral blood mononuclear cells (PBMCs) were isolated from peripheral blood samples obtained from healthy volunteers using a human PBMC isolation solution kit (Solarbio, China) according to the manufacturer's instructions. After isolation, PBMCs were resuspended in culture medium and gently added to the spheroid cultures at approximately 1×10^4 PBMCs per spheroid. For the co-culture groups, PBMCs were added to ULA or BFC HCT116 spheroids, while spheroids cultured without PBMCs were used as corresponding controls. Bright-field images were acquired on days 2, 4, and 6 to monitor spheroid morphology and PBMC-induced

spheroid disruption. On day 6, spheroids were stained using Calcein-AM and propidium iodide (PI) to assess live and dead cells, respectively, followed by fluorescence imaging.

Ki67 and F-actin staining of ULA and BFC spheroids

ULA and BFC spheroids were collected and processed according to the immunostaining procedure described in the main Methods section. Briefly, spheroids were fixed, permeabilized, and blocked, followed by simultaneous incubation with an anti-Ki67 primary antibody (Thermo Fisher Scientific, MA5-15690) and FITC-labeled phalloidin for F-actin staining. After washing, the samples were incubated with a Cy3-conjugated secondary antibody to visualize Ki67. After secondary antibody incubation and washing, nuclei were stained with DAPI before fluorescence imaging.

DOX penetration assay

ULA and BFC spheroids were prepared according to the standard spheroid culture procedures described above. For drug penetration analysis, spheroids were incubated with doxorubicin (DOX) at a final concentration of 5 μM. Fluorescence images were acquired at 0.5, 1, 2, 4, 24, and 72 h after DOX addition to monitor time-dependent drug penetration. The average DOX fluorescence intensity of each spheroid was quantified using ImageJ. Radial penetration profiles were generated by measuring DOX fluorescence intensity from the spheroid periphery toward the center at selected time points.

YAP and F-actin staining of ULA and BFC spheroids

ULA and BFC spheroids were collected and processed according to the immunostaining procedure described in the main Methods section. Briefly, spheroids were fixed, permeabilized, and blocked, followed by simultaneous incubation with an anti-YAP primary antibody (Cell Signaling Technology, D8H1X, 14074) and FITC-labeled phalloidin for F-actin staining. After washing, the samples were incubated with a Cy5-conjugated secondary antibody to visualize YAP. After secondary antibody incubation and washing, nuclei were stained with DAPI before fluorescence imaging.

Heterogeneous sphere coding

After MDA-MB-231 and MCF-10A cells were cultured into spheroids by the BFC method for 6 days, the MDA-MB-231 and MCF-10A cell spheres were stained with DiI and DiO (Beyotime Biotechnology Co., Ltd., Shanghai, China), respectively. Different combinations of DiI and DiO-stained MDA-MB-231 and MCF-10A spheroids were transferred to a new droplet microarray device as shown in the schematic diagram in Figure 6a and assembled. Afterward, spheroids interactions was monitored at regular intervals for 3 days with an upright fluorescence microscope (Olympus).

Fusion degree analysis

Fluorescence images were used to depict morphological changes during the fusion process. The degree of fusion (FD) of the spheroid pair was calculated using the following formula:

$$FD = 1 - \frac{S}{R_1 + R_2} = \frac{R_1 + R_2 - S}{R_1 + R_2} \tag{12}$$

In this context, FD indicates the fusion degree of the spheroid pair, S denotes the distance from the tumor spheroid's center to the normal spheroid's center, R_1 signifies the tumor spheroid's radius, and R_2 represents the radius of the normal spheroid (see **Fig. S49**).

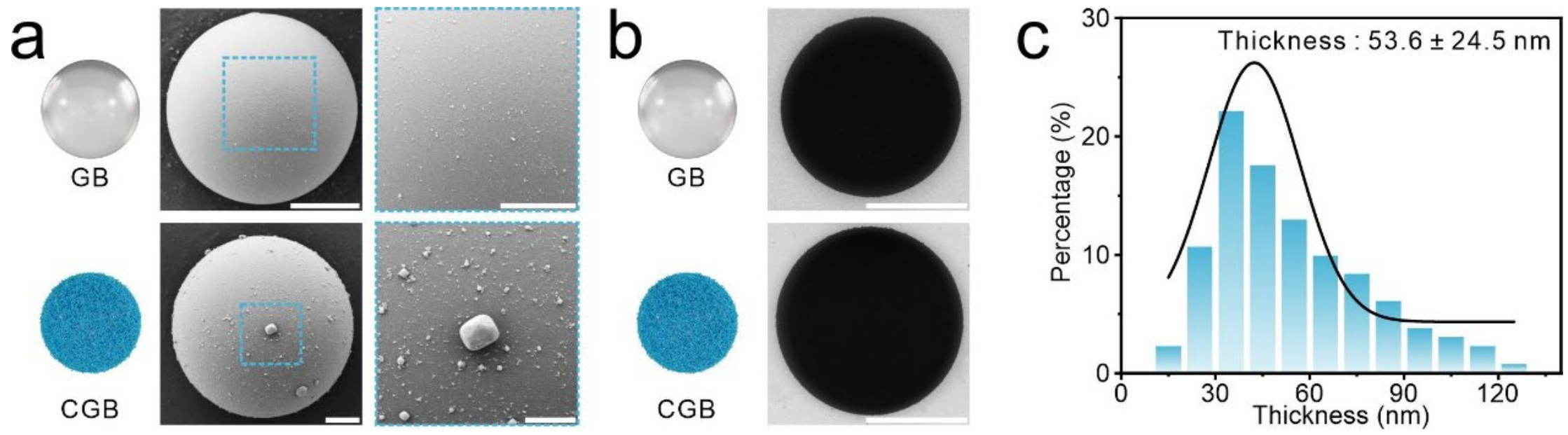

Fig. S1. Surface morphology characterization of GB and CGB. (a) Scanning electron microscopy (SEM) images of GB and CGB. Scale bars: 10 µm (left), 5 µm (right, enlarged view). (b) Transmission electron microscopy (TEM) images of GB and CGB. Scale bars: 10 µm. (c) Measured PLL thickness distributions on CGB. Thickness is expressed as mean ± standard deviation (SD).

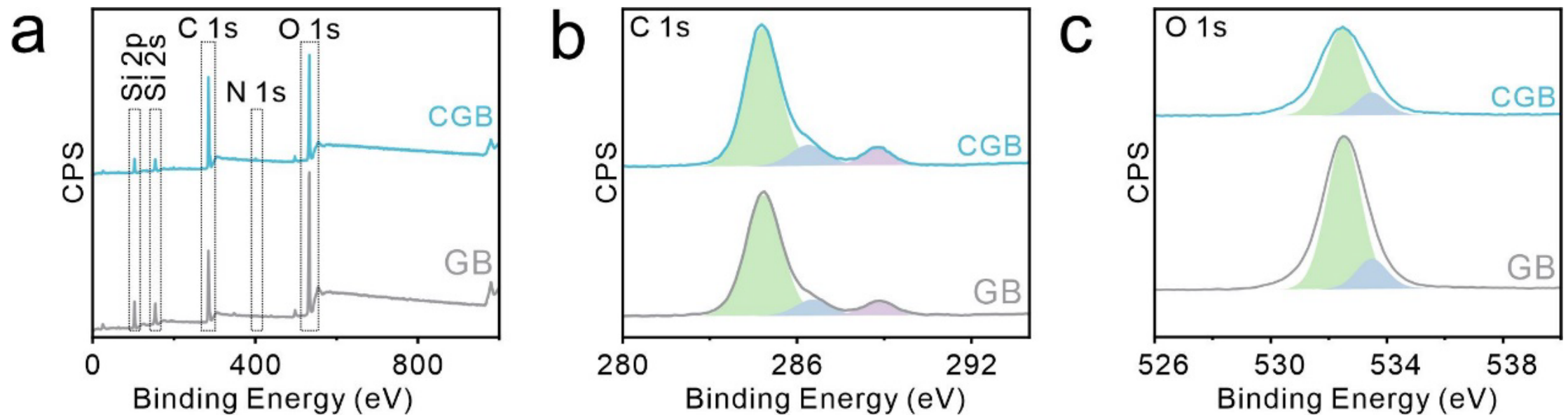

Fig. S2. Surface elemental characterization of GB and CGB. (a) X-ray photoelectron spectroscopy (XPS) survey spectrum of GB and CGB. (b) High-resolution XPS spectrum of carbon (C 1s) for GB and CGB. (c) High-resolution XPS spectrum of oxygen (O 1s) for GB and CGB.

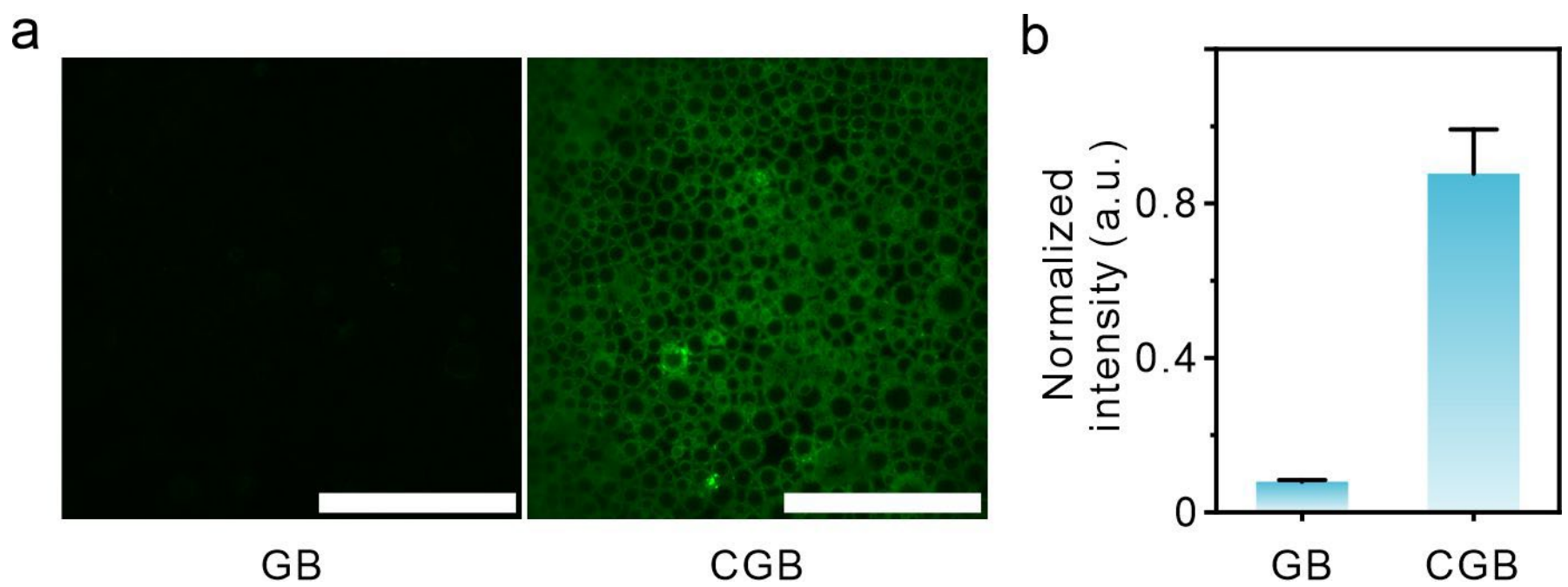

Fig. S3. Fluorescence characterization of CGB. (a) Fluorescence imaging of GB and CGB in reactions with AZDye 488 NHS ester. Scale bars: 300 µm. (b) Surface-normalized fluorescence intensities of AZDye 488-labeled GB and CGB. Error bar means ± SD (n = 5)

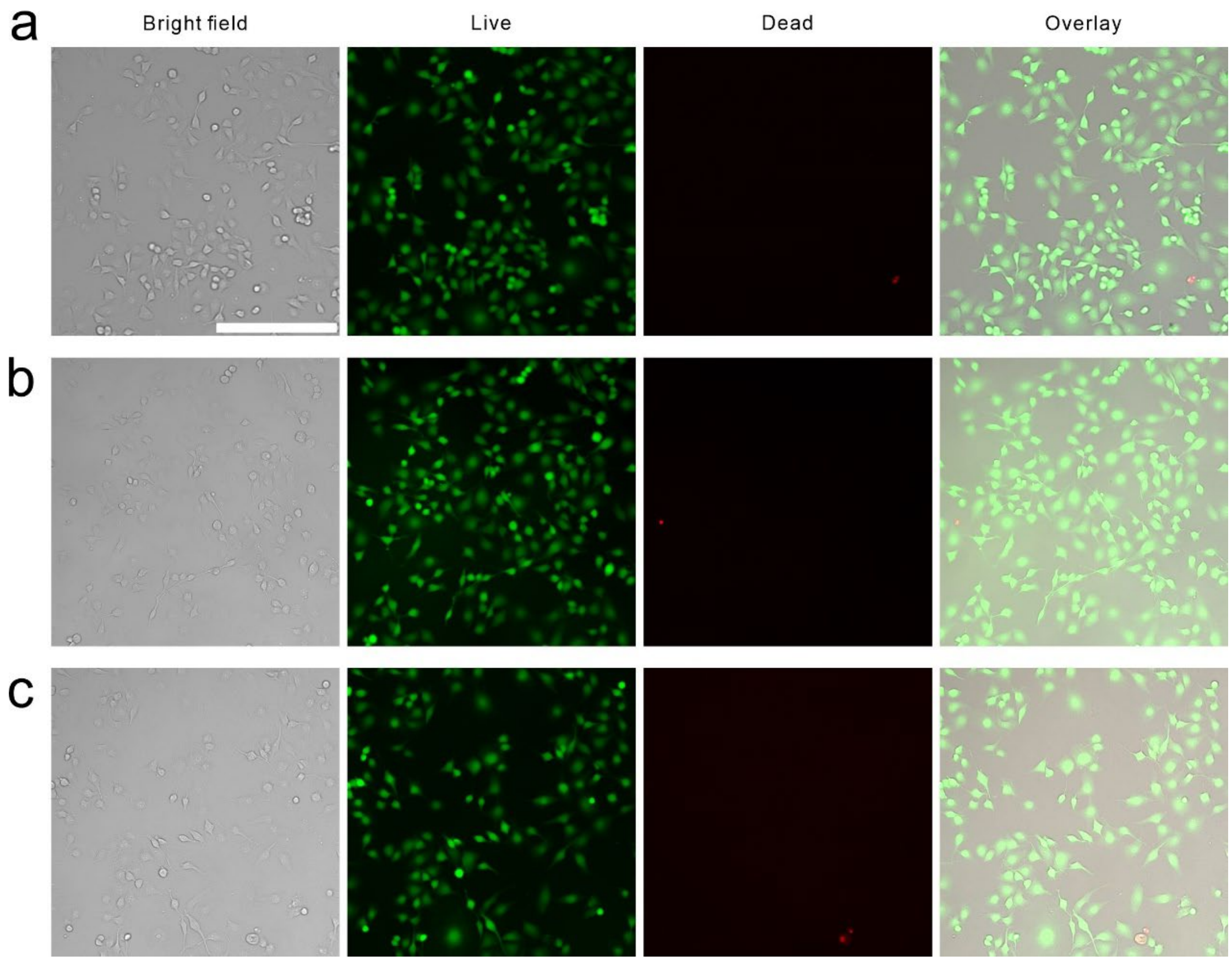

Fig. S4. Short-term cytocompatibility of the solutions used during cell-CGB attachment. Live/Dead staining of HeLa cells after 1 minute exposure to DMEM (a), PBS (b), or the attachment solution (c). Live cells were stained with calcein-AM (green), and dead cells were stained with PI (red). Scale bar: 400 μm.

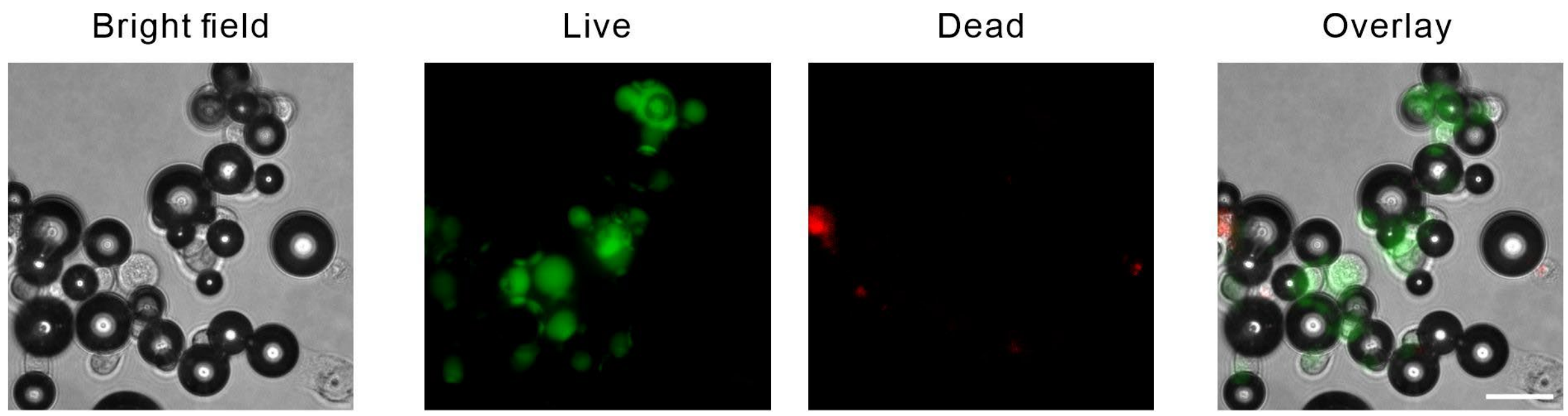

Fig. S5. Live/Dead staining of floating cell-CGB clusters after the initial 24-h BFC culture. Representative fluorescence images showing cell viability after 24 h of culture under the BFC condition. Live cells were stained with calcein-AM (green), and dead cells were stained with PI (red). Scale bar: 50 μm.

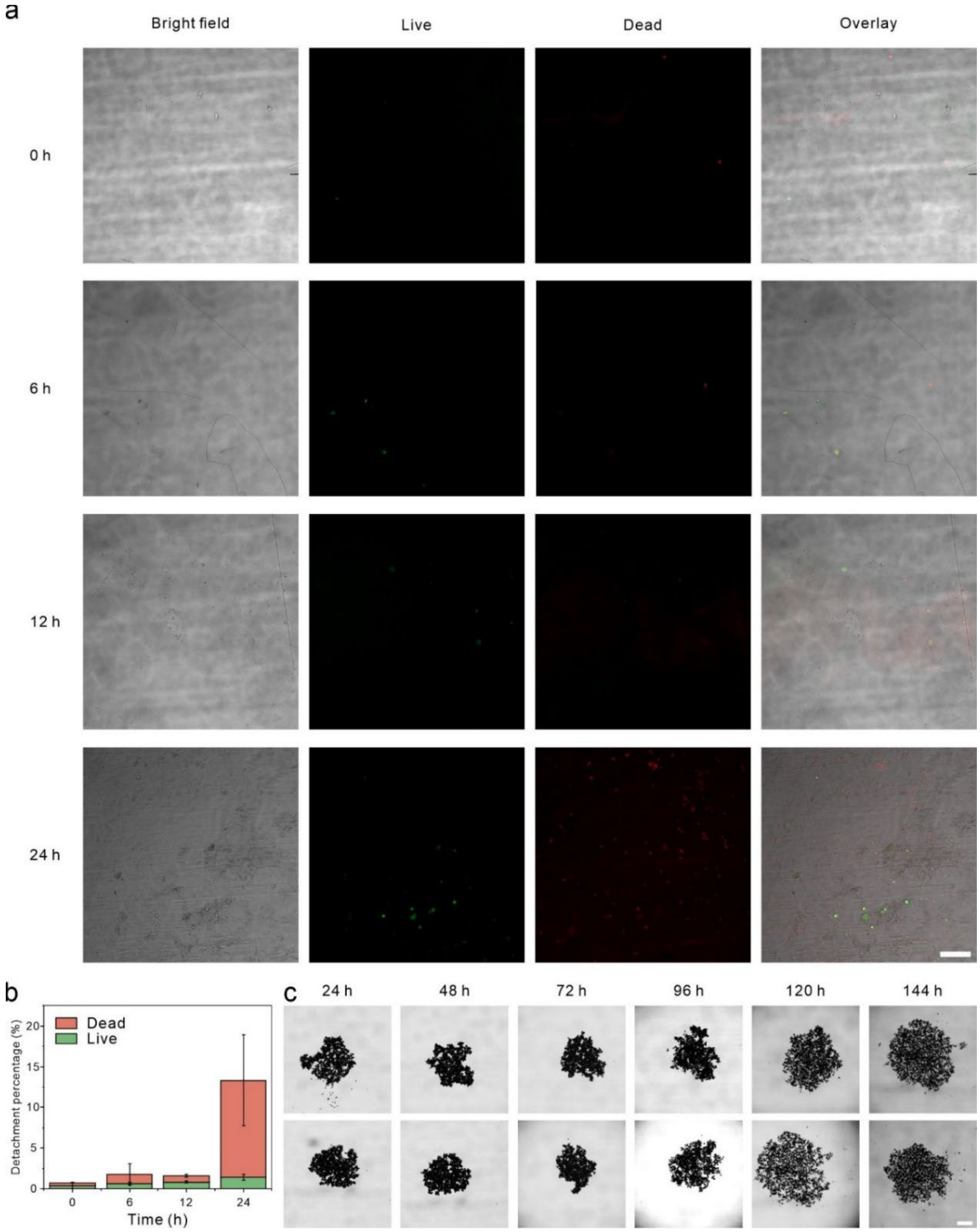

Fig. S6. Detachment of dead cells during the initial BFC culture period and its effect on spheroid formation. (a) Representative Calcein-AM/PI staining images of cells detached from the CGBs and accumulated at the bottom of the wells at the indicated time points. Live cells were stained with Calcein-AM (green), and dead cells were stained with PI (red). Scale bars: 100 µm. (b) Quantification of the detached live- and dead-cell fractions at the indicated time points. Data are presented as mean ± SD (n = 3). (c) Representative bright-field images of cell-CGB clusters cultured without the 24 h transfer step from 24 to 144 h. Scale bars: 200 µm.

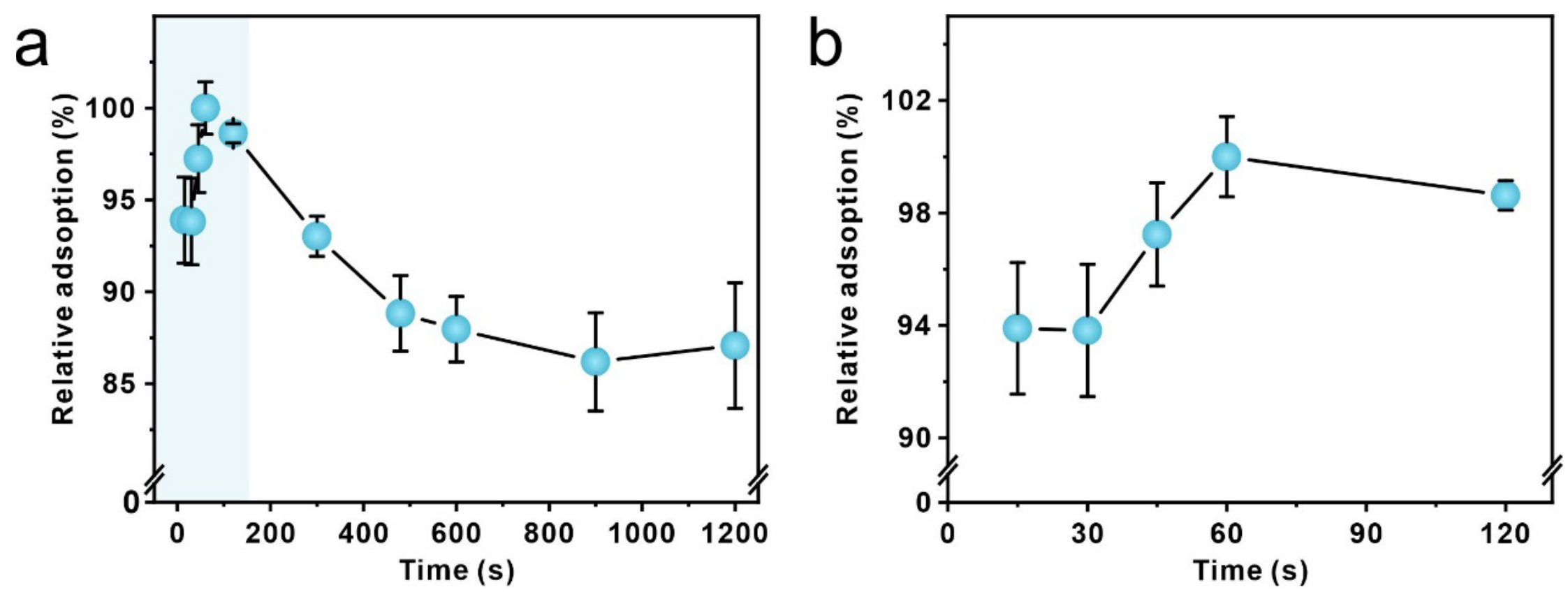

Fig. S7. Time-resolved cell adsorption on CGB. (a) The adsorption efficiency of cells on CGB measured at time points of 0, 15, 30, 60, 120, 300, 450, 600, 900, and 1200 s. (b) Enlarged view of the adsorption efficiency within the first 120 s. Error bar means ± SD (n = 5).

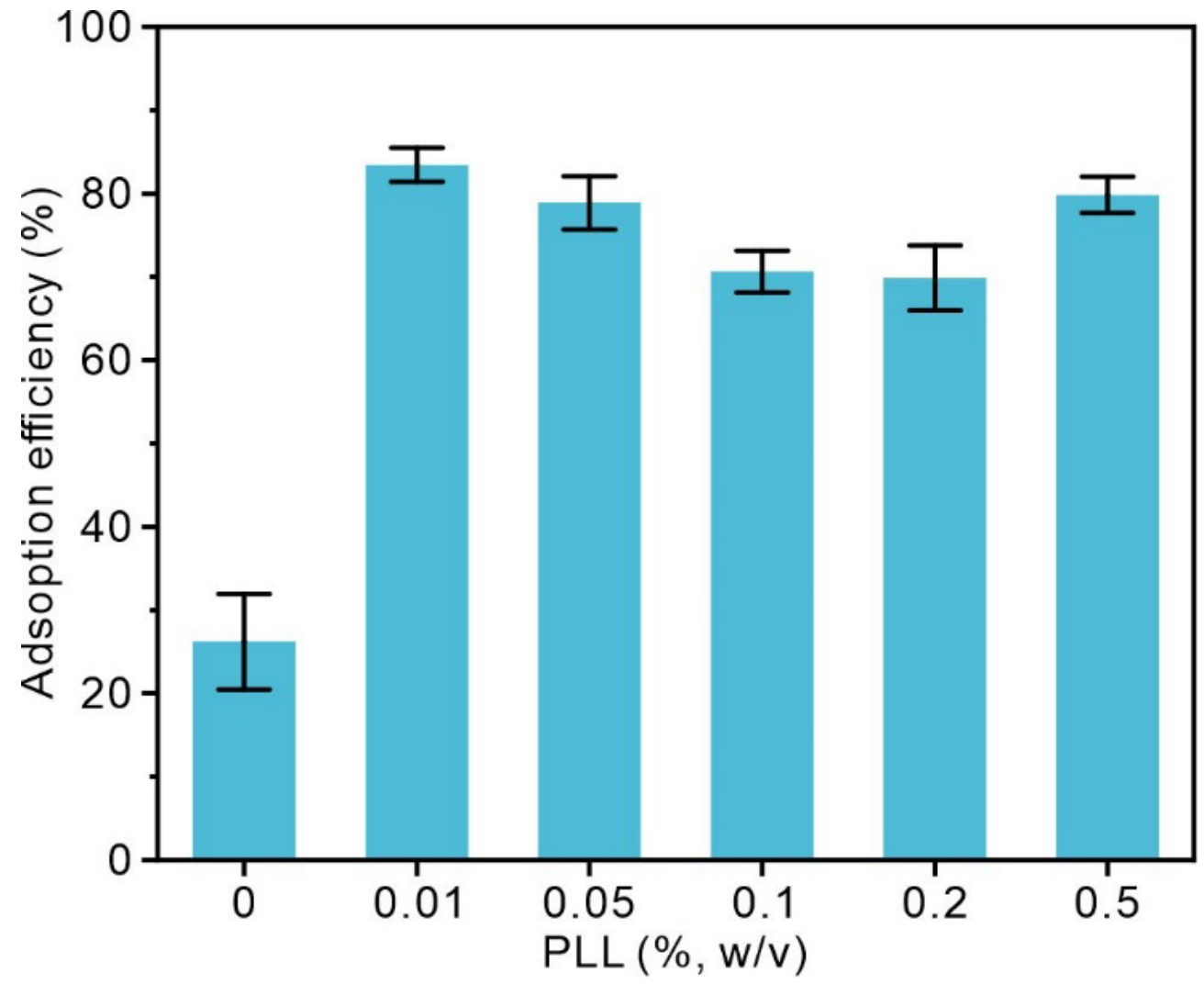

Fig. S8. Effect of PLL concentration on cell adsorption by CGB. Cell adsorption efficiency after CGB surface modification using different concentrations of PLL. Data are presented as mean ± SD (n = 5).

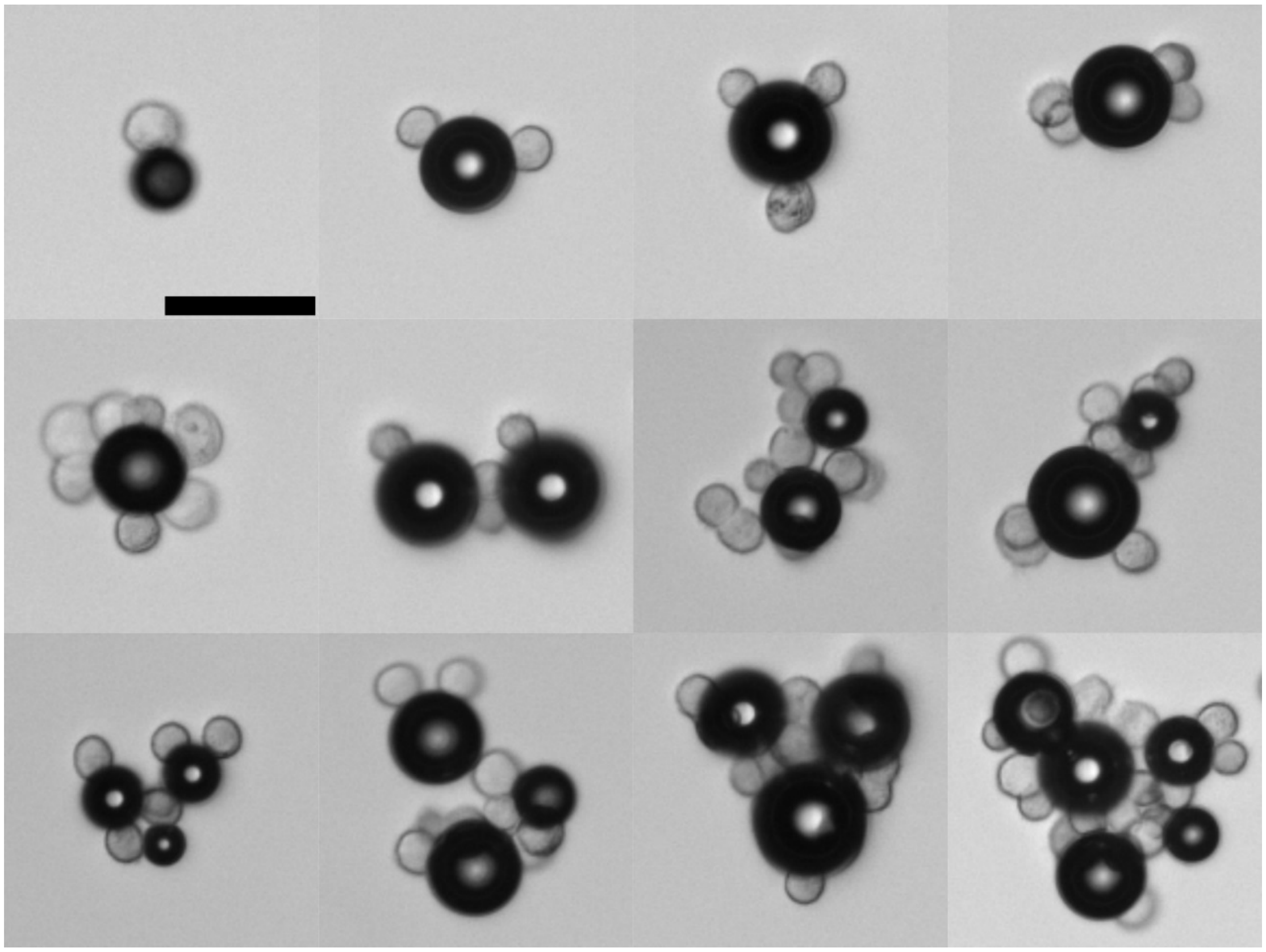

Fig. S9. CGB mediates flexible cell–particle interactions. Optical microscopy images of HeLa cells adsorbed onto CGB. Individual CGB can adsorb different numbers of cells, while a single cell can simultaneously adhere to multiple CGB. Scale bar, 40 μm.

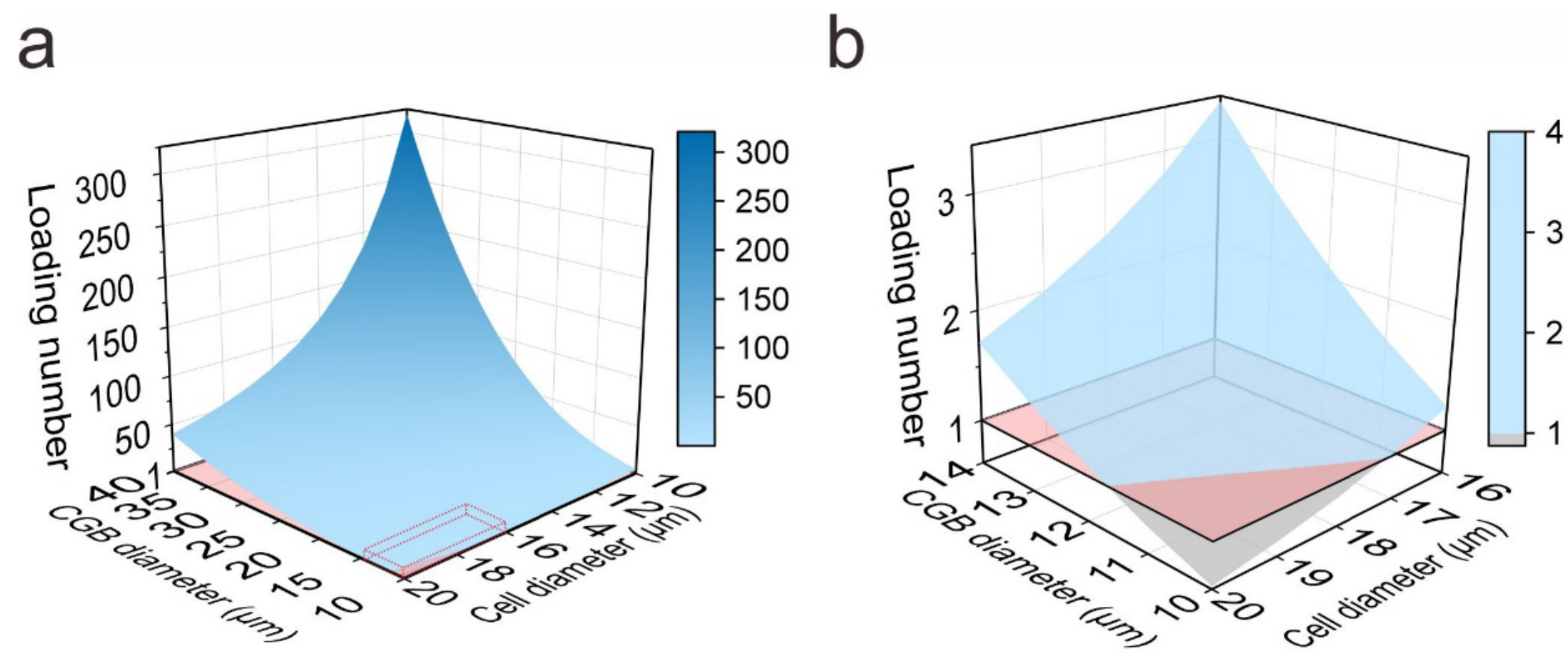

Fig. S10. Theoretical analysis of CGB cell load limits. (a) A theoretical calculation function graph illustrating the maximum number of 10-20 μm cells that can adhere to CGB with diameters ranging from 10 to 40 μm. (b) Magnified image of 10-14 μm CGB adsorbing 16-20 μm cells. A carrying number less than one indicates that one CGB is unable to carry a single cell to float.

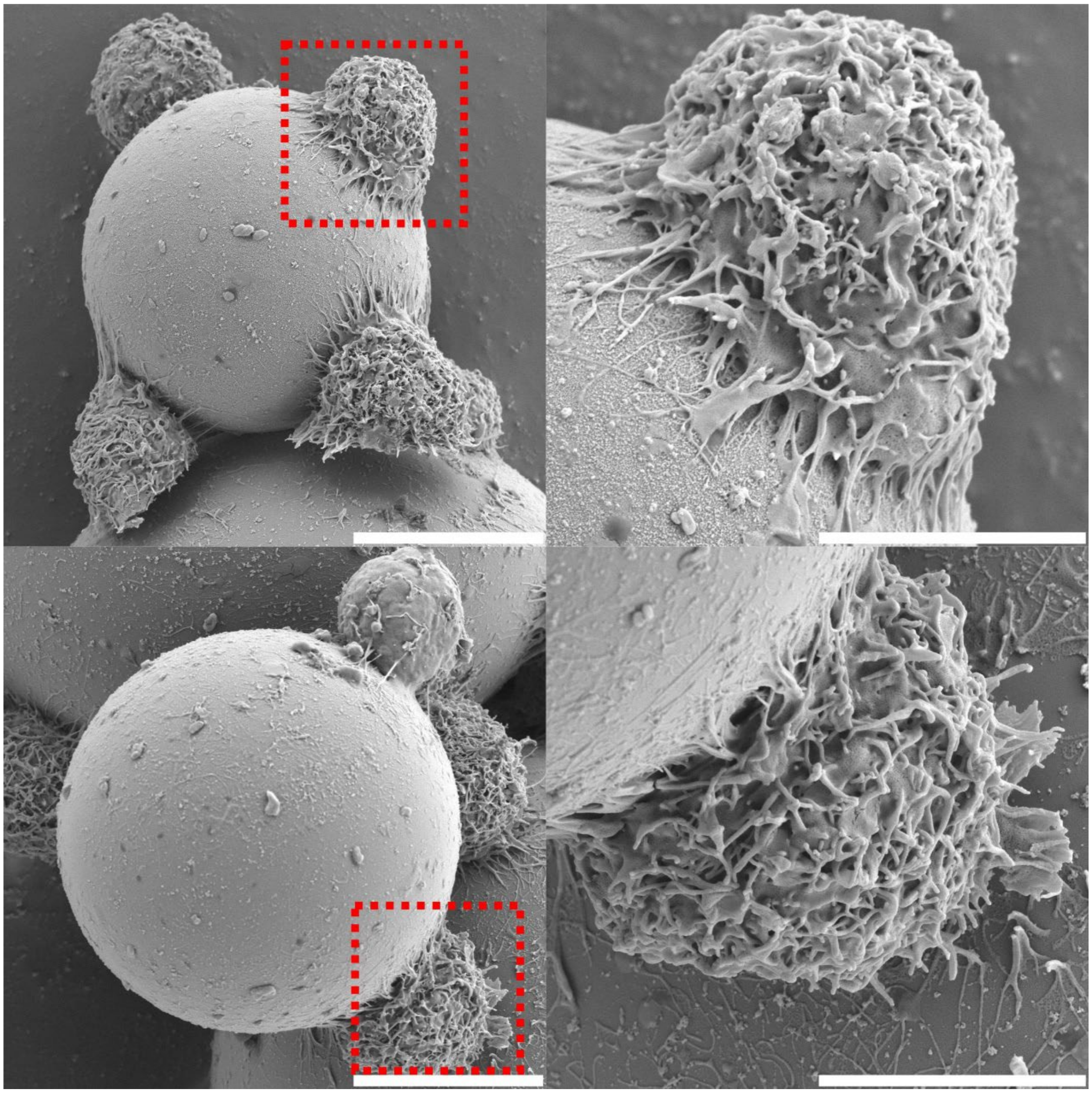

Fig. S11. SEM images of HeLa cells adsorbed onto CGB. Cells were clearly found to adhere stably between two CGB, and the magnified view showed the pseudopodia structures formed by the cells on the CGB. Scale bars: 10 µm (left), 5 µm (right, enlarged view).

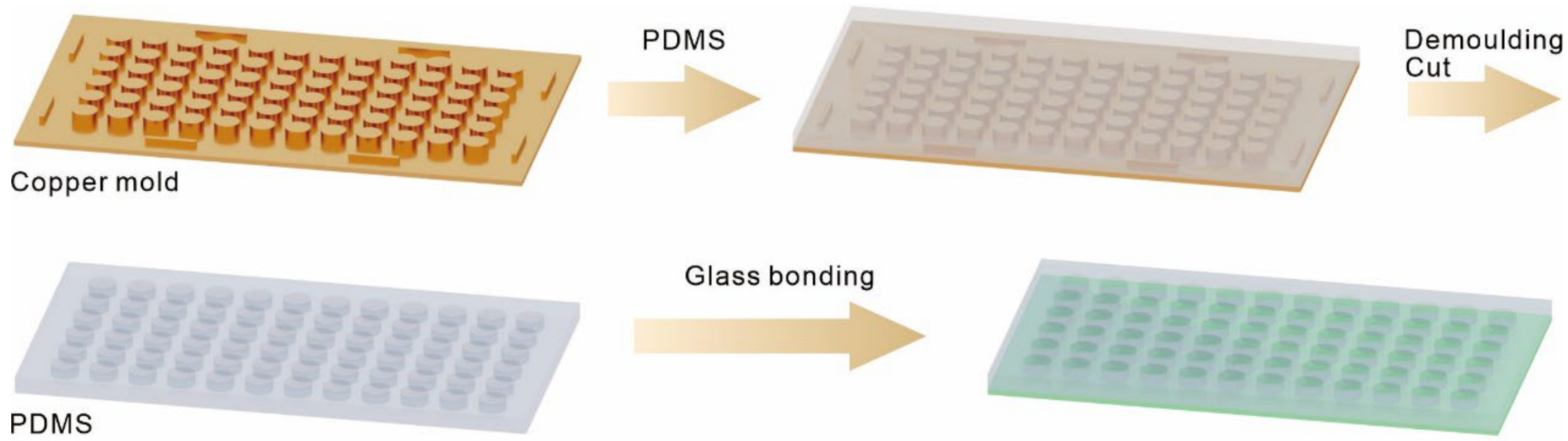

Fig. S12. Preparation workflow of the droplet microarray. A copper mold was first fabricated using standard CNC machining techniques. Then, PDMS prepolymers were cast into this mold for curing. After demolding and being precisely cut into the microarray structure, the PDMS microarray was bonded to a glass slide via plasma irradiation to enhance the overall rigidity and stability.

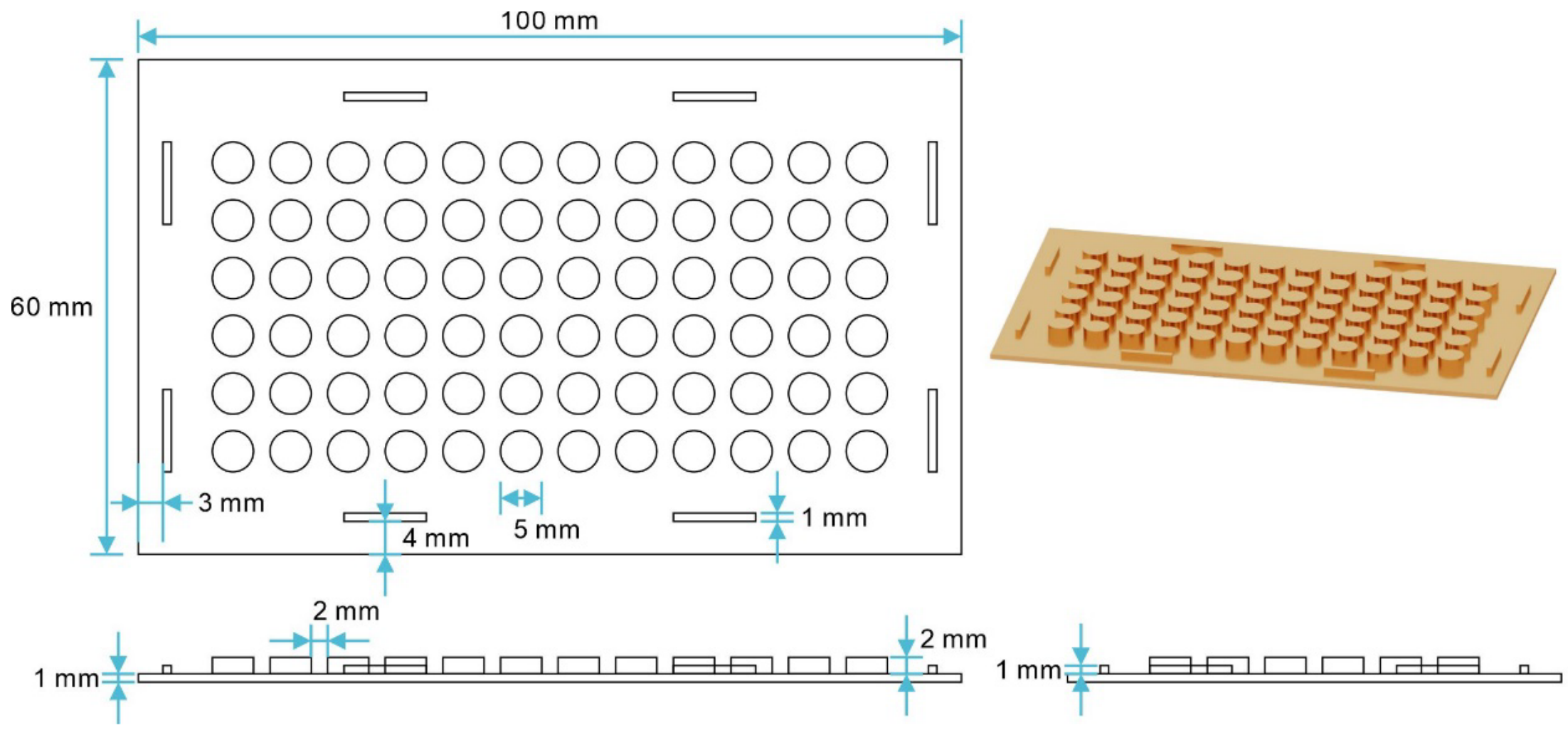

Fig. S13. Parameters of the copper mold. Each culture well was designed with a diameter of 5 mm and a depth of 2 mm, and an interspace of 2 mm between adjacent wells. A peripheral rectangular protrusion serves as the boundary line for cutting, ensuring the consistency of the droplet microarray. The final dimensions of the microarray are 94 mm × 52 mm.

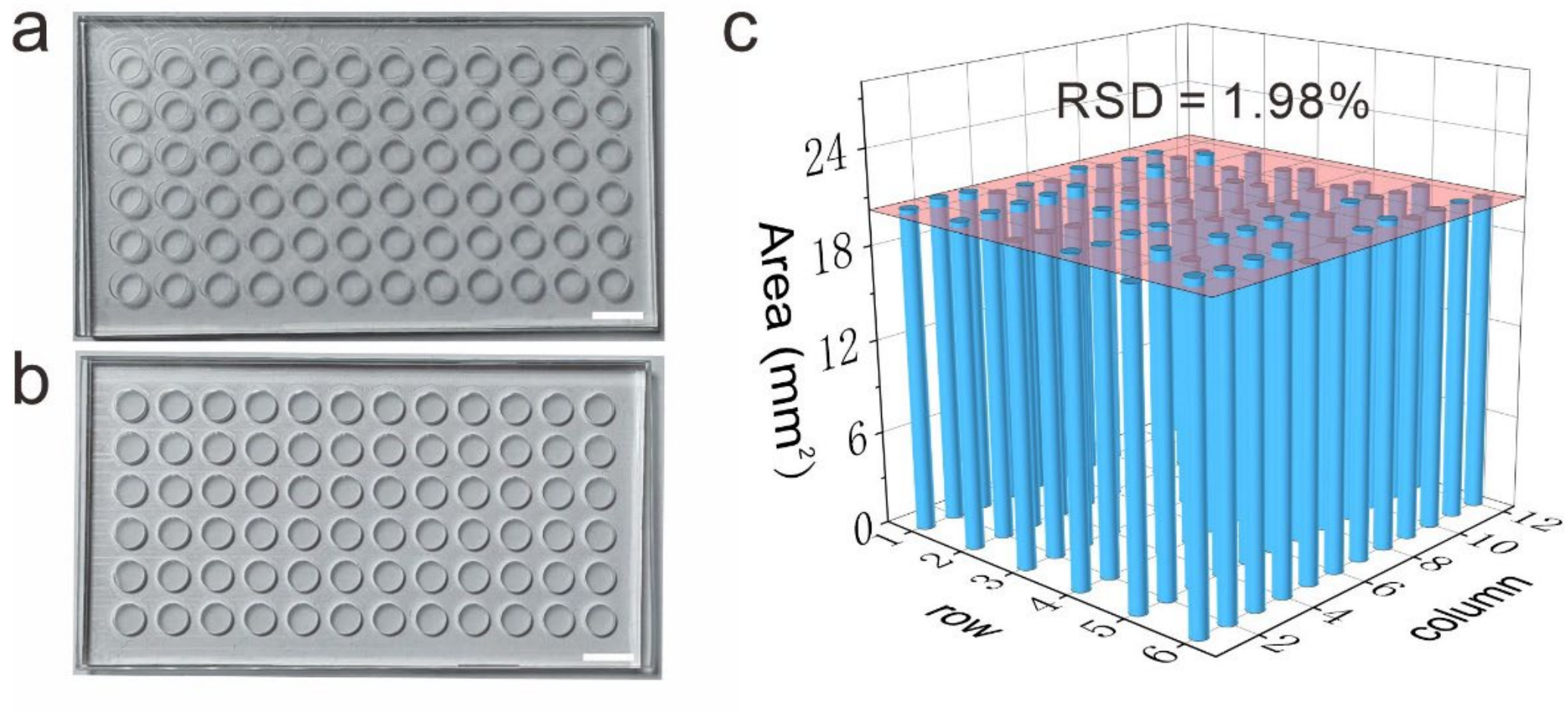

Fig. S14. Microarray well geometry and size distribution. (a, b) Photographs of the droplet microarray chip by top view (a) and bottom view (b). Scale bars: 10 mm (c) Histogram showing the size distribution of each well in the microarray chip. The bottom area of the well was 20.2 ± 0.4 mm^2 (mean ± SD), with a relative standard deviation (RSD) of 1.98%.

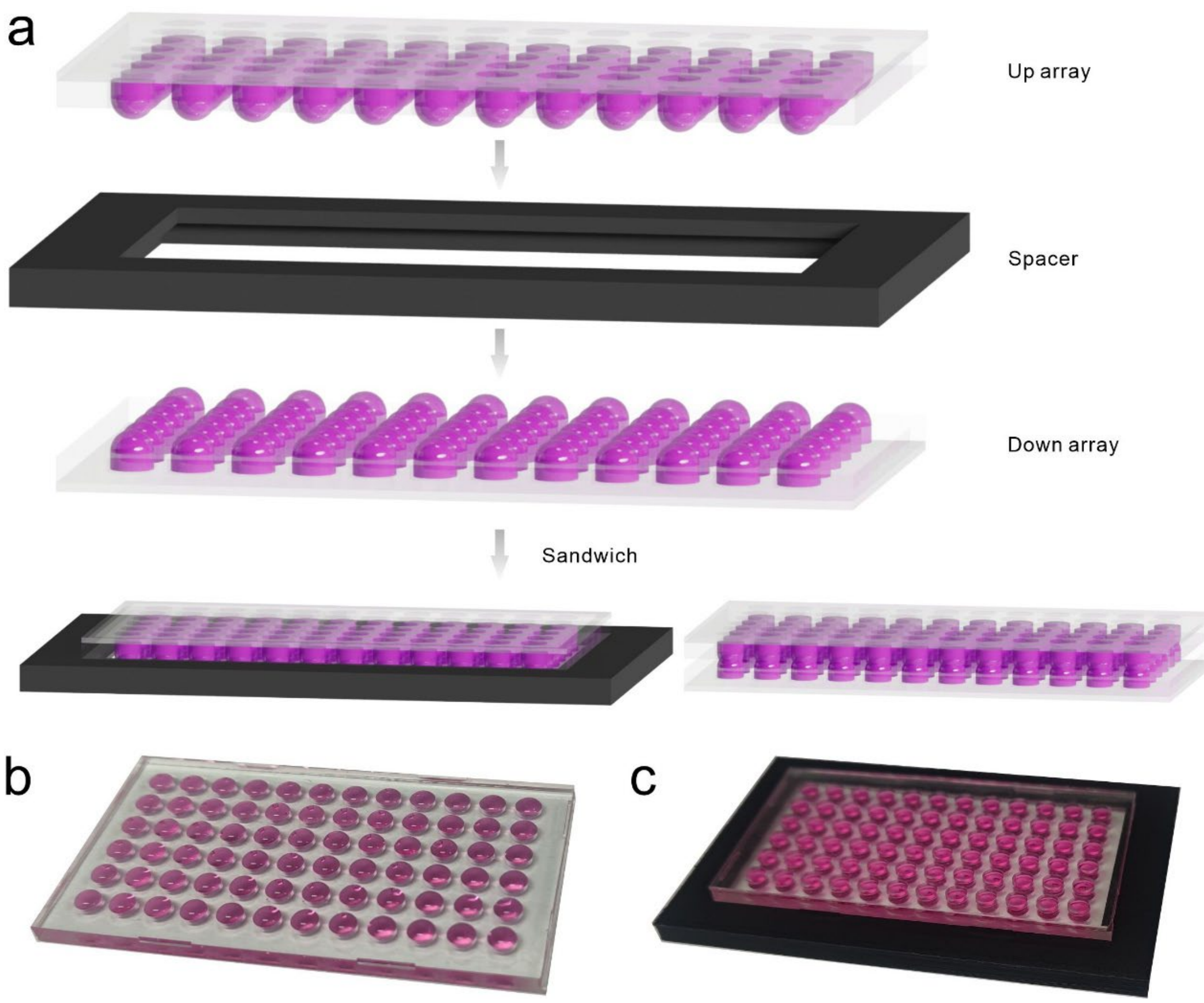

Fig. S15. Droplet microarray sandwich. (a) Schematic of the workflow in kiss between up and down droplets for sandwiching, where the distance between droplets was controlled by a 3D-printed spacer. (b) A photograph of the droplet microarray. (c) A photograph of the droplet sandwich.

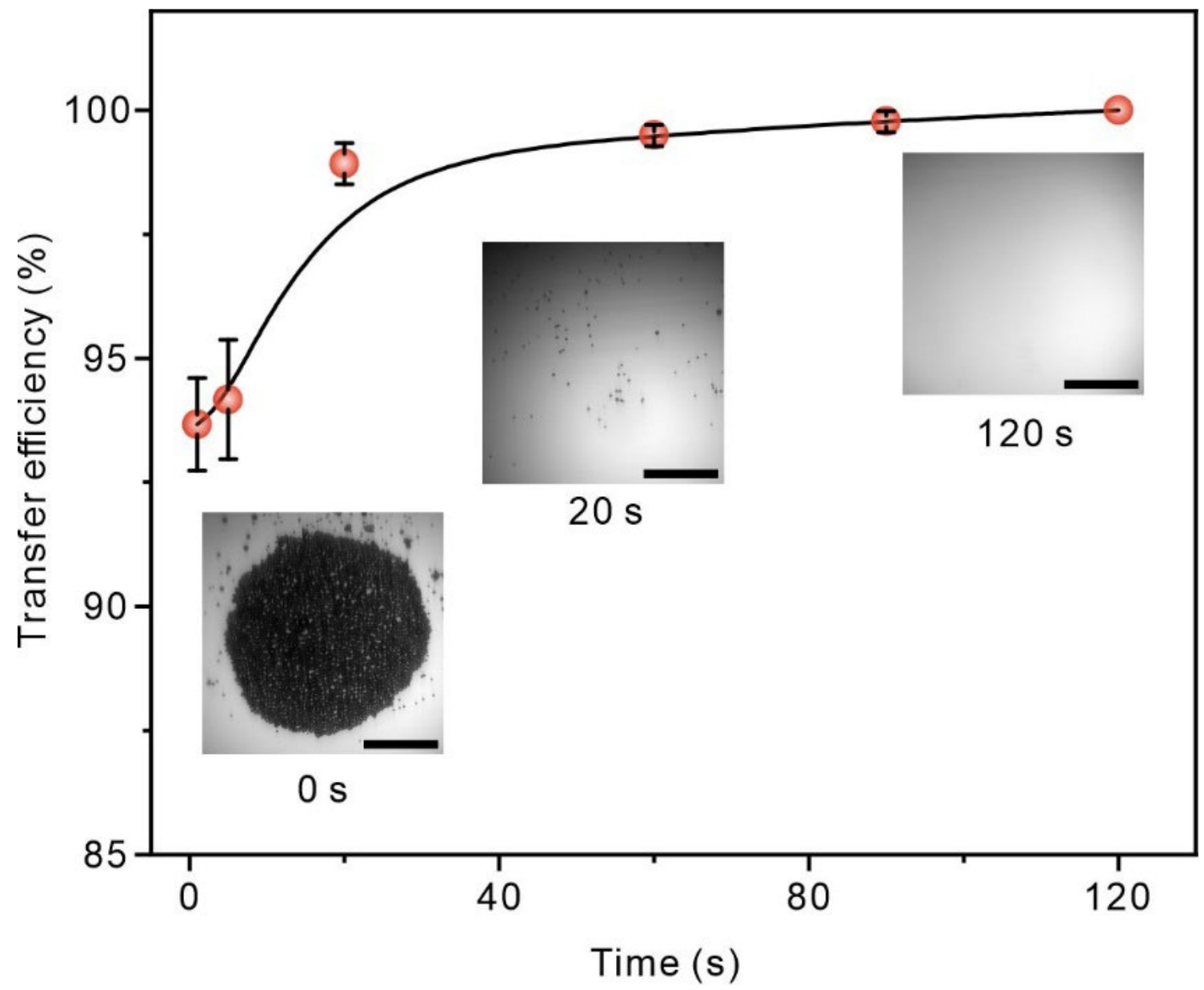

Fig. S16. Sandwich transfer efficiency of CGB without cells at different times. The transfer efficiency was calculated by recording the remaining number of CGB in the droplets before and after sandwich transfer at intervals of 1, 5, 20, 60, 90, and 120 s. Scale bars: 500 µm. Error bar means ± SD (n = 5).

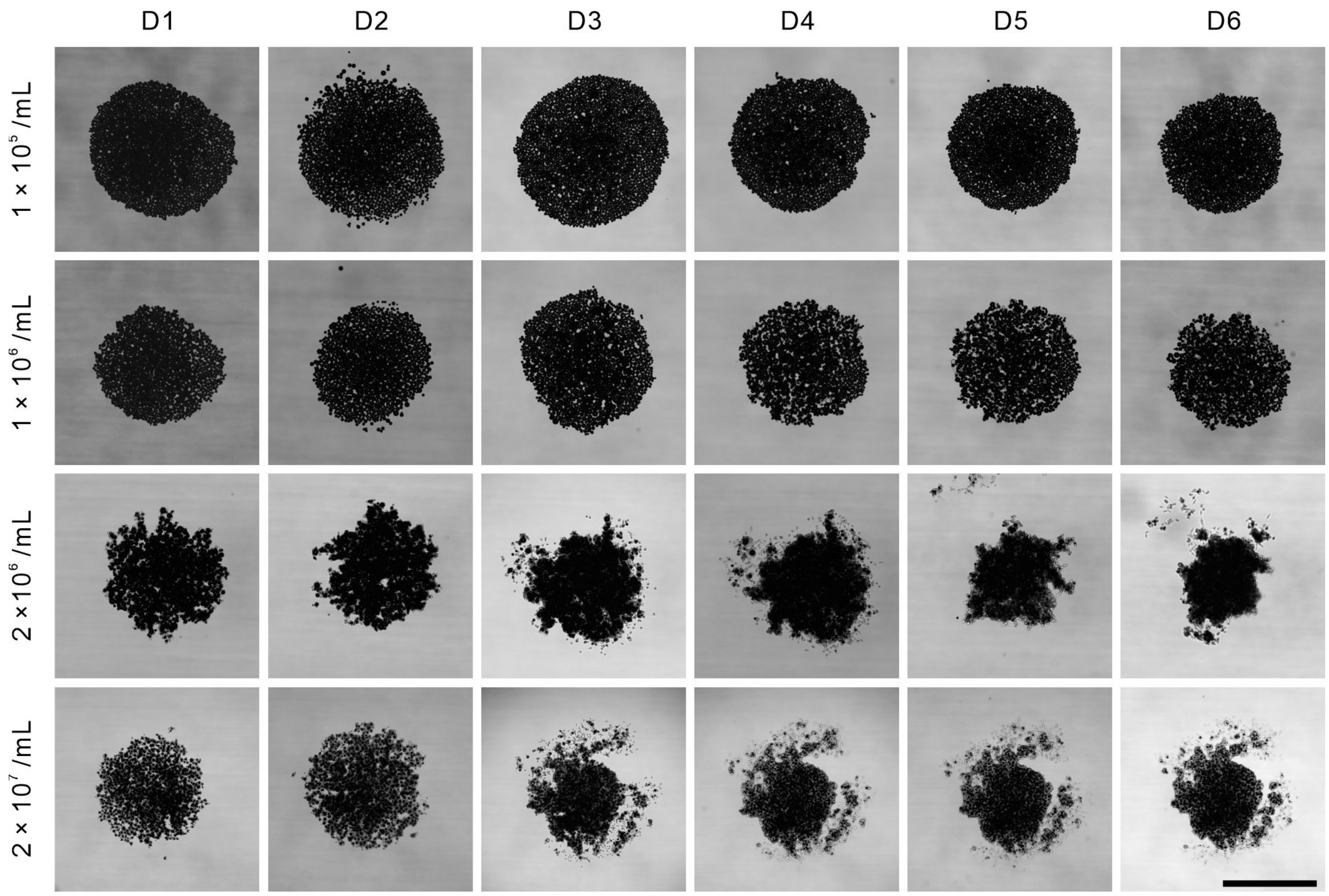

Fig. S17. Effect of initial cell density on BFC spheroid formation. Representative bright-field images showing the growth of BFC cultures generated by mixing approximately 10^5 CGBs with HeLa cells at initial concentrations of 1×10^5, 1×10^6, 2×10^6, and 2×10^7 cells/mL, corresponding to 5×10^3, 5×10^4, 1×10^5, and 1×10^6 cells, respectively, in a 50 µL attachment system, from D1 to D6. Scale bars, 400 µm.

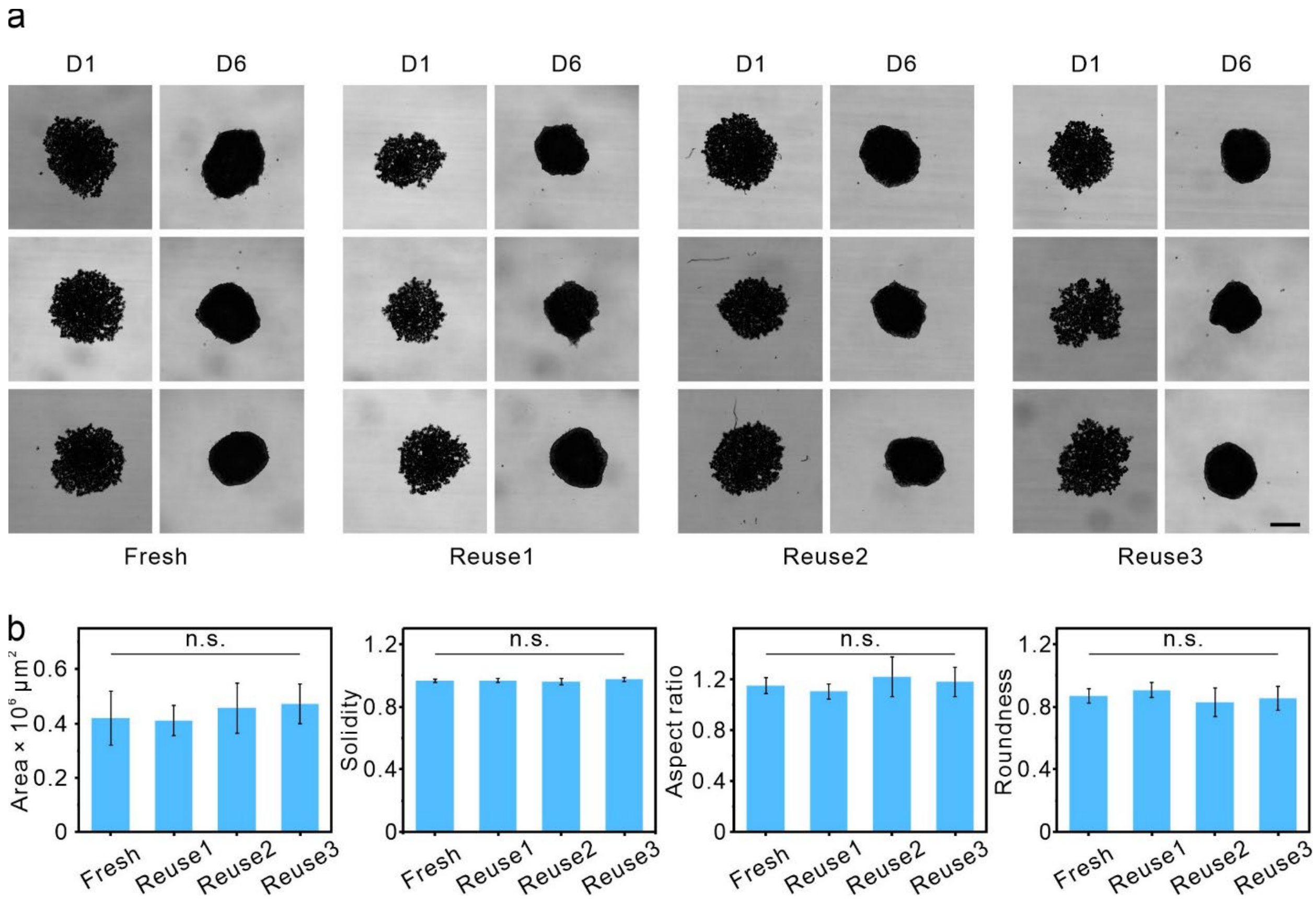

Fig. S18. Validation of BFC array chip reusability after repeated sterilization and reuse. (a) Representative bright-field images of HCT116 BFC spheroids cultured on fresh chips and reused chips after the first, second, and third reuse cycles at D1 and D6. Scale bar: 400 µm. (b) Quantitative comparison of spheroid area, solidity, aspect ratio, and roundness among the fresh, reuse 1, reuse 2, and reuse 3 chip groups. Data are presented as mean ± SD ($n = 10$). Statistical significance was assessed using one-way repeated-measures ANOVA with Geisser–Greenhouse correction followed by Šídák’s multiple-comparisons test for the indicated comparisons; n.s., no significant difference, $p > 0.05$.

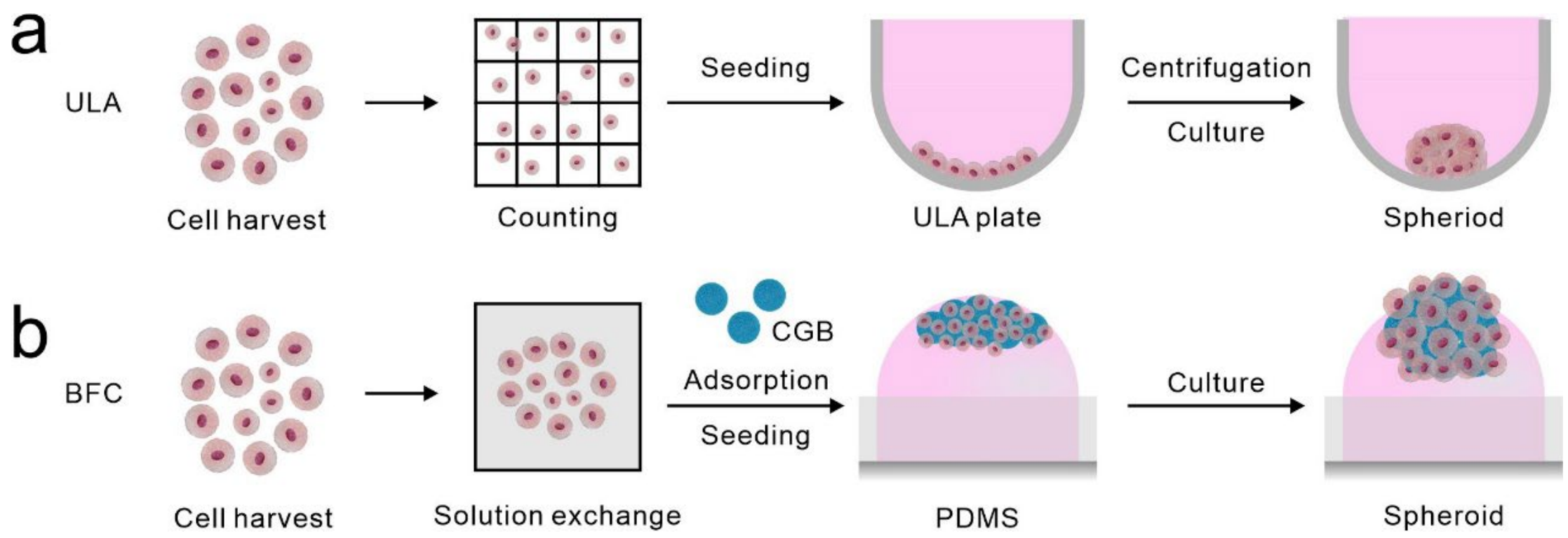

Fig. S19. Schematic illustration of BFC and ULA culture. (a) For ULA method, after harvesting the cells, it was necessary to determine the size of the cell-spheroid by counting. After cell seeding into the ULA plate, centrifugation was performed to enable the dispersed cells gathering at the center of the well bottom, followed by further cultivation to form cell spheroids. (b) For BFC method, following cell harvest, the culture medium was replaced with an attachment solution. An appropriate amount of CGB was then added, and the mixture was gently agitated to ensure even distribution before seeding. The cells that accumulated at the top of the droplets would spontaneously grow into spheroids.

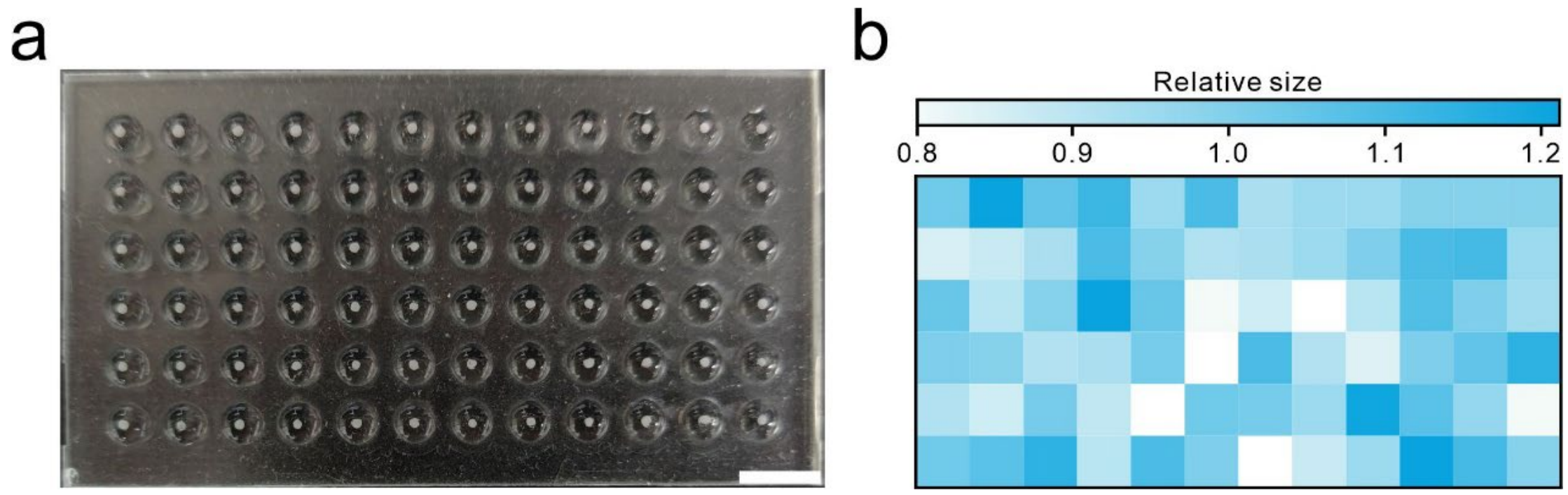

Fig. S20. Size distribution of CGB clusters in the droplet microarray. (a) Photographic representation of CGB clusters in a droplet microarray. Scale bar: 10 mm. (b) Heatmap showing the relative sizes of CGB clusters in the droplet microarray. The size of CGB clusters was 1.388 ± 0.15 mm^2 (mean ± SD), with RSD of 9.8%.

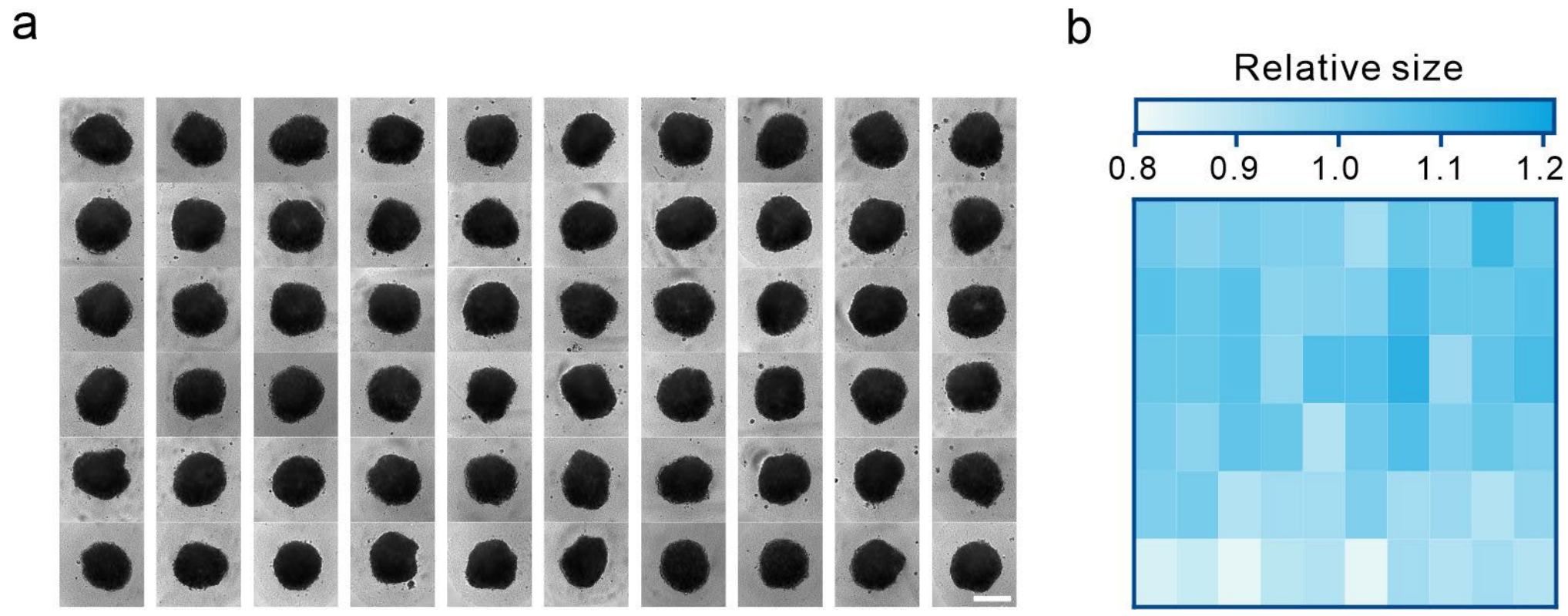

Fig. S21. Size distribution of cell spheroids cultured using a ULA culture plate. (a) Microscopic images of HCT116 cell spheroids cultured using a ULA culture plate. Scale bar: 400 μm. The average size of cell spheroids was 0.26 ± 0.02 mm^2 (mean ± SD), RSD = 6.9%. (b) Heatmap showing the relative sizes of cell spheroids cultured using a ULA culture plate.

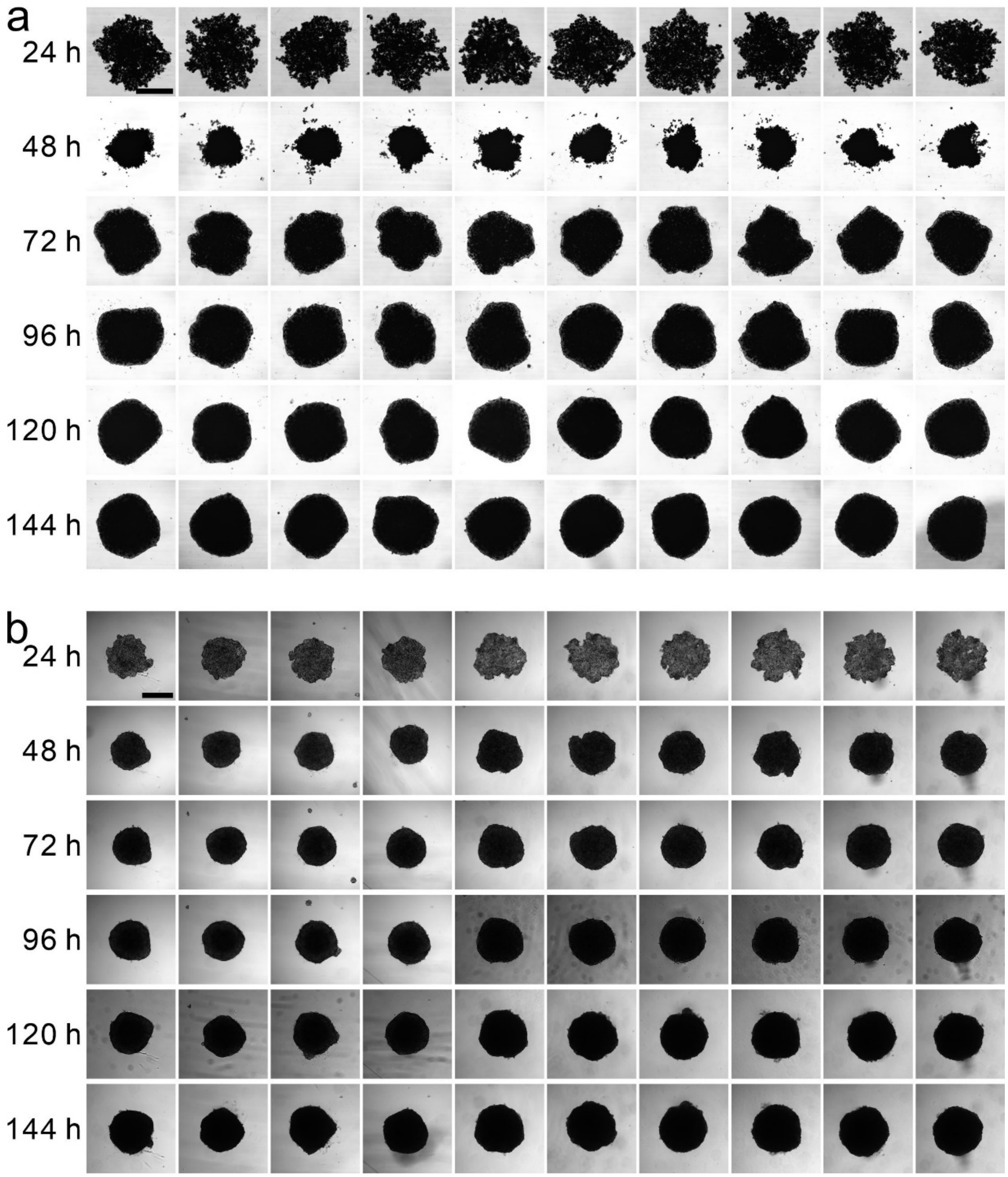

Fig. S22. Time-dependent morphological evolution of HCT116 spheroids in BFC and ULA cultures. Morphological changes in HCT116 cells cultured for 6 days using (A) BFC and (B) ULA methods. Images acquired from 24 to 144 hours are intentionally repeated in **Fig. 3C.** Scale bar, 500 µm.

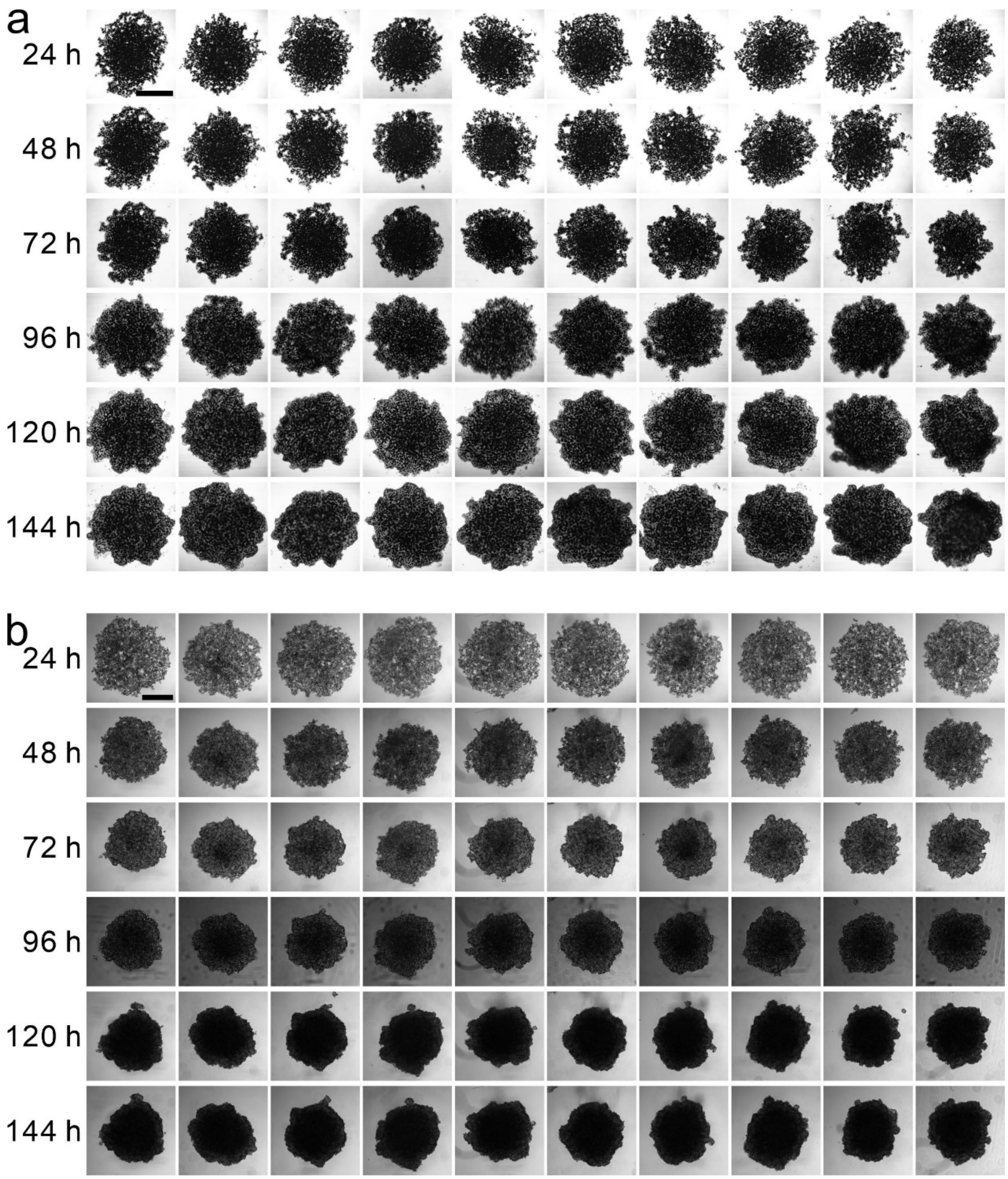

Fig. S23. Time-dependent morphological evolution of HeLa spheroids in BFC and ULA cultures. Morphological changes in HeLa cells cultured for 6 days using (A) BFC and (B) ULA methods. Scale bar, 500 μm.

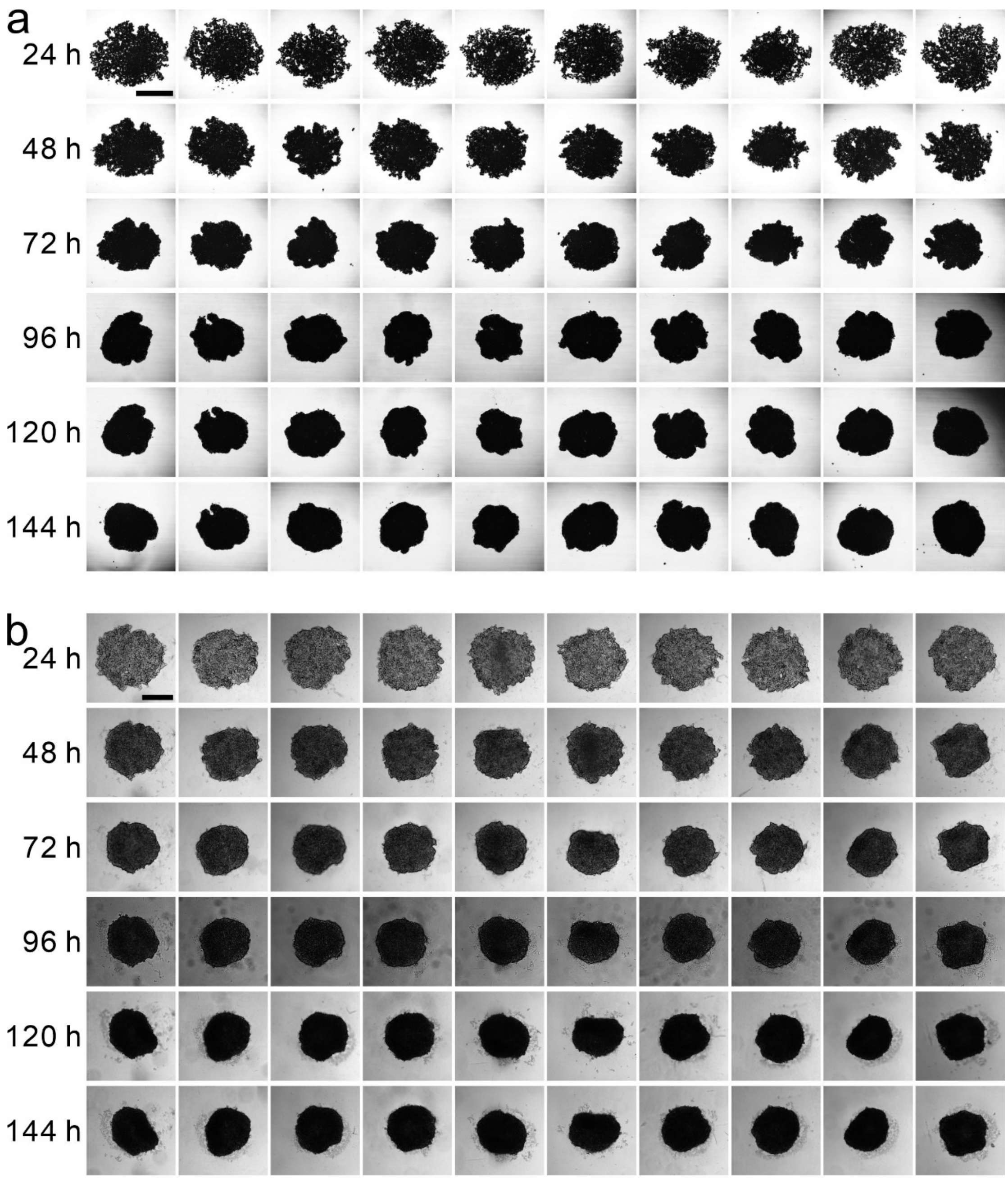

Fig. S24. Time-dependent morphological evolution of A549 spheroids in BFC and ULA cultures. Morphological changes in A549 cells cultured for 6 days using (A) BFC and (B) ULA methods. Scale bar, 500 μm.

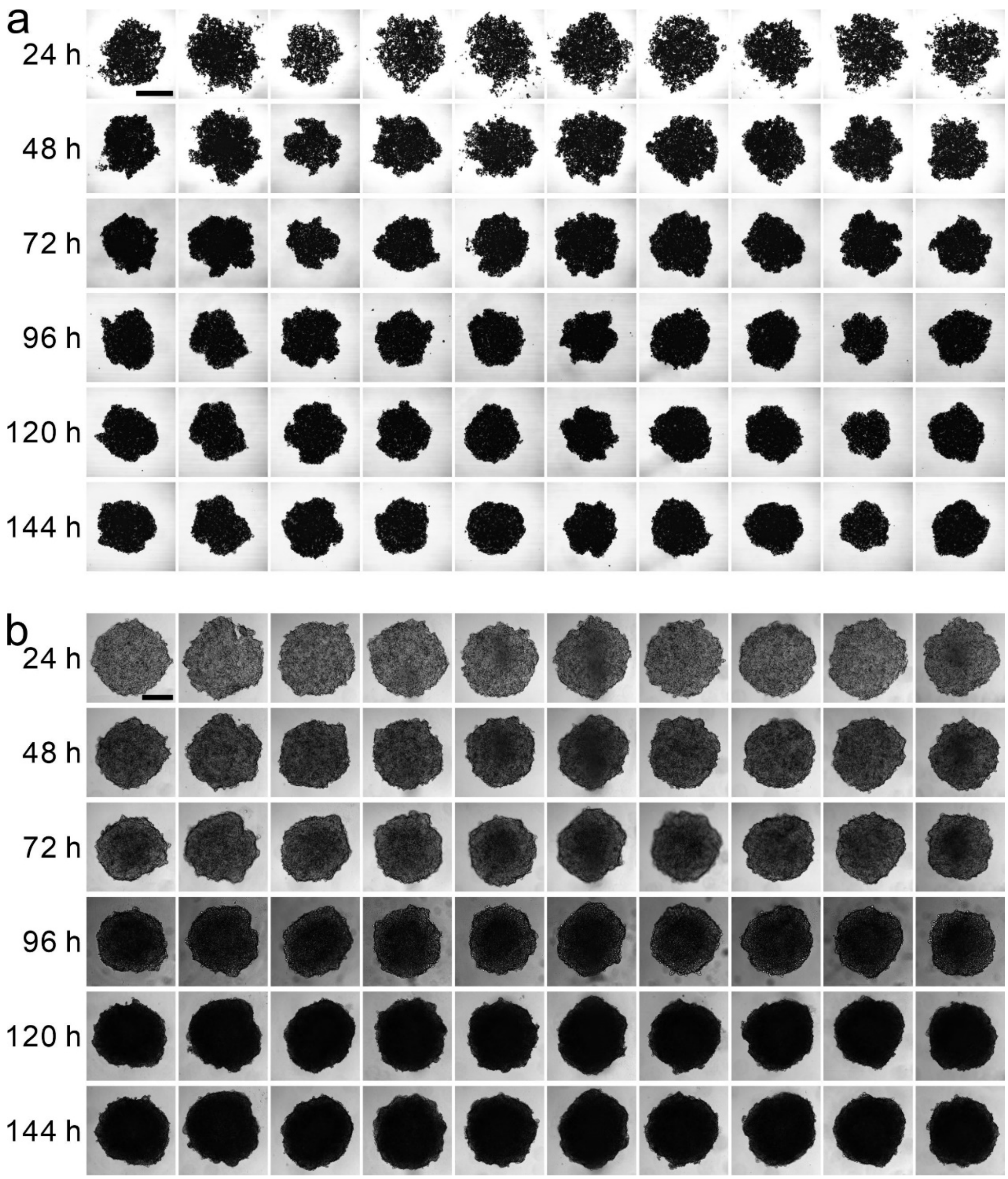

Fig. S25. Time-dependent morphological evolution of MDA-MB-231 spheroids in BFC and ULA cultures. Morphological changes in MDA-MB-231 cells cultured for 6 days using (A) BFC and (B) ULA methods. Scale bar, 500 μm.

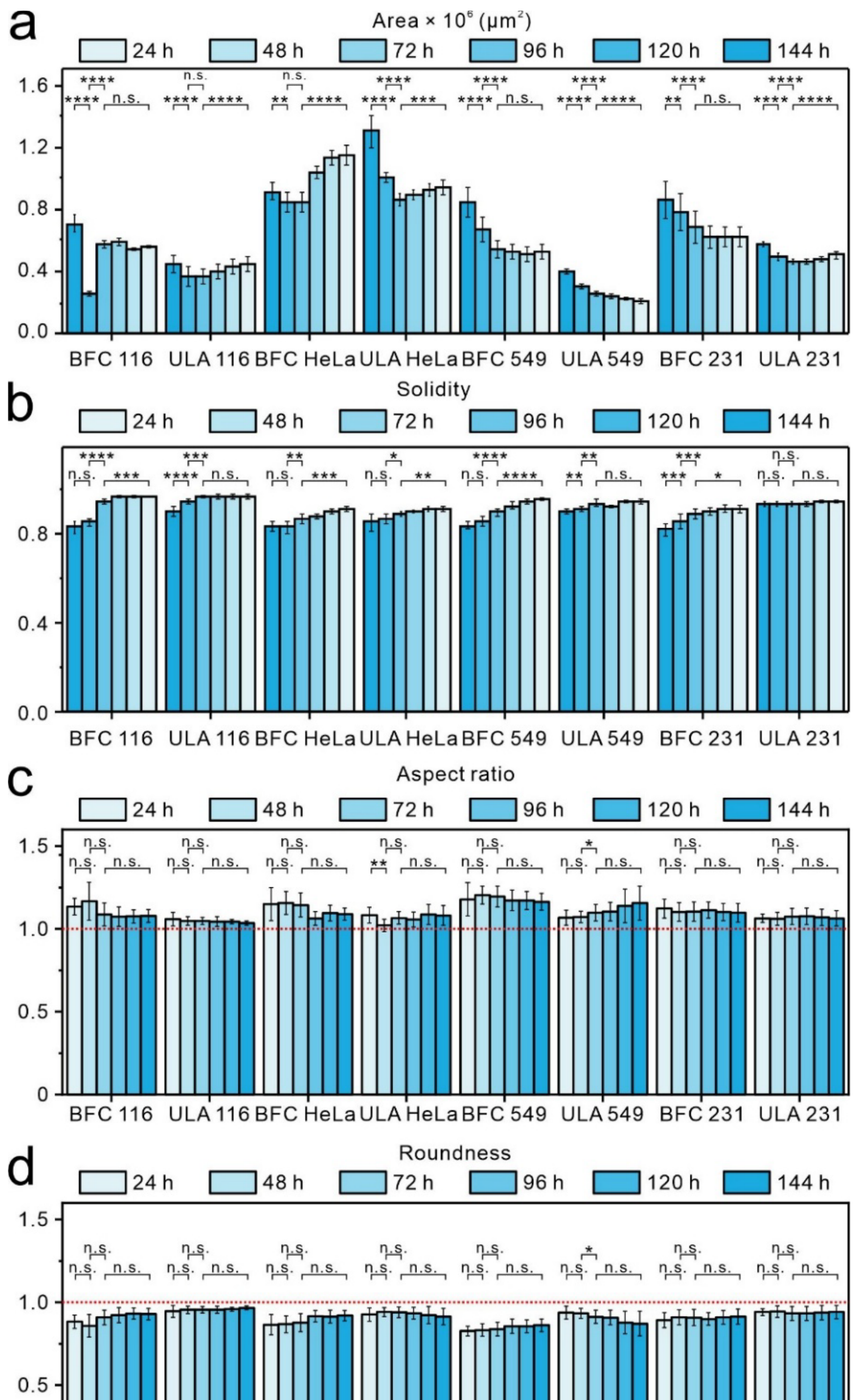

Fig. S26. Quantitative analysis of the morphological changes in spheroids cultured using the BFC and ULA methods. Time-dependent changes in the projected area (a), solidity (b), aspect ratio (c), and roundness (d) of MDA-MB-231, A549, HeLa, and HCT116 spheroids cultured using the BFC and ULA methods from D1 (24 h) to D6 (144 h). Data are presented as mean ± SD (n = 10). Statistical significance was assessed using one-way repeated-measures ANOVA with Geisser–Greenhouse correction followed by Šídák's multiple-comparisons test for the indicated comparisons. n.s., $P > 0.05$; *$P \le 0.05$; **$P \le 0.01$; ***$P \le 0.001$; ****$P \le 0.0001$.

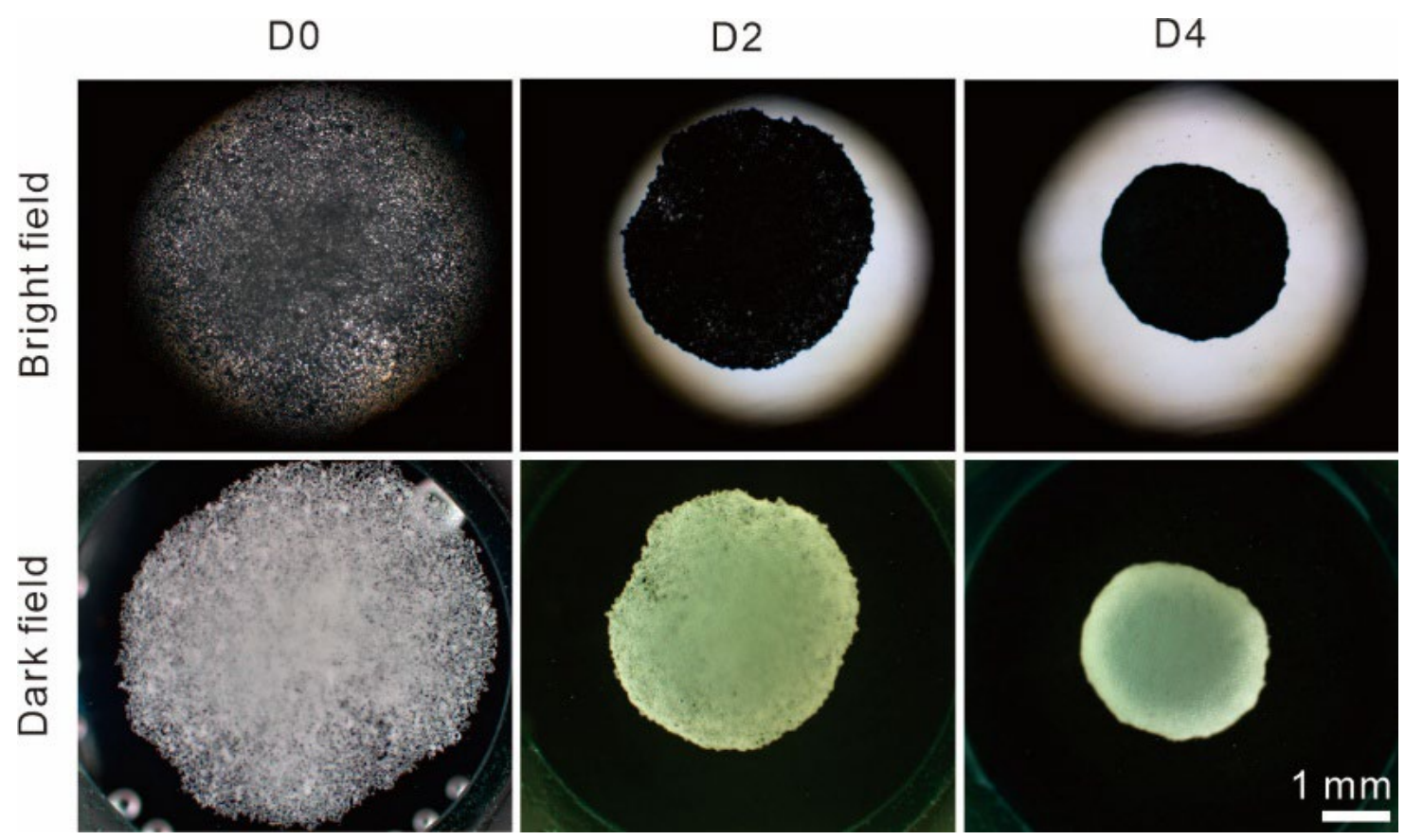

Fig. S27. BFC supports spheroid formation on enlarged culture substrates. Bright-field and dark-field microscopy images of a cell spheroid cultured on a substrate with a diameter of 7 mm at day 0 (D0), D2, and D4.

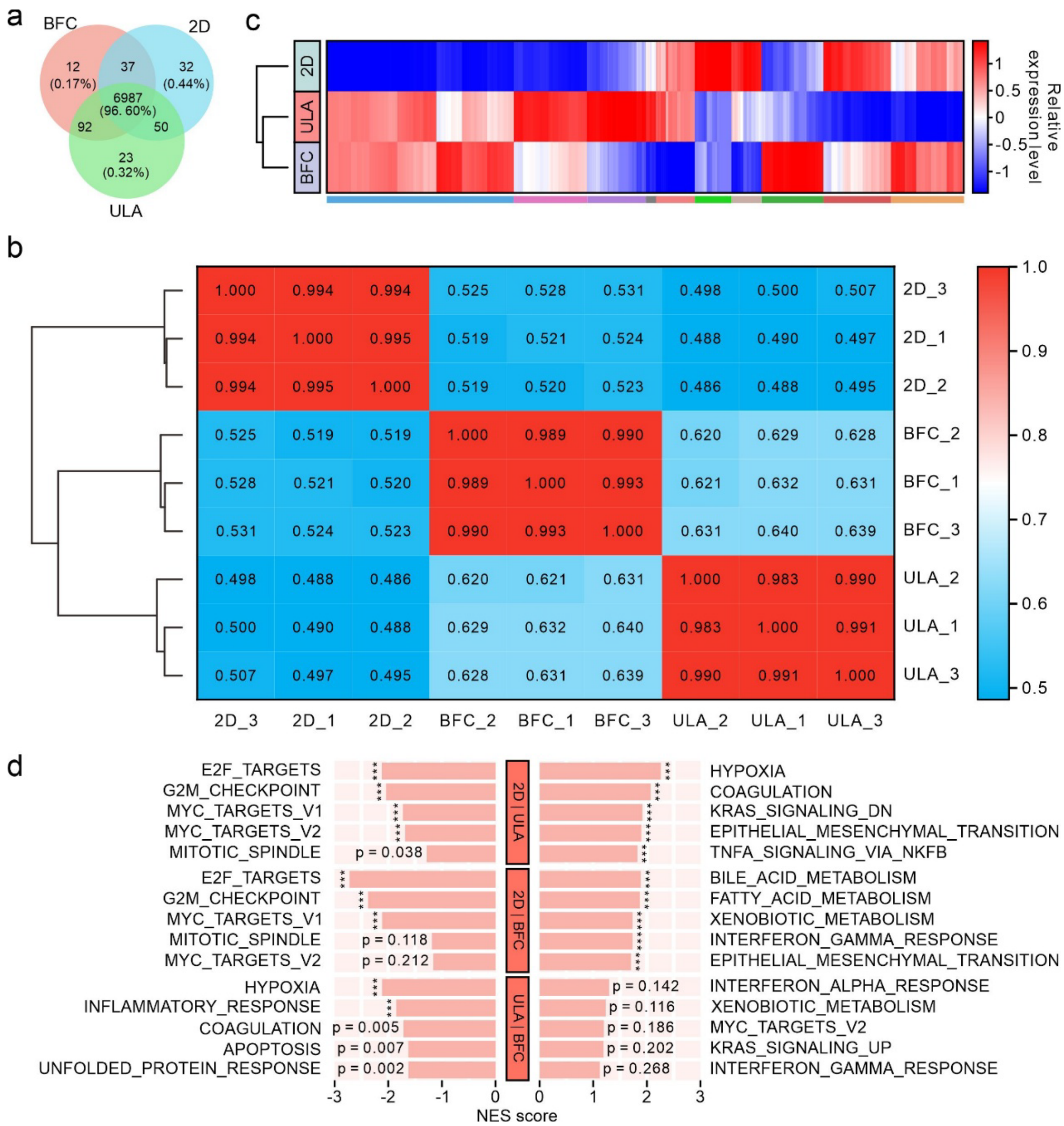

Fig. S28. Proteomic analysis of cells cultured by 2D, BFC, and ULA methods. (a) Venn diagram of proteomics data from HCT116 cells cultured by using 2D, ULA, and BFC methods. (b) Heatmap showing the correlation analysis of proteomics in samples from the 2D, ULA, and BFC culture methods. (c) Heatmap showing differences in protein expression from HCT116 cells cultured by the three methods. (d) GSEA analysis of HCT116 cells cultured by the three methods. The top five positively and negatively enriched pathways were listed. The statistical significance of the enrichment scores for each corresponding gene set was estimated by nominal p-values. *** $P < 0.001$.

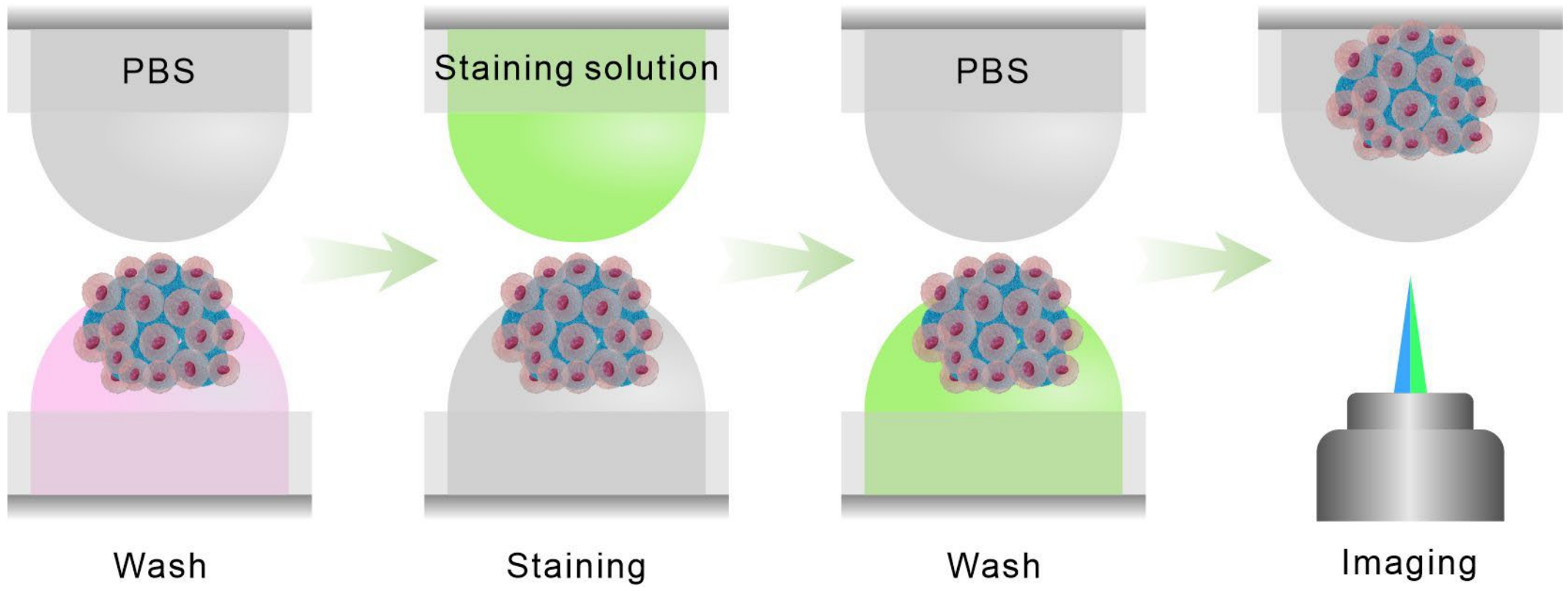

Fig. S29. Schematic illustration of staining and imaging of cell spheroids cultured using the BFC method. First, the spheroids were sandwiched between two droplets using PBS solution to ensure thorough washing of the cell spheroids. Next, the spheroids were transferred into a droplet of staining solution for incubation. After that, the spheroids underwent another round of washing by being sandwiched with PBS to remove any excess staining solution. For imaging, the droplets were inverted, and an inverted confocal microscope was used to examine the spheroids. This procedure ensures optimal imaging conditions while maintaining the structural integrity of the spheroids.

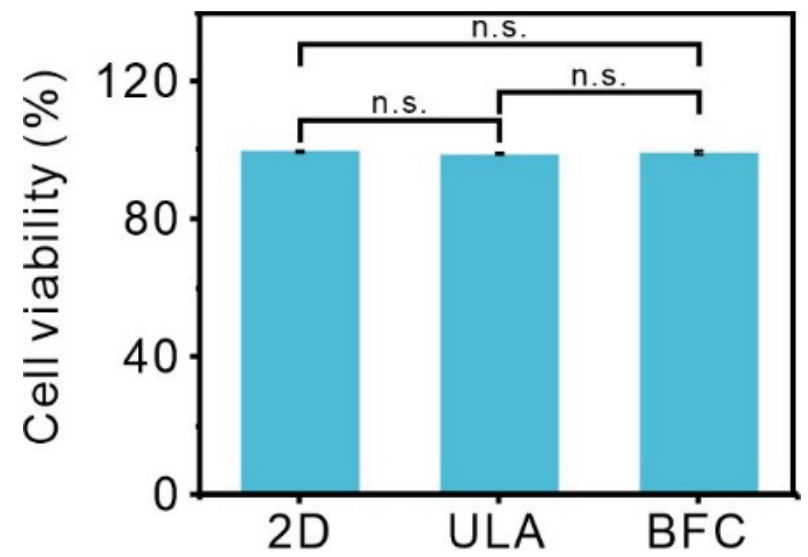

Fig. S30. HCT116 spheroids maintain comparable viability across different culture methods. Quantification of the viability of HCT116 cells cultured using the 2D, ULA, and BFC methods for 6 days, corresponding to the representative Live/Dead staining images shown in **Fig. 3J**. Cell viability was calculated as the percentage of the Calcein-AM-positive fluorescence area relative to the total Calcein-AM-positive and PI-positive fluorescence area. Data are presented as mean ± SD (n = 3). Statistical significance was assessed using one-way ANOVA followed by Tukey's multiple-comparisons test. n.s., $P > 0.05$.

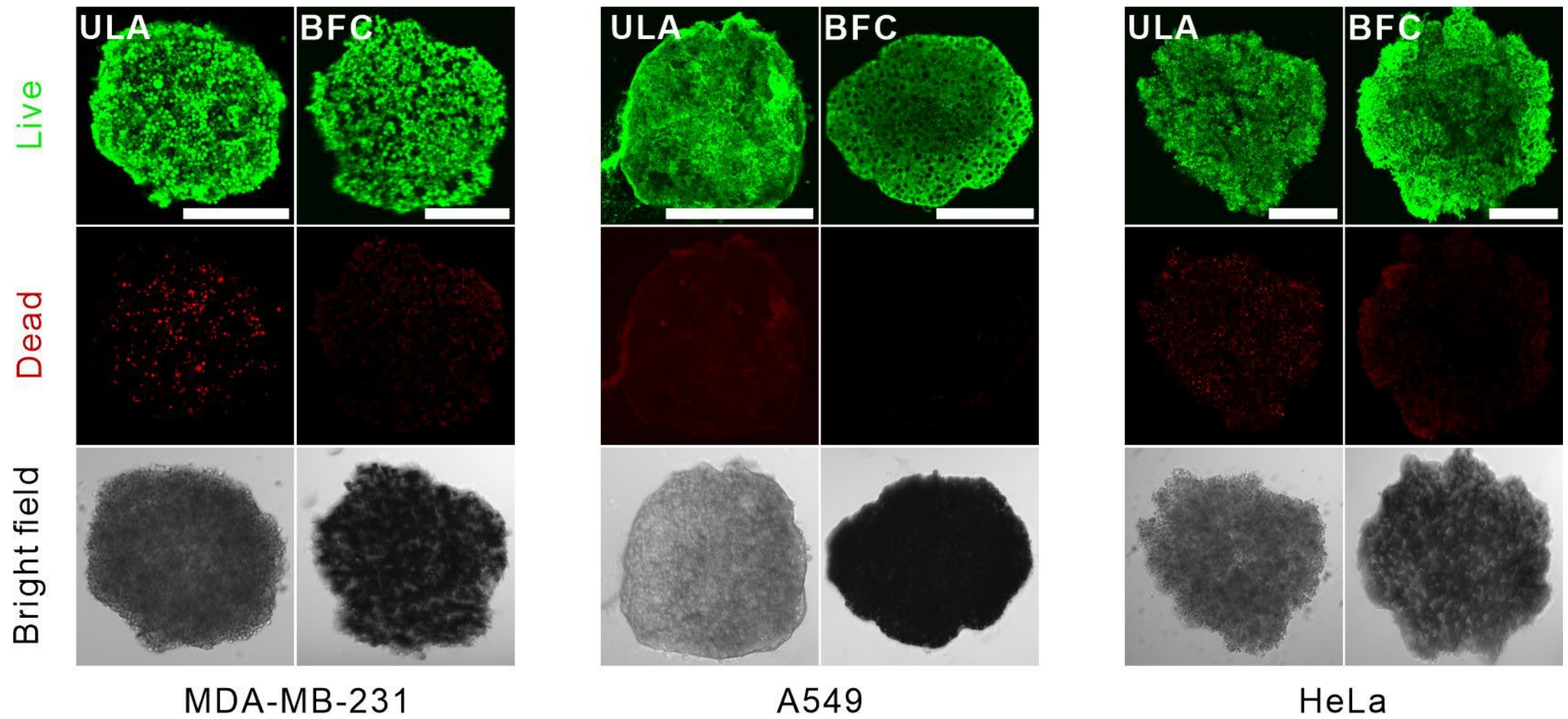

Fig. S31. Live/Dead staining confirms the viability of multiple cell types in ULA and BFC spheroids. Representative Live/Dead staining images of MDA-MB-231, A549, and HeLa cells cultured using the ULA and BFC methods. Scale bars, 400 µm.

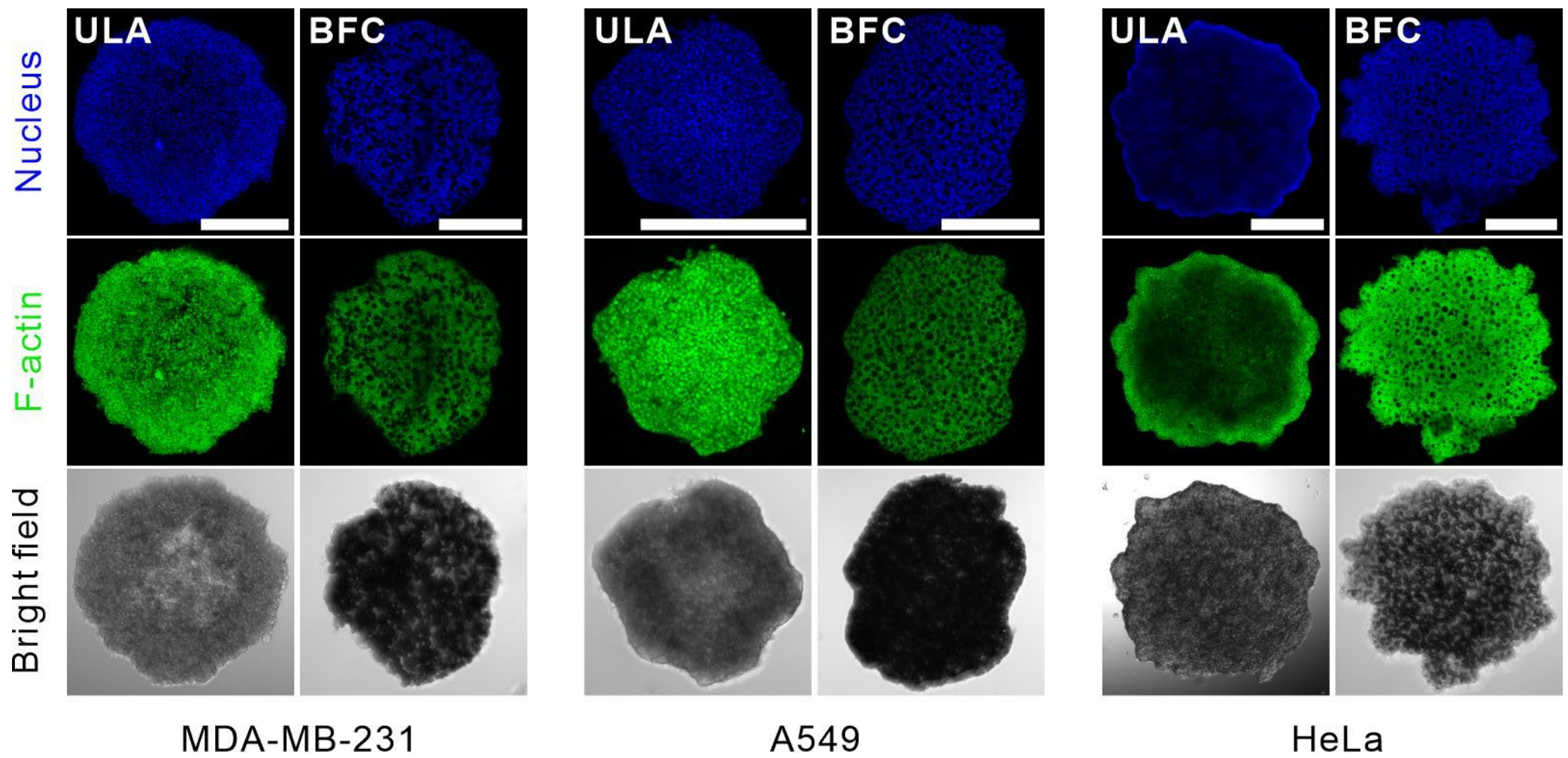

Fig. S32. Cytoskeletal and nuclear organization of multiple cell types in ULA and BFC spheroids. Representative immunofluorescence images of MDA-MB-231, A549, and HeLa cells cultured using the ULA and BFC methods. Scale bars, 400 µm.

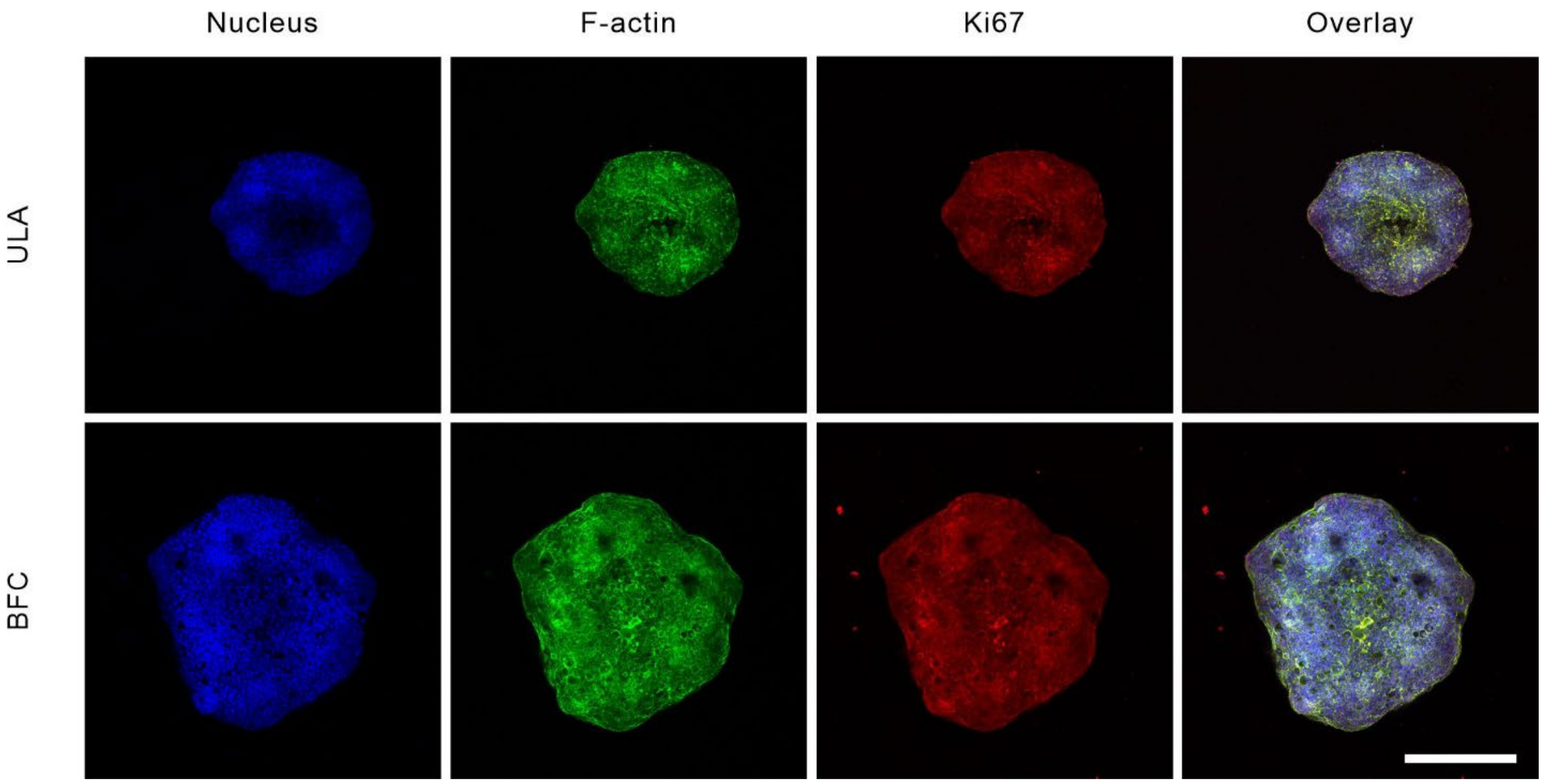

Fig. S33. Proliferative and cytoskeletal organization of ULA and BFC spheroids. Representative fluorescence images of ULA and BFC spheroids stained for nuclei, F-actin, and Ki67 (Thermo Fisher Scientific, MA5-15690). Scale bars, 400 μm.

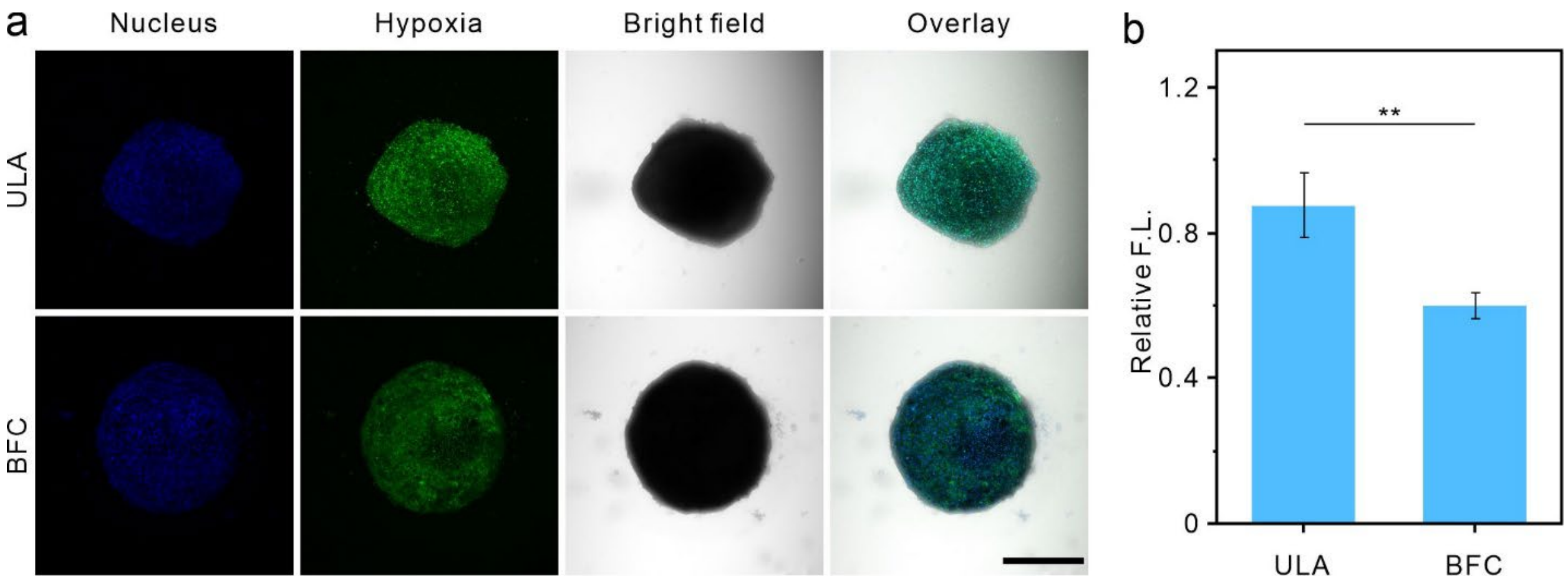

Fig. S34. Hypoxia staining of ULA and BFC spheroids. (a) Representative fluorescence images of ULA and BFC spheroids stained for nuclei and hypoxia. Hypoxia was detected using Image-iT Green Hypoxia Reagent (Thermo Fisher Scientific, I14834). Scale bars: 400 μm. (b) Quantification of hypoxia fluorescence intensity in ULA and BFC spheroids. Data are presented as mean ± SD (n = 5). Statistical analysis was performed using Student's t-test; $^{**}P < 0.01$.

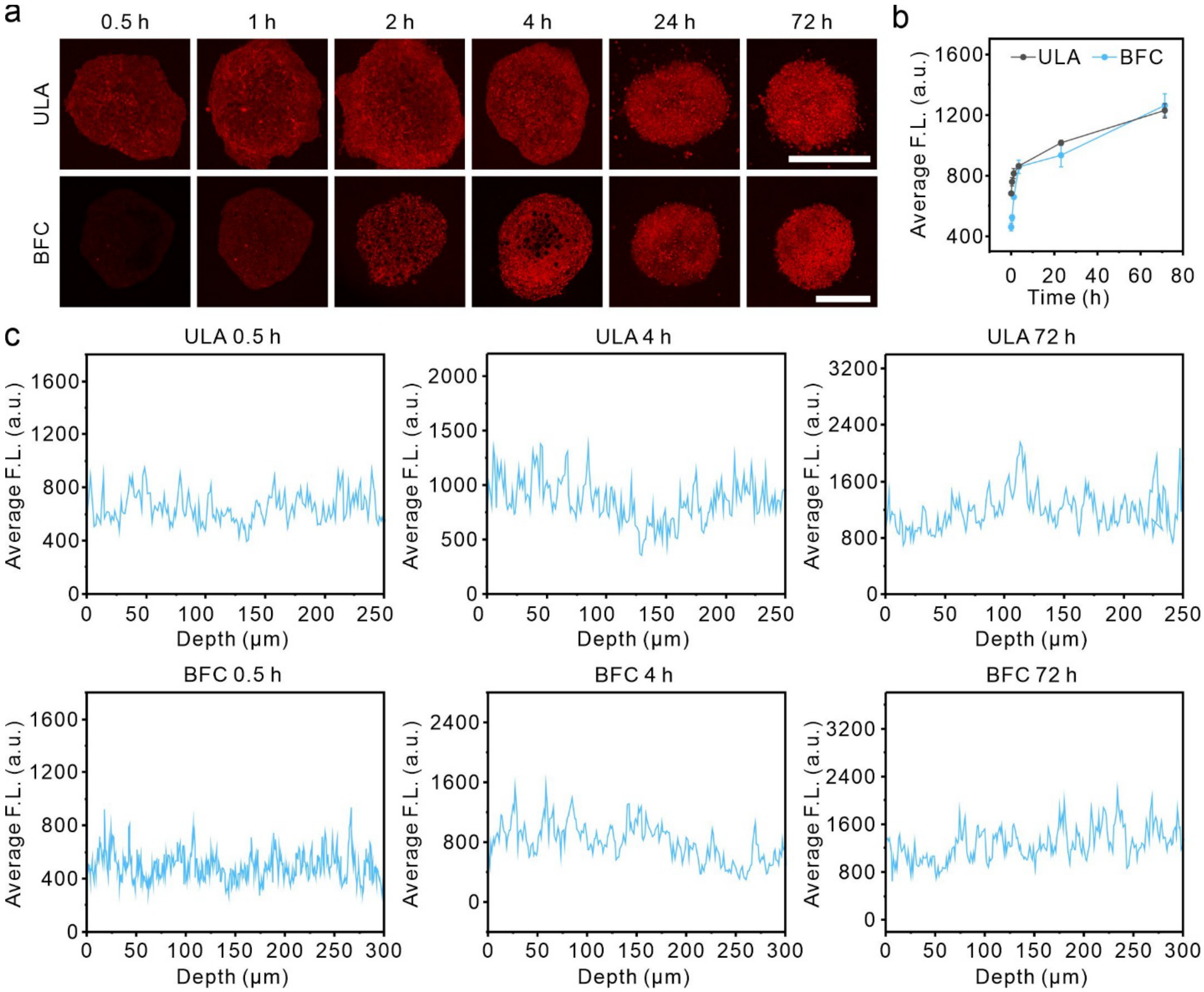

Fig. S35. DOX penetration analysis in ULA and BFC spheroids. (a) Representative fluorescence images showing DOX penetration in ULA and BFC spheroids at 0.5, 1, 2, 4, 24, and 72 h. Scale bars: 400 μm. (b) Time-dependent changes in the average DOX fluorescence intensity of ULA and BFC spheroids. (c) Radial penetration profiles of DOX fluorescence intensity in ULA and BFC spheroids at 0.5, 4, and 72 h.

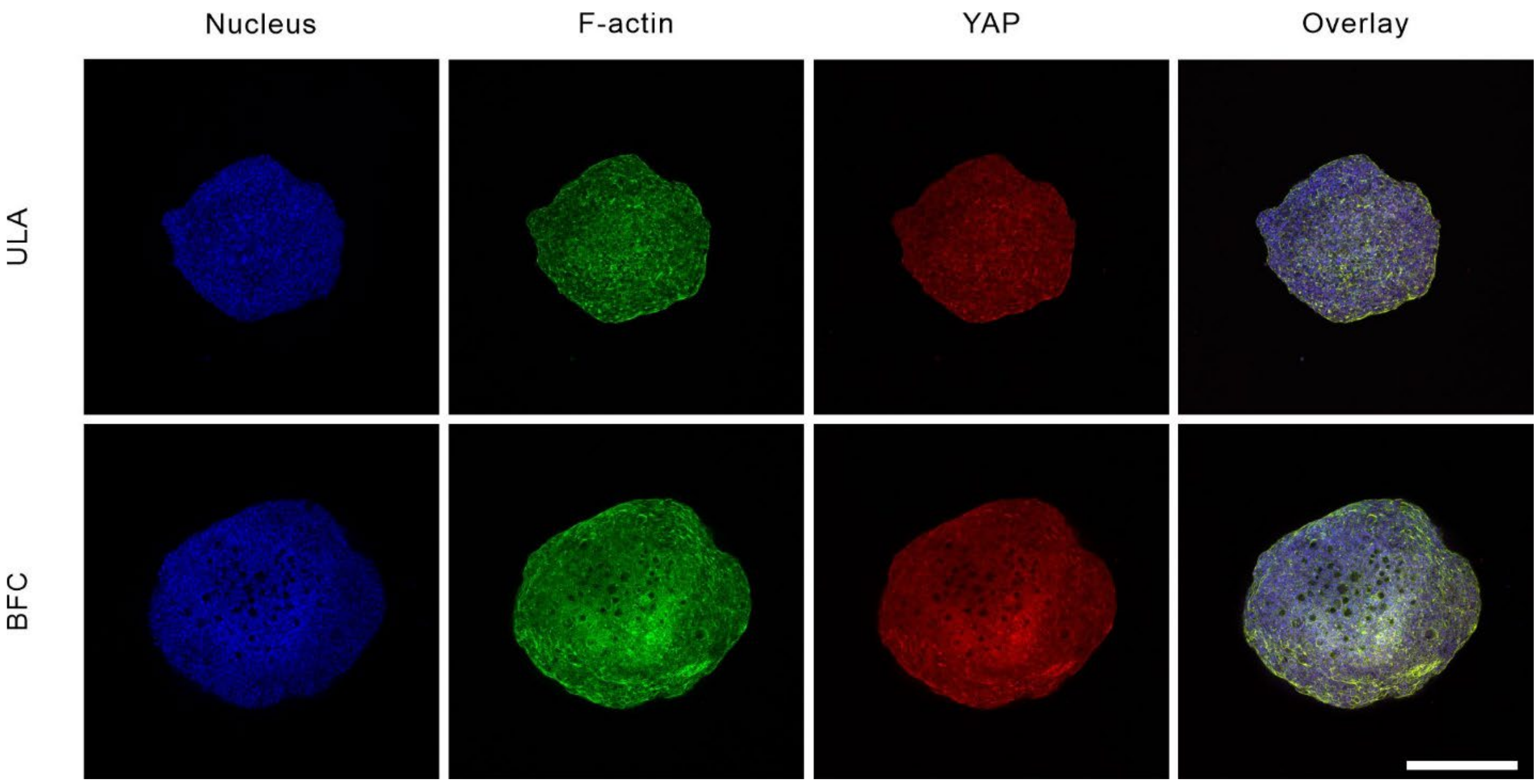

Fig. S36. YAP immunostaining of ULA and BFC spheroids. Representative fluorescence images of ULA and BFC spheroids stained for nuclei, F-actin, and YAP (Cell Signaling Technology, D8H1X, 14074). Scale bars: 400 µm.

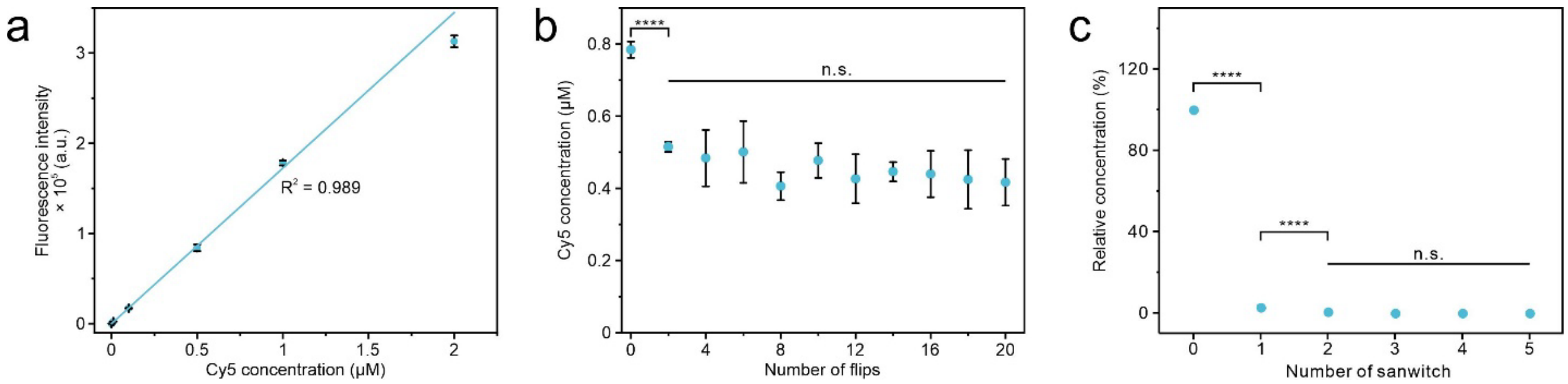

Fig. S37. Quantification of droplet-based dye concentration adjustment and washout in the droplet microarray system. (a) Standard curve of Cy5 concentration versus fluorescence intensity. (b) Changes in Cy5 concentration in droplets after sandwiching at a defined concentration followed by different numbers of flips. (c) Residual relative Cy5 concentration after sequential washout through 1 to 5 rounds of sandwiching with fresh droplets. Data are presented as mean ± SD (n = 5). Statistical analysis was performed using one-way ANOVA followed by Dunnett's multiple-comparisons test; n.s., $P > 0.05$; ****$P \leq 0.0001$.

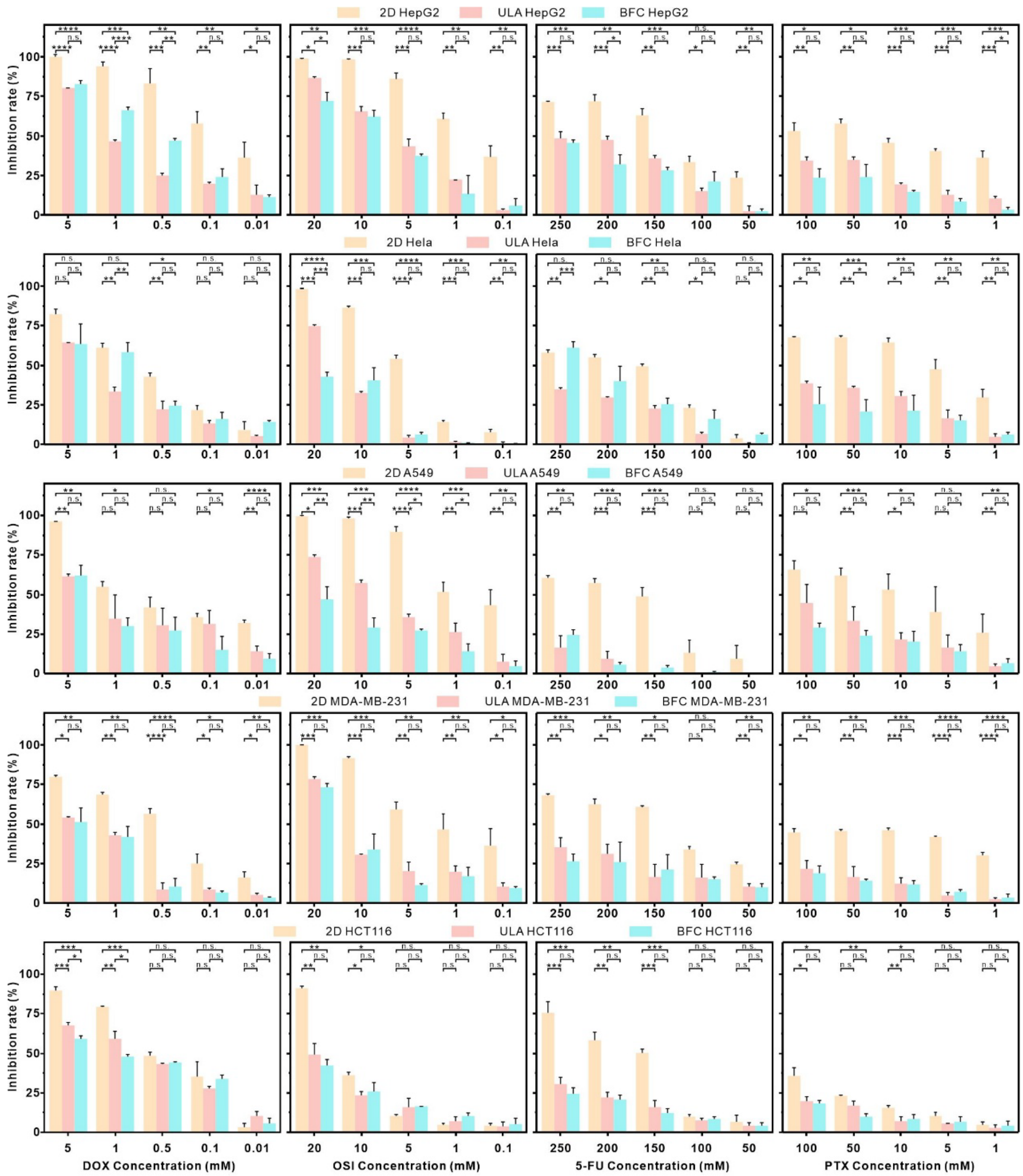

Fig. S38. Drug responses vary among cell types and culture methods. Quantitative analysis of the inhibition rates of HepG2, HeLa, A549, MDA-MB-231, and HCT116 cells cultured using the 2D, ULA, and BFC methods following treatment with the indicated concentrations of doxorubicin (DOX), osimertinib (OSI), 5-fluorouracil (5-FU), and paclitaxel (PTX). The corresponding inhibition-rate heatmap is shown in Fig. 3N. Data are presented as mean ± SD (n = 3). Statistical significance was assessed using two-way ANOVA followed by Šídák's multiple-comparisons test for comparisons among culture methods at each drug concentration. n.s., $P > 0.05$; $^{*}P \leq 0.05$; $^{**}P \leq 0.01$; $^{***}P \leq 0.001$; $^{****}P \leq 0.0001$.

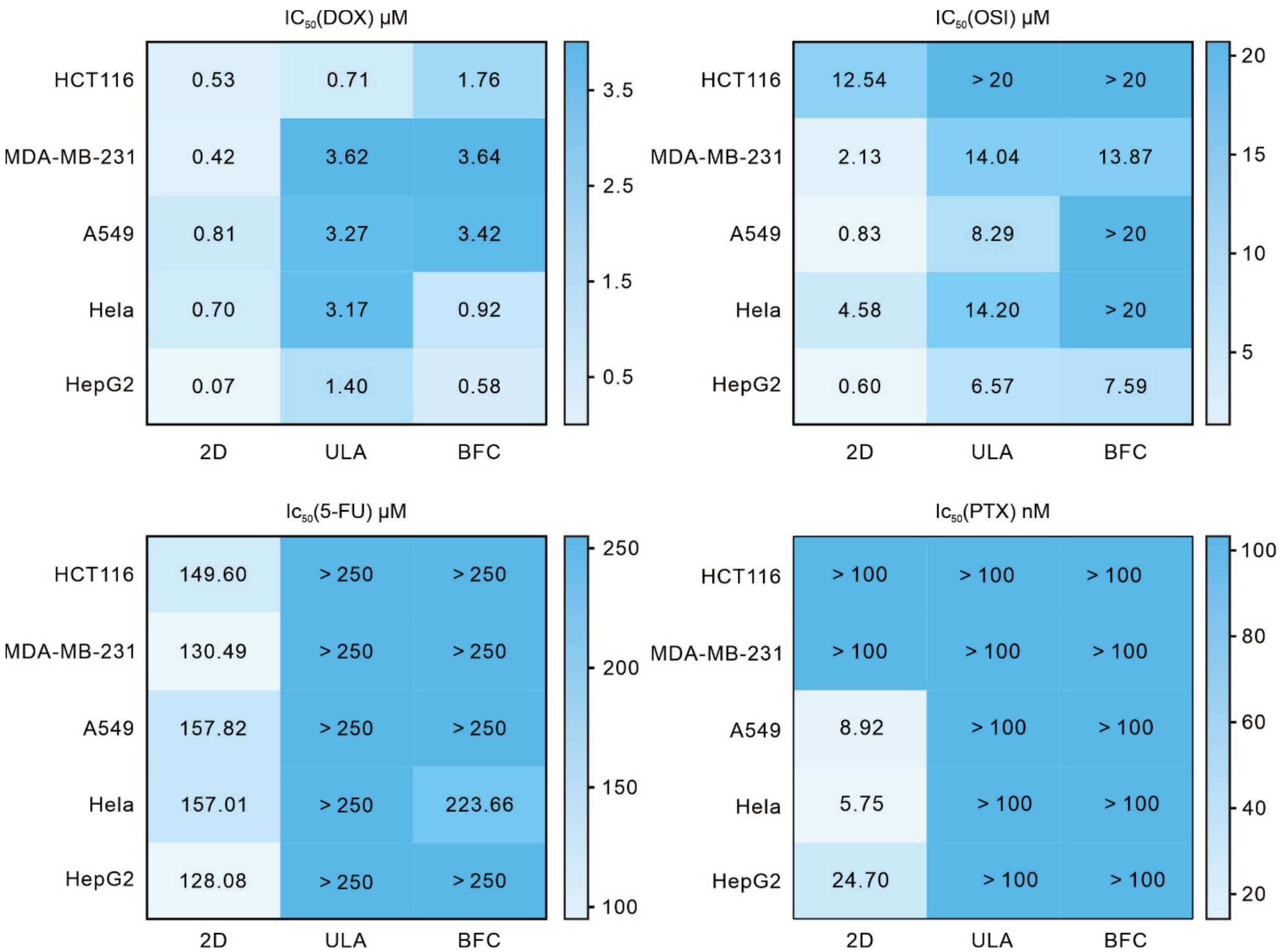

Fig. S39. IC50 profiles reveal culture-dependent drug sensitivity across multiple cell types. Heatmap of the IC50 values of DOX, OSI, 5-FU, and PTX for HepG2, HeLa, A549, MDA-MB-231, and HCT116 cells cultured using the 2D, ULA, and BFC methods. IC50 values were calculated using linear regression or logistic fitting.

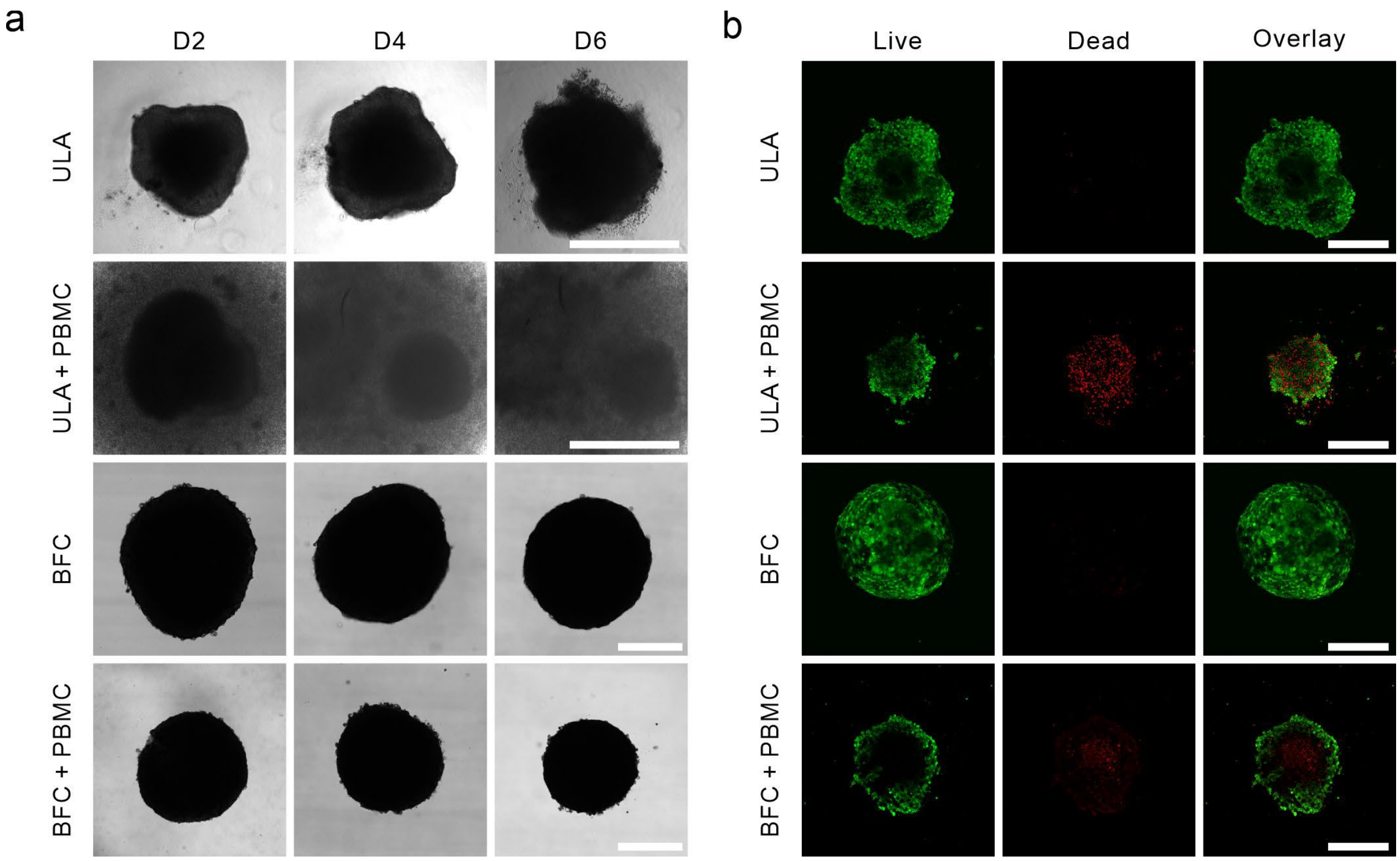

Fig. S40. **Co-culture of HCT116 spheroids with PBMCs.** (a) Representative bright-field images of HCT116 spheroids cultured under ULA, ULA + PBMC, BFC, and BFC + PBMC conditions at D2, D4, and D6. Scale bars: 400 μm. (b) Representative Live/Dead staining images of HCT116 spheroids under ULA, ULA + PBMC, BFC, and BFC + PBMC conditions after 6 days of culture. Live cells were stained with Calcein-AM (green), and dead cells were stained with PI (red). Scale bars: 400 μm.

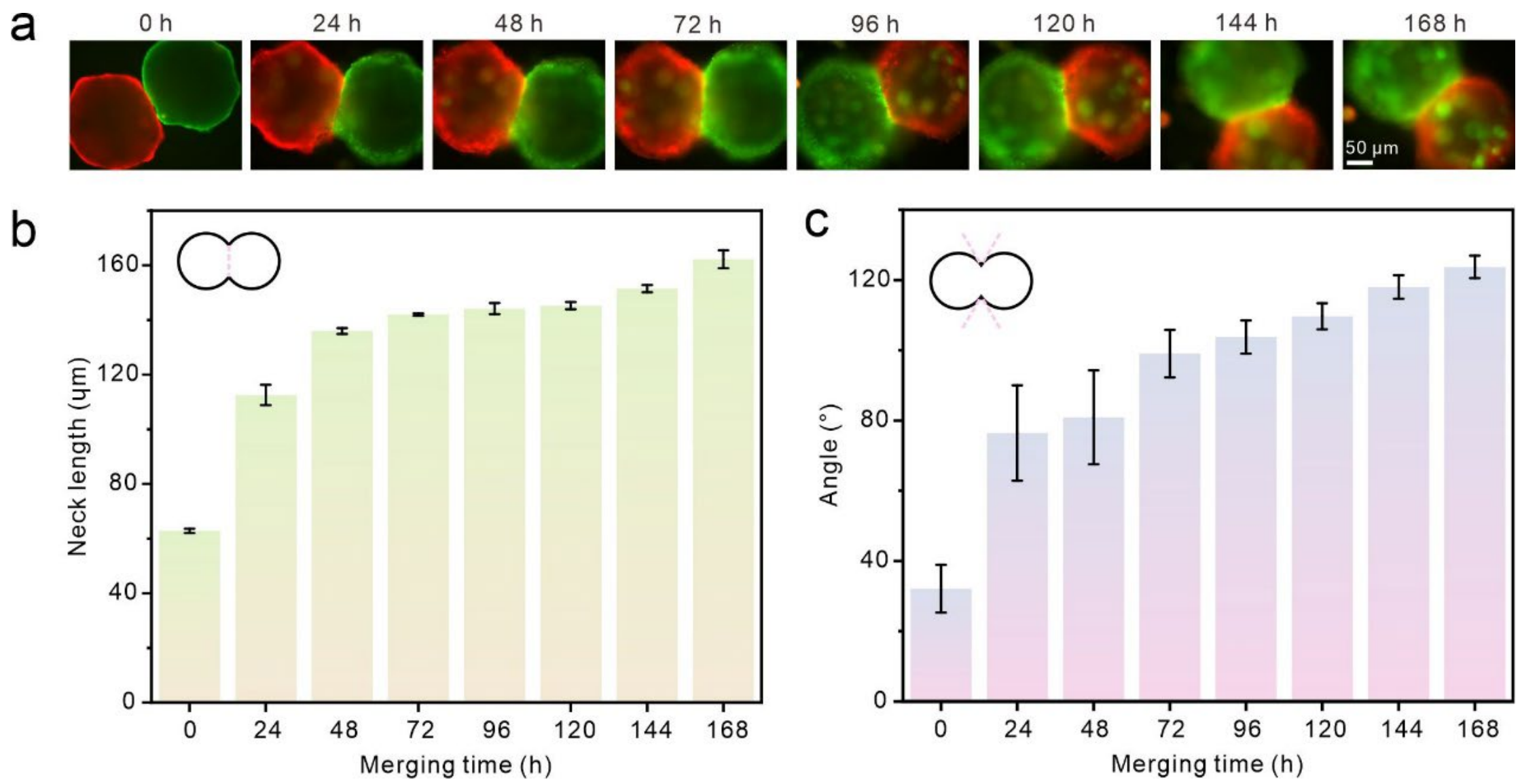

Fig. S41. Fusion dynamics of ULA-cultured spheroids in ULA plates. (a) Representative fluorescence microscopy images showing the fusion process of two ULA-cultured spheroids in ULA plates from D1 to D7. (b, c) Time-dependent changes in neck length (L_{neck}) (b) and fusion angle θ (c) during ULA spheroid fusion. Data are presented as mean ± SD (n = 5).

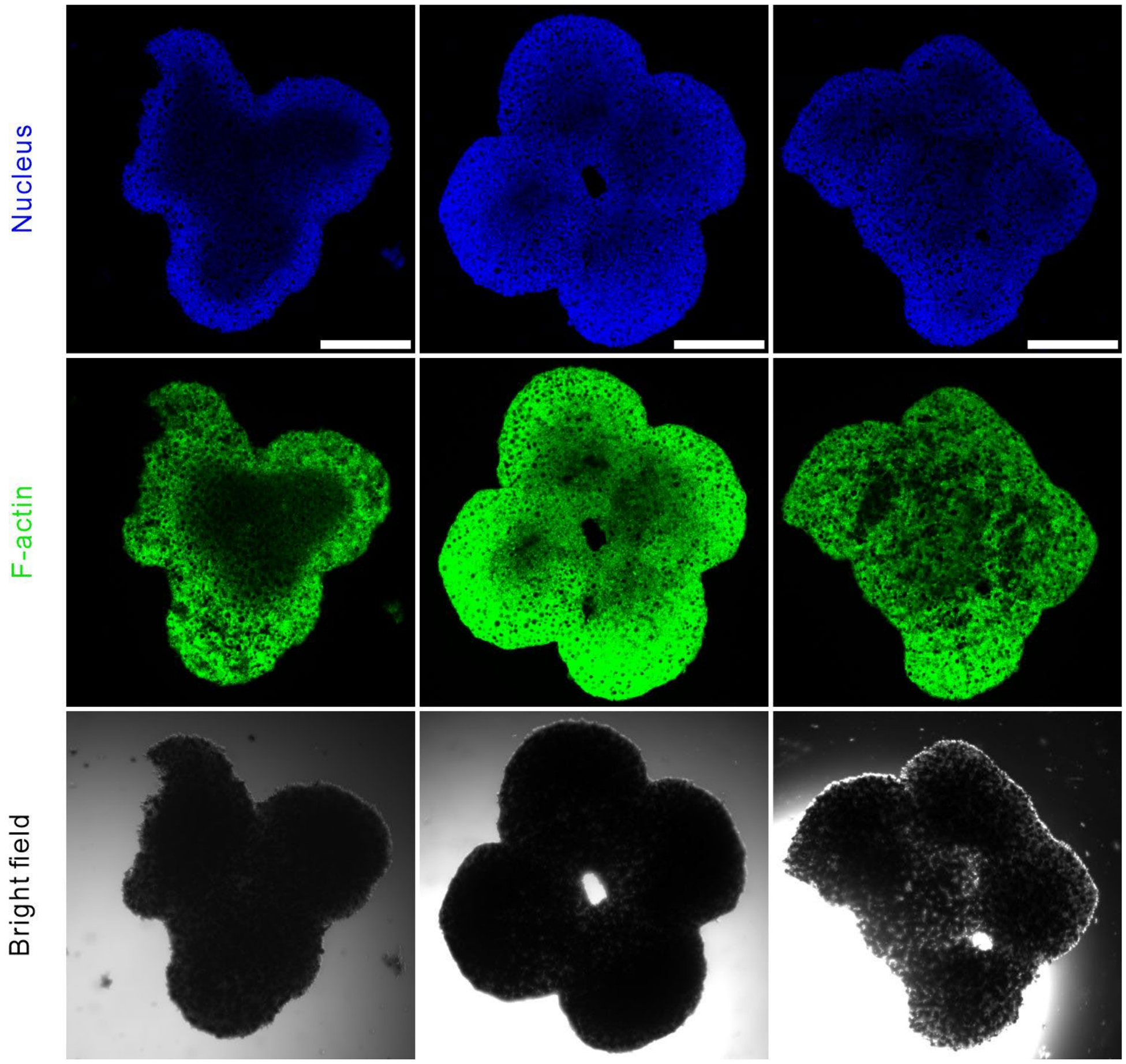

Fig. S42. F-actin staining reveals the cytoskeletal organization of multicellular spheroids. Representative fluorescence images of multicellular spheroids stained with FITC-phalloidin for F-actin and Hoechst for nuclei. Scale bars, 500 μm.

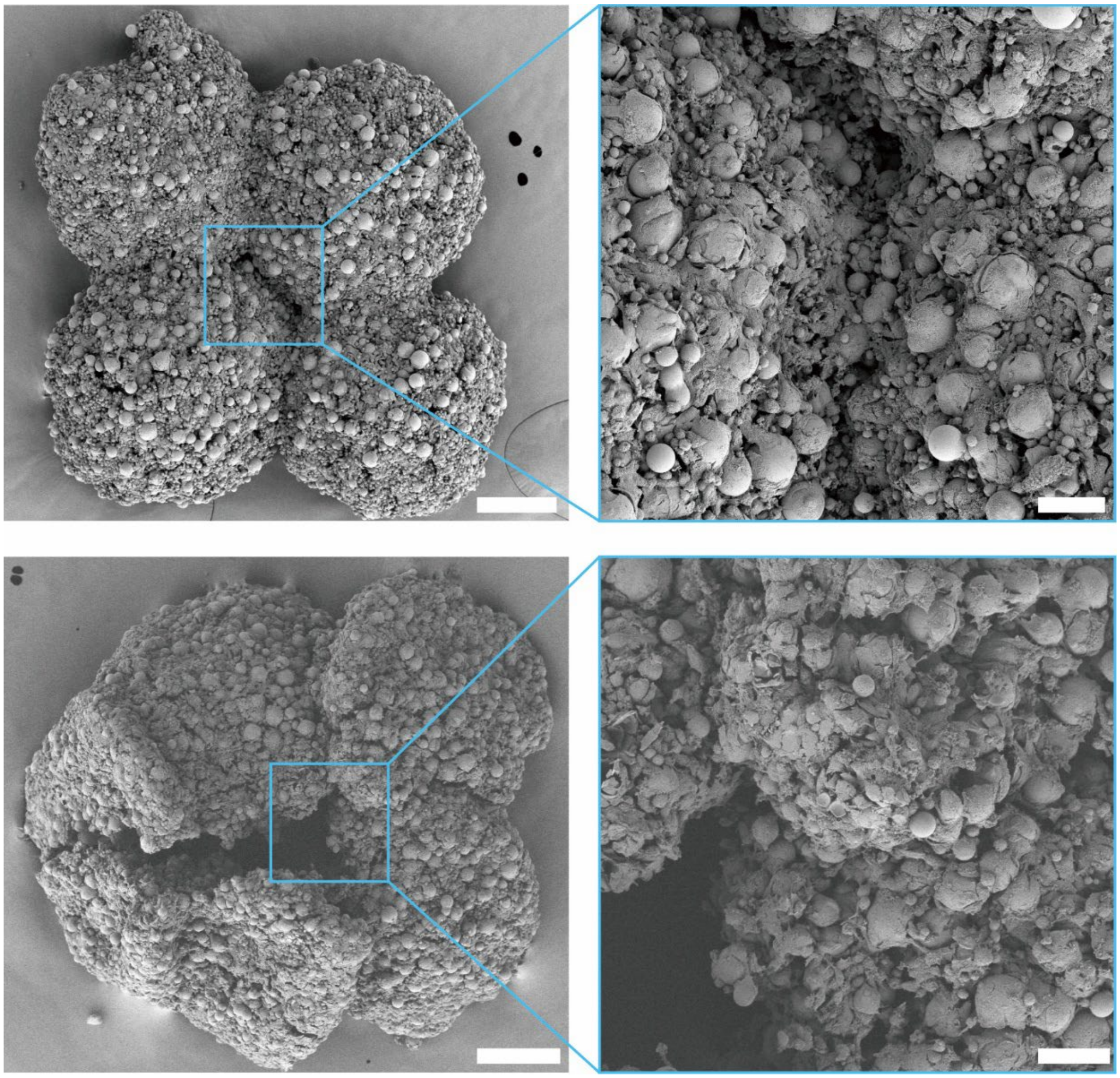

Fig. S43. SEM imaging reveals the surface architecture of multicellular spheroids. Representative SEM images of multicellular spheroids and enlarged views of their surface morphology. Scale bars, 200 µm; enlarged views, 50 µm.

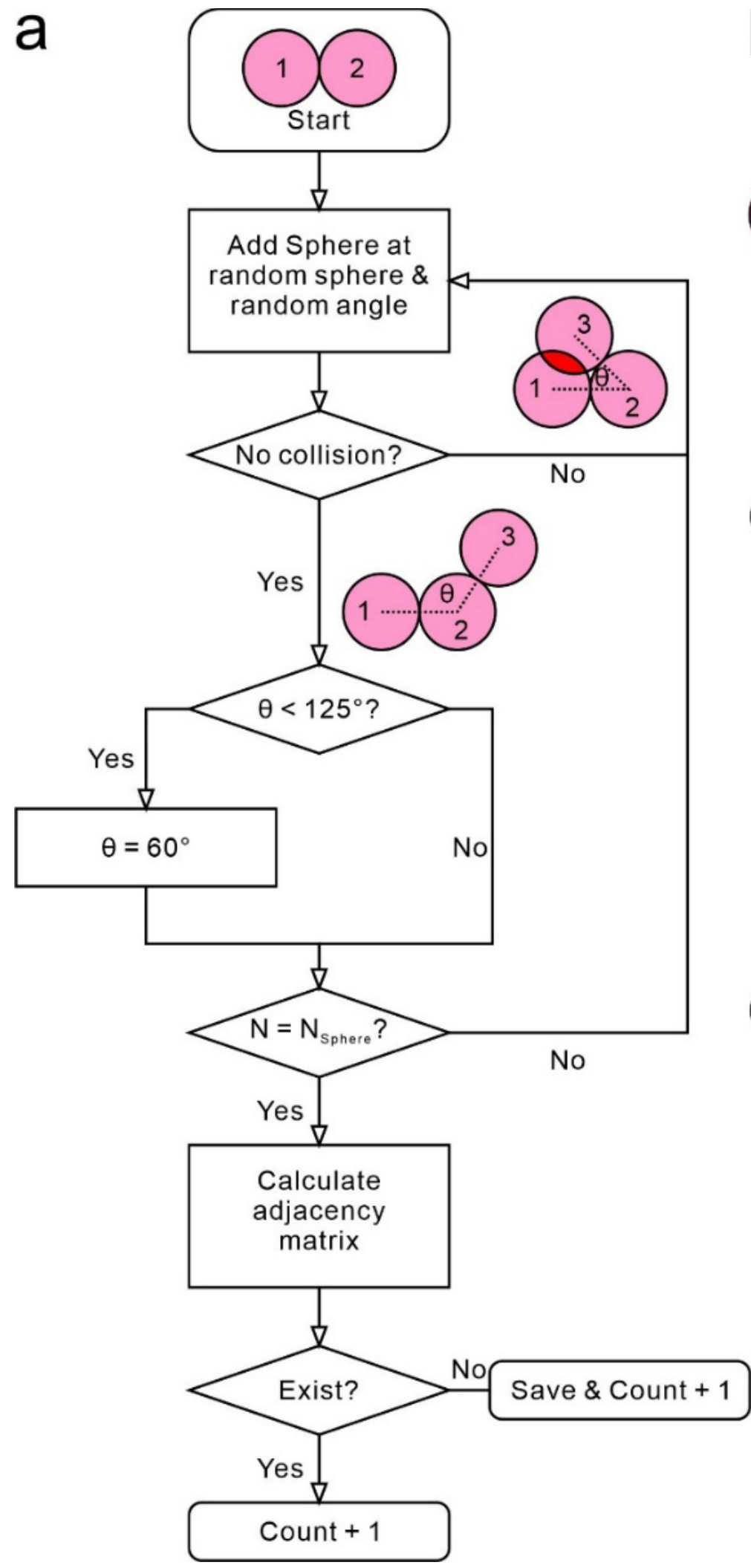

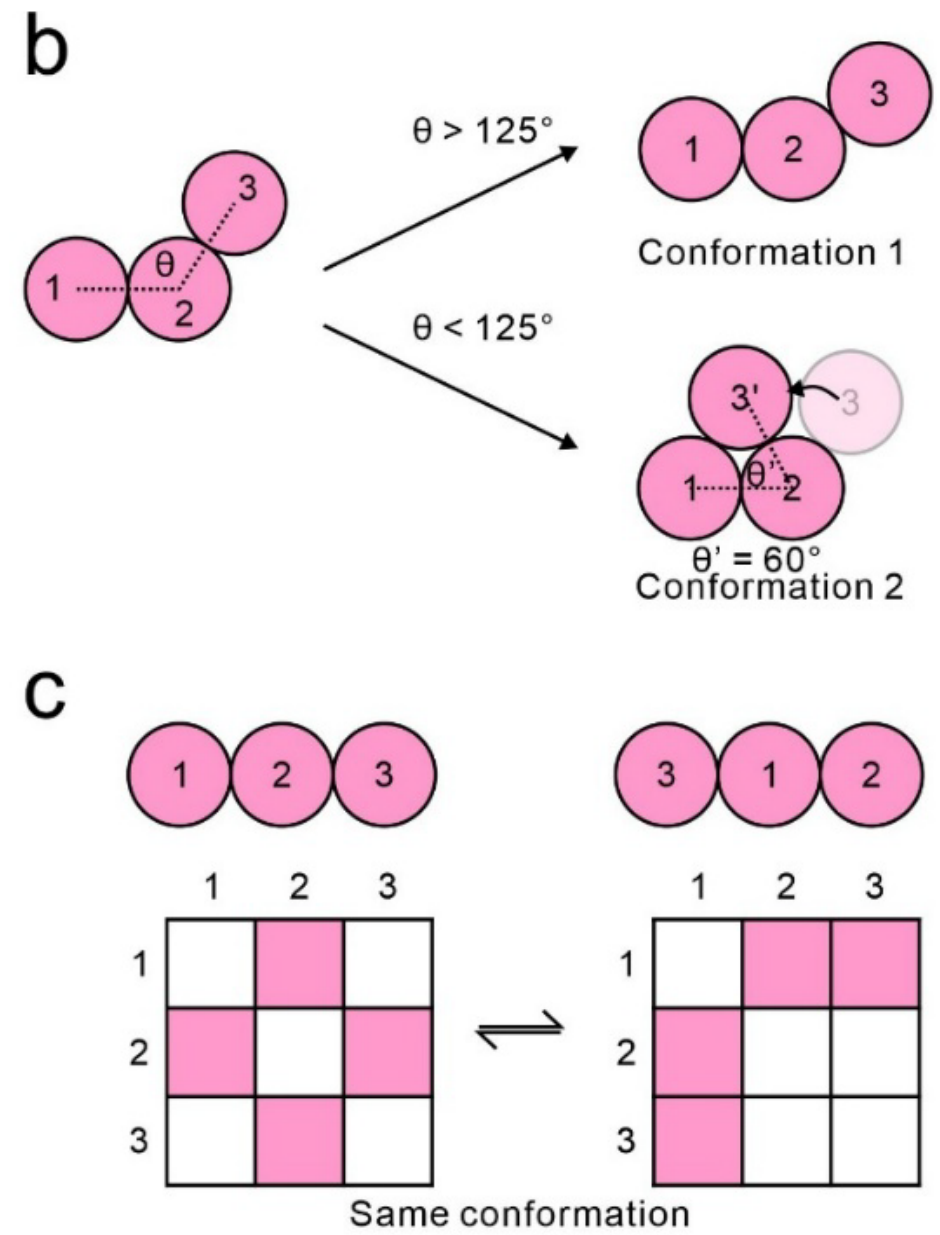

d

N_{sphere}	$N_{conformations}$
2	1 (N = 10000)
3	2 (N = 10000)
4	4 (N = 10000)
5	11 (N = 20000)
6	37 (N = 40000)
7	131 (N = 80000)

Fig. S44. Computational modeling of spheroid fusion probability. (a) Computational modeling of BFC spheroid fusion probability. The method for calculating the merging probability of BFC cell spheroids was adapted from Levkin et al. (Advanced Materials. 2021, 33 (4), 2006434.) and was modified. (b) Since cell spheroids float only at the liquid surface and assemble exclusively in 2D, we constructed the algorithm based on a two-dimensional model. First, the collision scenario of two cell spheroids was modeled in a 2D plane. A third spheroid was then randomly added near an existing spheroid at an angle θ. Subsequently, it was determined whether the added spheroid collided with surrounding spheroids; if collision occurred, a new spheroid was regenerated. Due to the buoyancy effect, a correction angle was introduced. (c) Combining with actual ratios, when the angle between the added spheroid and its two nearest spheroids was less than 125°, the spheroid would move closer to the nearest spheroid (i.e., the angle becomes 60°). This process was repeated until a sufficient number of merged spheroids was achieved. Finally, the adjacency matrix was computed and compared against existing matrices to determine the presence of isomorphic graphs. (d) If an identical matrix existed, its count was increased by one; if not, the new matrix was saved and its count was set to one. This ultimately yielded the number of conformations and their probabilities generated by merging different numbers of cell spheroids.

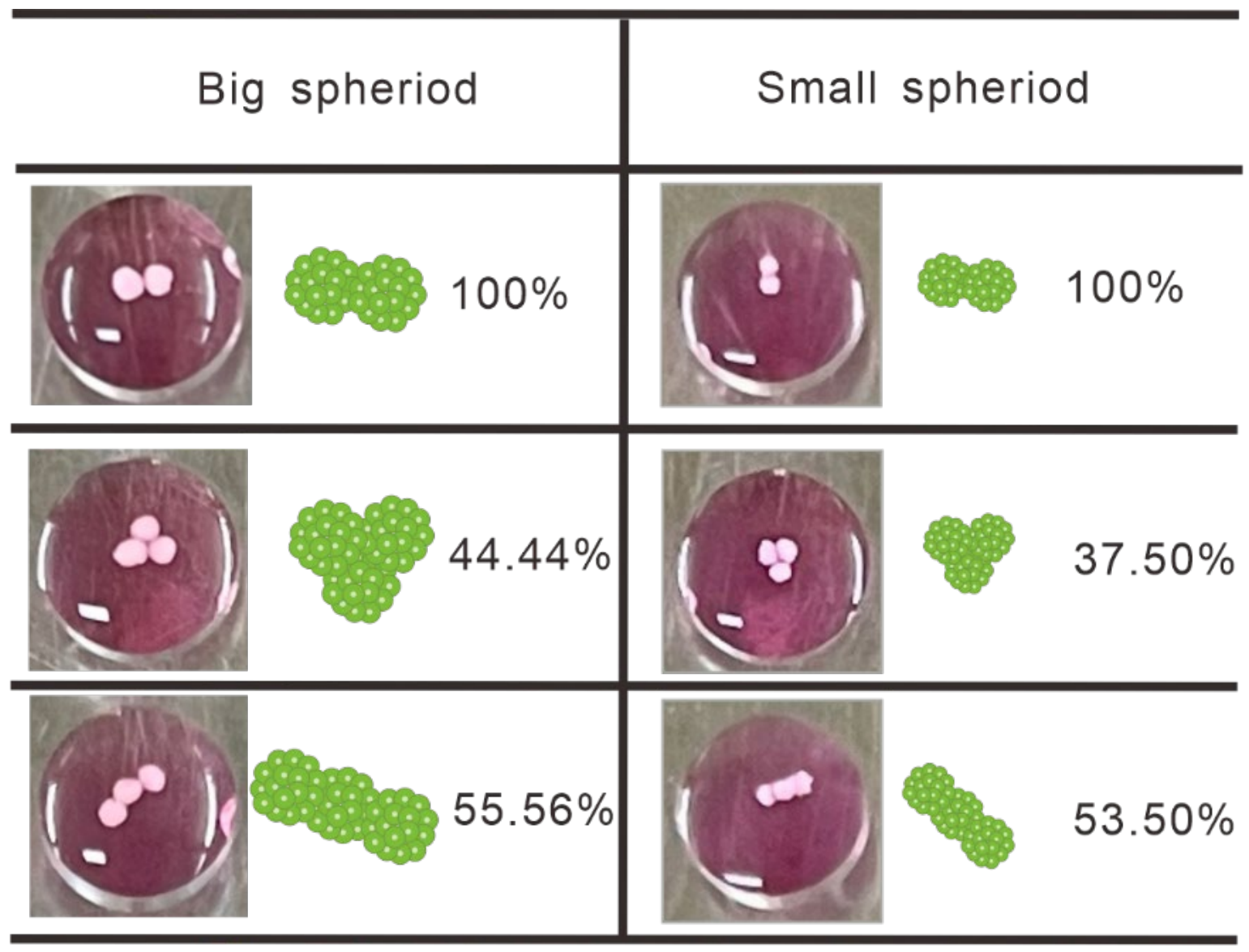

Fig. S45. Spheroid size influences the outcome of sandwich-mediated assembly. Comparison of the probabilities of forming different assembly structures through sandwich fusion of cell spheroids of varying sizes on substrates of the same size.

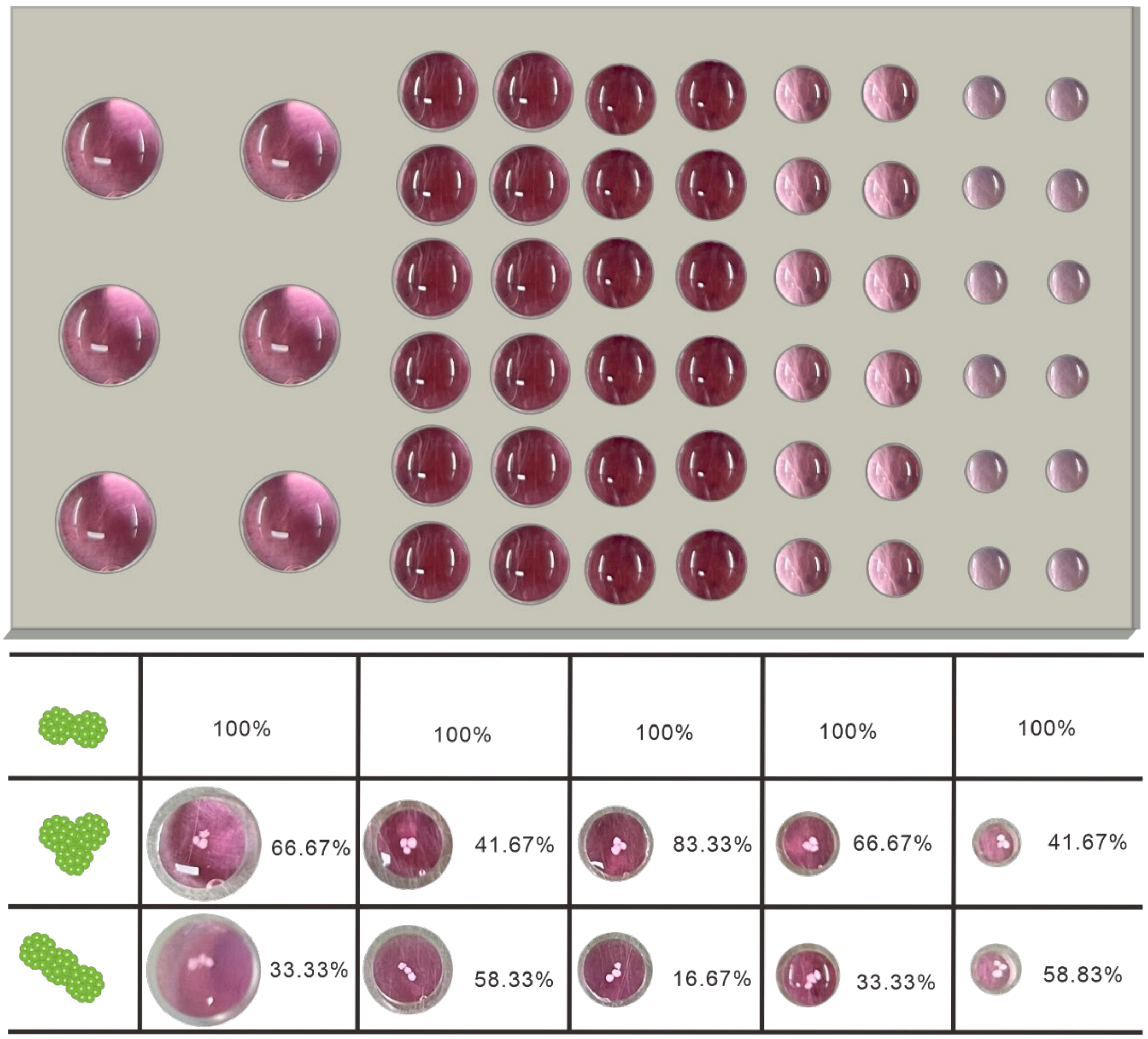

Fig. S46. Substrate size influences the assembly of identically sized spheroids. Comparative analysis of the probabilities of forming distinct assembly structures through sandwich fusion of identically sized cell spheroids on circular substrates with diameters of 3, 4, 5, 6, and 7 mm.

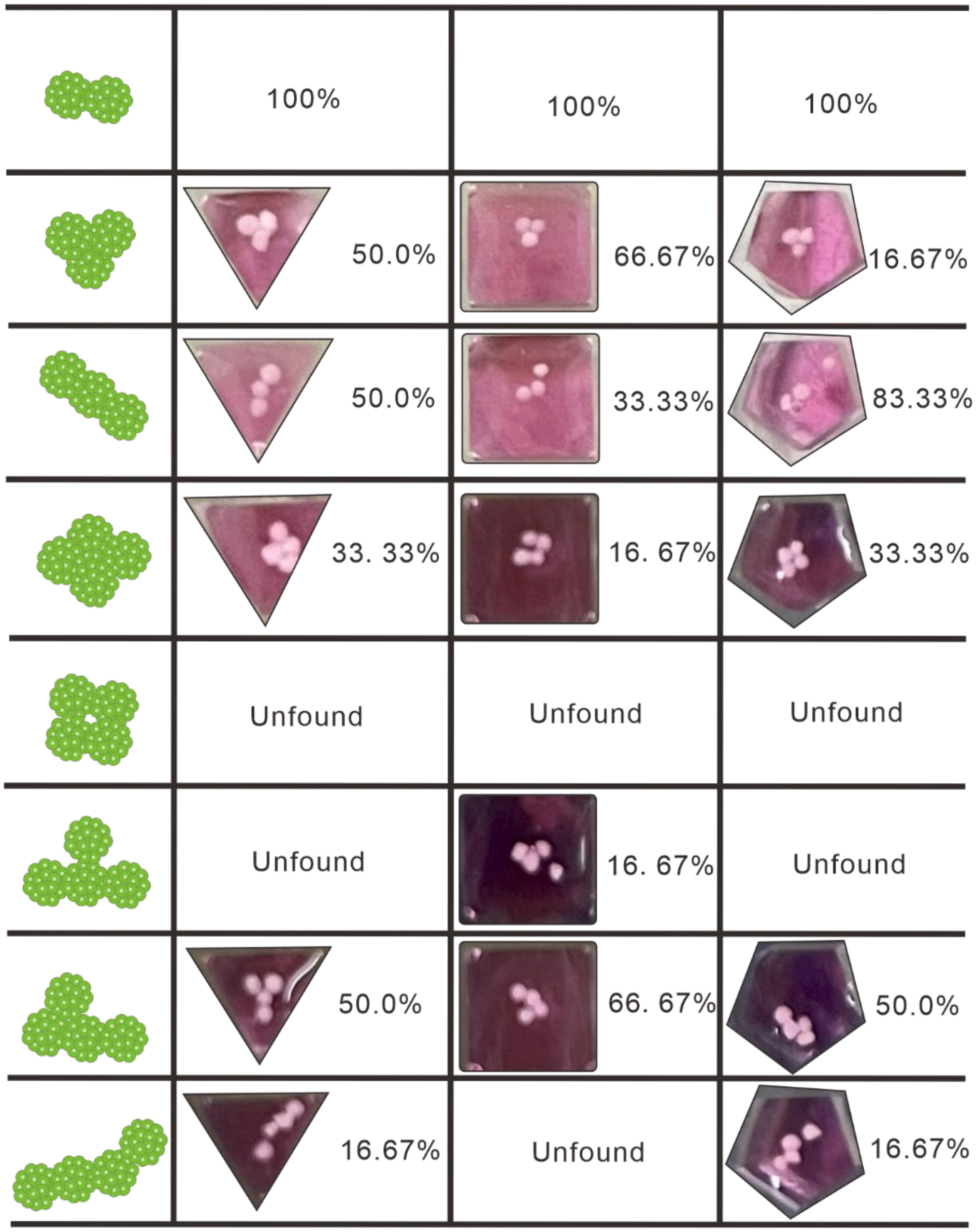

Fig. S47. Substrate geometry influences the assembly of identically sized spheroids. Comparative analysis of the probabilities of forming distinct assembly structures through sandwich fusion of identically sized cell spheroids cultured on substrates with different geometries.

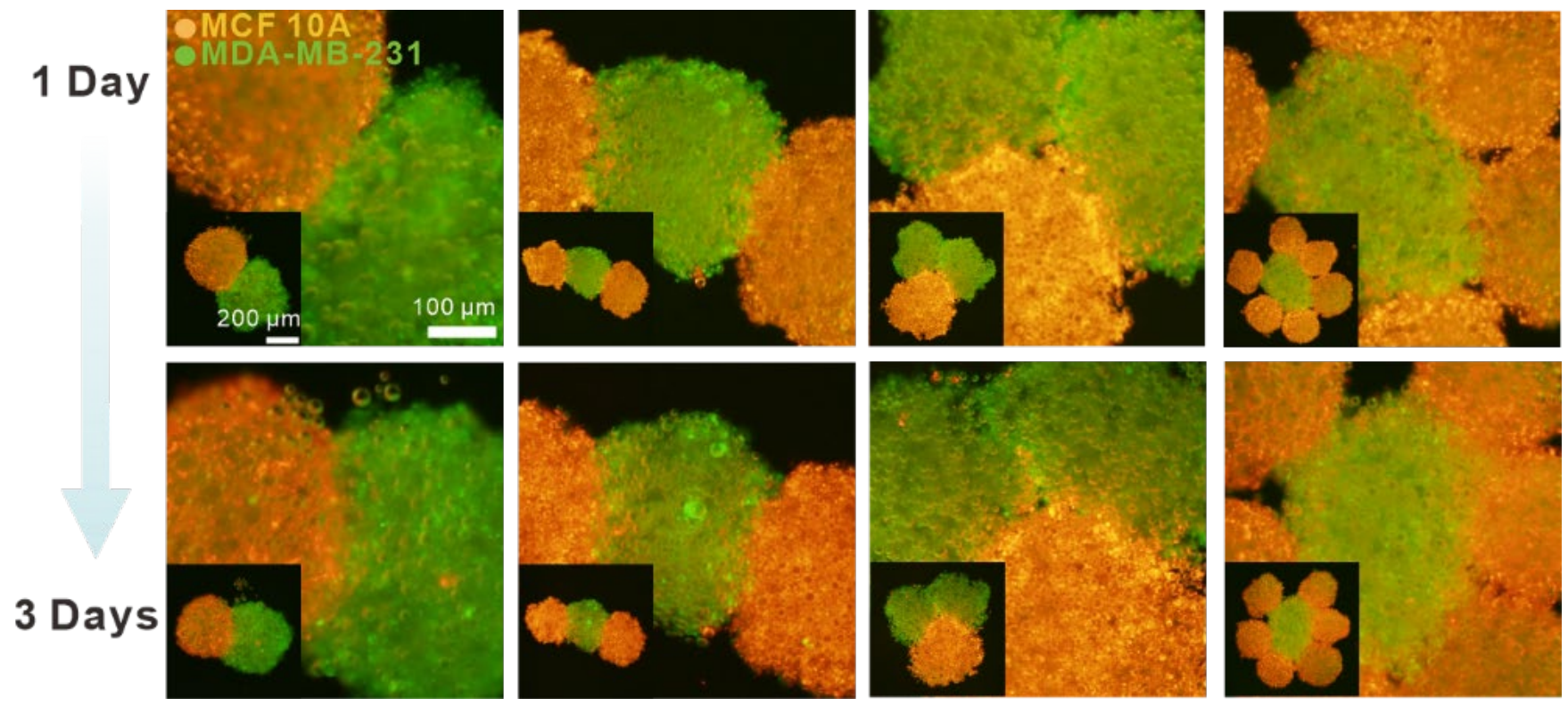

Fig. S48. Programmable assembly enables progressive fusion of heterotypic spheroids. Magnified images of the boundary between MCF-10A and MDA-MB-231 spheroids after programmable heterotypic assembly (top) and during fusion over 3 days (bottom).

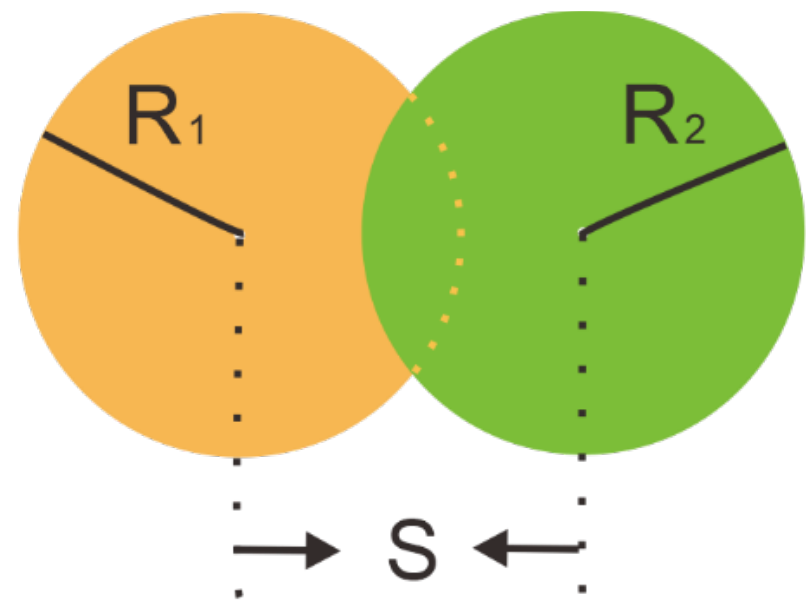

Fig. S49. Fusion index enables quantitative evaluation of spheroid fusion. Schematic illustration of the method used to calculate the fusion index, also referred to as the fusion degree (FD).

Table S1. Benchmark comparison of BFC with representative spheroid culture methods.

Culture method	Scaffold	External field / equipment	Single-spheroid formation	High-throughput transfer / solution exchange	Structure programming	Cost / reusability
**BFC**[this work]	**CGB**	**None**	**Yes**	**Parallel sandwich transfer; ~1–10 s**	**Self-driven horizontal/vertical assembly**	**Low equipment burden; reusable non-contact chip; consumable CGBs**
Hanging drop[5, 27]	None	None	Yes	Parallel droplet transfer; ~1-10 min	2D droplet merging	Low cost; disposable plates/arrays; higher cost with automation
ULA[21, 52]	None	None; centrifugation optional	Yes	Manual serial exchange or robotic handling	Limited	Low–moderate cost; disposable ULA plates
Bioreactor[53]	None	Stirred bioreactor	No; bulk aggregates	Bulk solution exchange	Limited	High cost; specialized vessel and control system
Magnetic assisted levitation[17, 54]	MNPs*	External magnetic field	Platform-dependent	Magnet-guided manipulation; limited parallelization	Magnet-defined geometry	Moderate–high cost; magnetic labeling and field setup
Matrigel[8]	Matrix	Temperature-controlled gelation	No; matrix-embedded culture	Manual serial exchange	Matrix extrusion/printing	Moderate cost; single-use matrix; batch variability

* MNPs, magnetic nanoparticles.

Movie S1.
Cell spheroid transfer from the bottom droplet to the top droplet upon droplet sandwich merging.

Movie S2.
High-throughput cell spheroid transfer.

REFERENCES

1. Z. Zhao, X. Chen, A. M. Dowbaj, A. Sljukic, K. Bratlie, L. Lin, E. L. S. Fong, G. M. Balachander, Z. Chen, A. Soragni, M. Huch, Y. A. Zeng, Q. Wang, H. Yu, Organoids. *Nat. Rev. Methods Primers* **2**, 94 (2022).

2. M. Hofer, M. P. Lutolf, Engineering organoids. *Nat. Rev. Mater.* **6**, 402–420 (2021).

3. P. R. Baraniak, T. C. McDevitt, Scaffold-free culture of mesenchymal stem cell spheroids in suspension preserves multilineage potential. *Cell Tissue Res.* **347**, 701–711 (2012).

4. F. Madoux, A. Tanner, M. Vessels, L. Willetts, S. Hou, L. Scampavia, T. P. Spicer, A 1536-well 3d viability assay to assess the cytotoxic effect of drugs on spheroids. *SLAS Discov.* **22**, 516–524 (2017).

5. H. Cui, X. Wang, J. Wesslowski, T. Tronser, J. Rosenbauer, A. Schug, G. Davidson, A. A. Popova, P. A. Levkin, Assembly of multi-spheroid cellular architectures by programmable droplet merging. *Adv. Mater.* **33**, e2006434 (2021).

6. H. Shan, M. Chen, S. Zhao, X. Wei, M. Zheng, Y. Li, Q. Lin, Z. Jiang, Z. Chen, C. Fei, Z. Li, Z. Chen, X. Chen, Acoustic virtual 3D scaffold for direct-interacting tumor organoid–immune cell coculture systems. *Sci. Adv.* **10**, eadr4831 (2024).

7. M. Diba, S. Spaans, S. I. S. Hendrikse, M. M. C. Bastings, M. J. G. Schotman, J. F. van Sprang, D. J. Wu, F. J. M. Hoeben, H. M. Janssen, P. Y. W. Dankers, Engineering the dynamics of cell adhesion cues in supramolecular hydrogels for facile control over cell encapsulation and behavior. *Adv. Mater.* **33**, e2008111 (2021).

8. Y.-H. Peng, S.-K. Hsiao, K. Gupta, A. Ruland, G. K. Auernhammer, M. F. Maitz, S. Boye, J. Lattner, C. Gerri, A. Honigmann, C. Werner, E. Krieg, Dynamic matrices with DNA-encoded viscoelasticity for cell and organoid culture. *Nat. Nanotechnol.* **18**, 1463–1473 (2023).

9. X. Yang, B. Yang, Y. Deng, X. Xie, Y. Qi, G. Yan, X. Peng, P. Zhao, L. Bian, Coacervation-mediated cytocompatible formation of supramolecular hydrogels with self-evolving macropores for 3D multicellular spheroid culture. *Adv. Mater.* **35**, e2300636 (2023).

10. J. Wei, Y. Sun, H. Wang, T. Zhu, L. Li, Y. Zhou, Q. Liu, Z. Dai, W. Li, T. Yang, B. Wang, C. Zhu, X. Shen, Q. Yao, G. Song, Y. Zhao, H. Pei, Designer cellular spheroids with DNA origami for drug screening. *Sci. Adv.* **10**, eado9880 (2024).

11. L. Zwi-Dantsis, B. Wang, C. Marijon, S. Zonetti, A. Ferrini, L. Massi, D. J. Stuckey, C. M. Terracciano, M. M. Stevens, Remote magnetic nanoparticle manipulation enables the dynamic patterning of cardiac tissues. *Adv. Mater.* **32**, e1904598 (2020).

12. A. Leferink, D. Schipper, E. Arts, E. Vrij, N. Rivron, M. Karperien, K. Mittmann, C. van Blitterswijk, L. Moroni, R. Truckenmüller, Engineered micro-objects as scaffolding elements in cellular building blocks for bottom-up tissue engineering approaches. *Adv. Mater.* **26**, 2592–2599 (2014).

13. T. H. Qazi, J. Wu, V. G. Muir, S. Weintraub, S. E. Gullbrand, D. Lee, D. Issadore, J. A. Burdick, Anisotropic rod-shaped particles influence injectable granular hydrogel properties and cell invasion. *Adv. Mater.* **34**, e2109194 (2022).

14. N. D. Caprio, J. A. Burdick, Engineered biomaterials to guide spheroid formation, function, and fabrication into 3D tissue constructs. *Acta Biomater.* **165**, 4–18 (2023).

15. A. Elosegui-Artola, A. Gupta, A. J. Najibi, B. R. Seo, R. Garry, C. M. Tringides, I. de Lázaro, M. Darnell, W. Gu, Q. Zhou, D. A. Weitz, L. Mahadevan, D. J. Mooney, Matrix viscoelasticity controls spatiotemporal tissue organization. *Nat. Mater.* **22**, 117–127 (2023).

16. H. Haag, P. D. Dalton, V. Bloemen, The synergy of biomimetic design strategies for tissue constructs. *Adv. Funct. Mater.* **32**, 2201414 (2022).

17. G. R. Souza, J. R. Molina, R. M. Raphael, M. G. Ozawa, D. J. Stark, C. S. Levin, L. F. Bronk, J. S. Ananta, J. Mandelin, M.-M. Georgescu, J. A. Bankson, J. G. Gelovani, T. C. Killian, W. Arap, R. Pasqualini, Three-dimensional tissue culture based on magnetic cell levitation. *Nat. Nanotechnol.* **5**, 291–296 (2010).

18. W. L. Haisler, D. M. Timm, J. A. Gage, H. Tseng, T. C. Killian, G. R. Souza, Three-dimensional cell culturing by magnetic levitation. *Nat. Protoc.* **8**, 1940–1949 (2013).

19. A. Tocchio, N. G. Durmus, K. Sridhar, V. Mani, B. Coskun, R. El Assal, U. Demirci, Magnetically guided self-assembly and coding of 3D living architectures. *Adv. Mater.* **30**, 1705034 (2018).

20. J. G. Roth, L. G. Brunel, M. S. Huang, Y. Liu, B. Cai, S. Sinha, F. Yang, S. P. Pașca, S. Shin, S. C. Heilshorn, Spatially controlled construction of assembloids using bioprinting. *Nat. Commun.* **14**, 4346 (2023).

21. N. Brandenberg, S. Hoehnel, F. Kuttler, K. Homicsko, C. Ceroni, T. Ringel, N. Gjorevski, G. Schwank, G. Coukos, G. Turcatti, M. P. Lutolf, High-throughput automated organoid culture via stem-cell aggregation in microcavity arrays. *Nat. Biomed. Eng.* **4**, 863–874 (2020).

22. F. Birey, J. Andersen, C. D. Makinson, S. Islam, W. Wei, N. Huber, H. C. Fan, K. R. C. Metzler, G. Panagiotakos, N. Thom, N. A. O'Rourke, L. M. Steinmetz, J. A. Bernstein, J. Hallmayer, J. R. Huguenard, S. P. Paşca, Assembly of functionally integrated human forebrain spheroids. *Nature* **545**, 54–59 (2017).

23. N. Vogt, Assembloids. *Nat. Methods* **18**, 27–27 (2021).

24. J. Andersen, O. Revah, Y. Miura, N. Thom, N. D. Amin, K. W. Kelley, M. Singh, X. Chen, M. V. Thete, E. M. Walczak, H. Vogel, H. C. Fan, S. P. Paşca, Generation of functional human 3D cortico-motor assembloids. *Cell* **183**, 1913–1929.e26 (2020).

25. A. A. Popova, S. M. Schillo, K. Demir, E. Ueda, A. Nesterov-Mueller, P. A. Levkin, Droplet-Array (DA) sandwich chip: a versatile platform for high-throughput cell screening based on superhydrophobic–superhydrophilic micropatterning. *Adv. Mater.* **27**, 5217–5222 (2015).

26. W. Feng, E. Ueda, P. A. Levkin, Droplet microarrays: from surface patterning to high-throughput applications. *Adv. Mater.* **30**, e1706111 (2018).

27. J. E. Urrutia Gómez, M. Zhou, N. K. Mandsberg, J. A. Serna, J. von Padberg, S. Liu, M. Reischl, P. A. Levkin, A. A. Popova, Highly parallel and high-throughput

nanoliter-scale liquid, cell, and spheroid manipulation on droplet microarray. *Adv. Funct. Mater.* **35**, 2410355 (2025).

28. Y. Xiang, H. Zhang, H. Lu, B. Wei, C. Su, X. Qin, M. Fang, X. Li, F. Yang, Bioorthogonal microbubbles with antifouling nanofilm for instant and suspended enrichment of circulating tumor cells. *ACS Nano* **17**, 9633–9646 (2023).

29. B. Ayan, D. N. Heo, Z. Zhang, M. Dey, A. Povilianskas, C. Drapaca, I. T. Ozbolat, Aspiration-assisted bioprinting for precise positioning of biologics. *Sci. Adv.* **6**, eaaw5111 (2020).

30. X. Qin, Y. Xiang, L. Mao, Y. Yang, B. Wei, H. Lu, X. Li, Y. Zhang, F. Yang, Buoyant metal–organic framework corona-driven fast isolation and ultrasensitive profiling of circulating extracellular vesicles. *ACS Nano* **18**, 14569–14582 (2024).

31. B. Wei, Y. Xiang, X. Qin, Y. Yang, H. Lu, H. Li, M. Fang, X. Li, F. Yang, EVs-on-a-Bubble: self-aggregated click bubbles streamline isolation and amplified profiling of circulating extracellular vesicles. *Adv. Funct. Mater.* **34**, 2310823 (2024).

32. Y. Zhang, X. Qin, Z. Xu, W. Liu, H. Lu, Y. Yang, J. Yang, X. Li, Y. Zhang, F. Yang, Electric field-resistant bubble-enhanced wash-free profiling of extracellular vesicle surface markers. *ACS Nano* **19**, 8093–8107 (2025).

33. A. F. Cunha, A. F. V. Matias, C. S. Dias, M. B. Oliveira, N. A. M. Araújo, J. F. Mano, Cell response in free-packed granular systems. *ACS Appl. Mater. Interfaces* **14**, 40469–40480 (2022).

34. B. S. Jacobson, D. Branton, Plasma membrane: rapid isolation and exposure of the cytoplasmic surface by use of positively charged beads. *Science* **195**, 302–304 (1977).

35. L. P. Ferreira, V. M. Gaspar, J. F. Mano, Bioinstructive microparticles for self-assembly of mesenchymal stem Cell-3D tumor spheroids. *Biomaterials* **185**, 155–173 (2018).

36. Z. Zhang, J. Ren, W. Dai, H. Zhang, X. Wang, B. He, Q. Zhang, Fast and dynamic mapping of the protein corona on nanoparticle surfaces by photocatalytic proximity labeling. *Adv. Mater.* **35**, e2206636 (2023).

37. S. Li, J. Zhang, J. He, W. Liu, Y. Wang, Z. Huang, H. Pang, Y. Chen, Functional PDMS elastomers: Bulk composites, surface engineering, and precision fabrication. *Adv. Sci.* **10**, e2304506 (2023).

38. A. A. Popova, T. Tronser, K. Demir, P. Haitz, K. Kuodyte, V. Starkuviene, P. Wajda, P. A. Levkin, Facile one step formation and screening of tumor spheroids using droplet-microarray platform. *Small* **15**, e1901299 (2019).

39. V. Z. Beachley, M. T. Wolf, K. Sadtler, S. S. Manda, H. Jacobs, M. R. Blatchley, J. S. Bader, A. Pandey, D. Pardoll, J. H. Elisseeff, Tissue matrix arrays for high-throughput screening and systems analysis of cell function. *Nat. Methods* **12**, 1197–1204 (2015).

40. A. Martínez-Val, K. Fort, C. Koenig, L. Van der Hoeven, G. Franciosa, T. Moehring, Y. Ishihama, Y.-J. Chen, A. Makarov, Y. Xuan, J. V. Olsen, Hybrid-DIA: Intelligent

data acquisition integrates targeted and discovery proteomics to analyze phospho-signaling in single spheroids. *Nat. Commun.* **14**, 3599 (2023).

41. A. I. Minchinton, I. F. Tannock, Drug penetration in solid tumours. *Nat. Rev. Cancer* **6**, 583–592 (2006).

42. C. F. Rodríguez, V. Quezada, P. Guzmán-Sastoque, J. C. Orozco, L. H. Reyes, J. F. Osma, C. Muñoz-Camargo, J. C. Cruz, A mathematical phase field model predicts superparamagnetic nanoparticle accelerated fusion of HeLa spheroids for field guided biofabrication. *Sci. Rep.* **15**, 19765 (2025).

43. M. S. Huang, F. Christakopoulos, J. G. Roth, S. C. Heilshorn, Organoid bioprinting: from cells to functional tissues. *Nat. Rev. Bioeng.* **3**, 126–142 (2025).

44. V. A. Parfenov, Y. D. Khesuani, S. V. Petrov, P. A. Karalkin, E. V. Koudan, E. K. Nezhurina, F. D. Pereira, A. A. Krokhmal, A. A. Gryadunova, E. A. Bulanova, I. V. Vakhrushev, I. I. Babichenko, V. Kasyanov, O. F. Petrov, M. M. Vasiliev, K. Brakke, S. I. Belousov, T. E. Grigoriev, E. O. Osidak, E. I. Rossiyskaya, L. B. Buravkova, O. D. Kononenko, U. Demirci, V. A. Mironov, Magnetic levitational bioassembly of 3D tissue construct in space. *Sci. Adv.* **6**, eaba4174 (2020).

45. L. Gu, S. Jiang, X. Xu, J. Wang, F. Xu, H. Fan, J. Shang, K. Liu, U. Demirci, P. Chen, Size- and density-dependent acoustic differential bioassembly of spatially-defined heterocellular architecture. *Biofabrication* **15**, 015019 (2023).

46. T. Kamperman, N. G. A. Willemen, C. Kelder, M. Koerselman, M. Becker, L. Lins, C. Johnbosco, M. Karperien, J. Leijten, Steering stem cell fate within 3D living

composite tissues using stimuli-responsive cell-adhesive micromaterials. *Adv. Sci.* **10**, e2205487 (2023).

47. T. Fang, H. Zhang, Y. Xie, X. Li, X. Liu, Z. Wang, Y. Xue, X. Xia, Z. Wang, T. Lei, R. Lin, W. Shen, B. Wu, Y. Chen, Y. Du, X. Chen, Z. Yin, Centimeter-Scale self-assembling tendon organoids drive tissue regeneration. *Adv. Sci.* **12**, e09453 (2025).

48. M. Lei, J. Li, X. Ren, H. Xie, P. Li, F. Qi, J. Shi, X. Cai, Y. Liu, P. Chen, X. Feng, L. Xia, F. Wang, H. Li, M. Guo, Y. Zhang, B.-F. Liu, Y. Li, Tumor-adipose assembloids reveal cell-fate-transition-triggered multistage collective invasions. *Cell Syst.* **16**, 101353 (2025).

49. S. Toda, L. R. Blauch, S. K. Y. Tang, L. Morsut, W. A. Lim, Programming self-organizing multicellular structures with synthetic cell-cell signaling. *Science* **361**, 156–162 (2018).

50. D. Bhattaram, K. Golestan, X. Zhang, S. Yang, Z. Gong, S. L. Brody, A. Horani, V. A. Webster-Wood, A. B. Farimani, X. Ren, AggreBots: Configuring CiliaBots through guided, modular tissue aggregation. *Sci. Adv.* **11**, eadx4176 (2025).

51. Y. Perez-Riverol, A. Csordas, J. Bai, M. Bernal-Llinares, S. Hewapathirana, D. J. Kundu, A. Inuganti, J. Griss, G. Mayer, M. Eisenacher, E. Pérez, J. Uszkoreit, J. Pfeuffer, T. Sachsenberg, Ş. Yılmaz, S. Tiwary, J. Cox, E. Audain, M. Walzer, A. F. Jarnuczak, T. Ternent, A. Brazma, J. A. Vizcaíno, The PRIDE database and related tools and resources in 2019: Improving support for quantification data. *Nucleic Acids Res.* **47**, D442–D450 (2019).

52. L. Drakhlis, S. Biswanath, C.-M. Farr, V. Lupanow, J. Teske, K. Ritzenhoff, A. Franke, F. Manstein, E. Bolesani, H. Kempf, S. Liebscher, K. Schenke-Layland, J. Hegermann, L. Nolte, H. Meyer, J. de la Roche, S. Thiemann, C. Wahl-Schott, U. Martin, R. Zweigerdt, Human heart-forming organoids recapitulate early heart and foregut development. *Nat. Biotechnol.* **39**, 737–746 (2021).

53. M. Prondzynski, P. Berkson, M. A. Trembley, Y. Tharani, K. Shani, R. H. Bortolin, M. E. Sweat, J. Mayourian, D. Yucel, A. M. Cordoves, B. Gabbin, C. Hou, N. J. Anyanwu, F. Nawar, J. Cotton, J. Milosh, D. Walker, Y. Zhang, F. Lu, X. Liu, K. K. Parker, V. J. Bezzerides, W. T. Pu, Efficient and reproducible generation of human iPSC-derived cardiomyocytes and cardiac organoids in stirred suspension systems. *Nat. Commun.* **15**, 5929 (2024).

54. K. K. Moncal, S. Yaman, N. G. Durmus, Levitational 3D bioassembly and density-based spatial coding of levitoids. *Adv. Funct. Mater.* **32**, 2204092 (2022).
